# The ubiquitous P(*o*-tol)_3_ ligand promotes formation of catalytically-active higher order palladacyclic clusters†

**DOI:** 10.1039/d4sc05346j

**Published:** 2024-10-23

**Authors:** David R. Husbands, Theo Tanner, Adrian C. Whitwood, Neil S. Hodnett, Katherine M. P. Wheelhouse, Ian J. S. Fairlamb

**Affiliations:** a Department of Chemistry, University of York Heslington York YO10 5DD UK ian.fairlamb@york.ac.uk; b Medicine Development & Supply, GSK Medicines Research Centre Gunnels Wood Road, Stevenage Hertfordshire SG1 2NY UK

## Abstract

The Herrmann–Beller catalyst, Pd[(C^P)(μ_2_-OAc)]_2_, is readily formed by reaction of the cyclic trimer of ‘Pd(OAc)_2_’ with P(*o*-tol)_3_. In the presence of hydroxide, Pd(C^P)(μ_2_-OAc)]_2_ converts to [Pd(C^P)(μ__2__-OH)]_2_. Here, we report how this activated Pd precatalyst species, and related species, serve as a conduit for formation of higher order Pd_*n*_ clusters containing multiple cyclopalladated P(*o*-tol)_3_ ligands. The catalytic competency of a Pd_4_-palladacyclic cluster is demonstrated in an arylated Heck cross-coupling, which is comparable to the base-activated form of Herrmann's catalyst, namely [Pd(C^P)(μ_2_-OH)]_2_. The findings show that ‘simple’ ubiquitous phosphine ligands can promote higher order Pd speciation, moving beyond well-known phosphine-ligated Pd_1_ and Pd_2_ complexes. The findings challenge the status quo in the field, in that phosphine ligands can ligate higher order Pd_*n*_ species which are catalytically competent species in cross-coupling reactions.

## Abstract 2

Higher order palladacyclic Pd_*n*_ clusters, containing tri-*ortho*-tolyl phosphine, are active cross-coupling (pre)catalysts.

## Graphical abstract

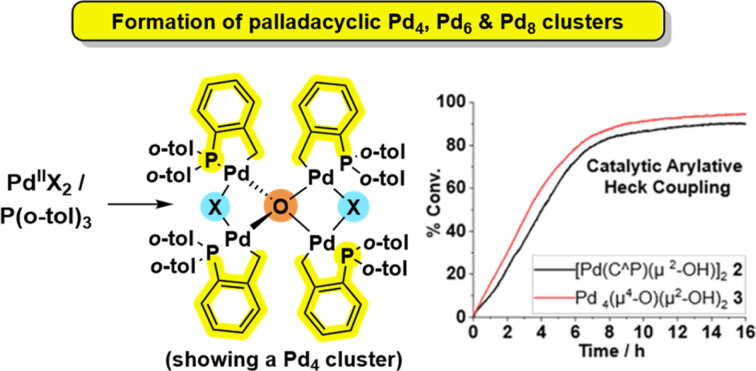

## Introduction

Tri-*ortho*-tolyl phosphine, P(*o*-tol)_3_, is a bench-stable phosphine ligand which is widely applied in some of the most challenging Pd-catalyzed cross-coupling reactions. P(*o*-tol)_3_ has an unusually large ligand cone angle (194°)^1^ and facile ability to undergo cyclometallation, especially at Pd^II^.^2^ Reaction screening campaigns often show that P(*o*-tol)_3_ effectively competes with specialist ‘designer’ electron-rich, sterically bulky phosphines.^3^ Of the many interesting applications of the P(*o*-tol)_3_ ligand, perhaps one of the most curious and interesting findings was deuteration of its *ortho*-methyl substituents influences branched/linear isomer product ratios in the Suzuki–Miyaura cross-couplings (SMCCs) of non-activated Csp^3^-boronic acids with aryl halides, an outcome that is not readily explained with simple Pd^0^(P(tol)_3_)_*n*_ catalyst models.^4^ Indeed, the related ‘simpler’ ligand, PPh_3_, can form multiple catalyst species, including higher order Pd_3_ clusters and nanoparticles.^5^ Furthermore, neutral PPh_3_ can be converted to anionic PPh_2_ ligands in Pd^II^ pre-catalyst activation processes.^6^ Out of curiosity emerges the question whether P(*o*-tol)_3_ can engage in similar ligand promiscuity to PPh_3_, influencing downstream Pd speciation and potentially different reaction outcomes.

**Fig. 1:**
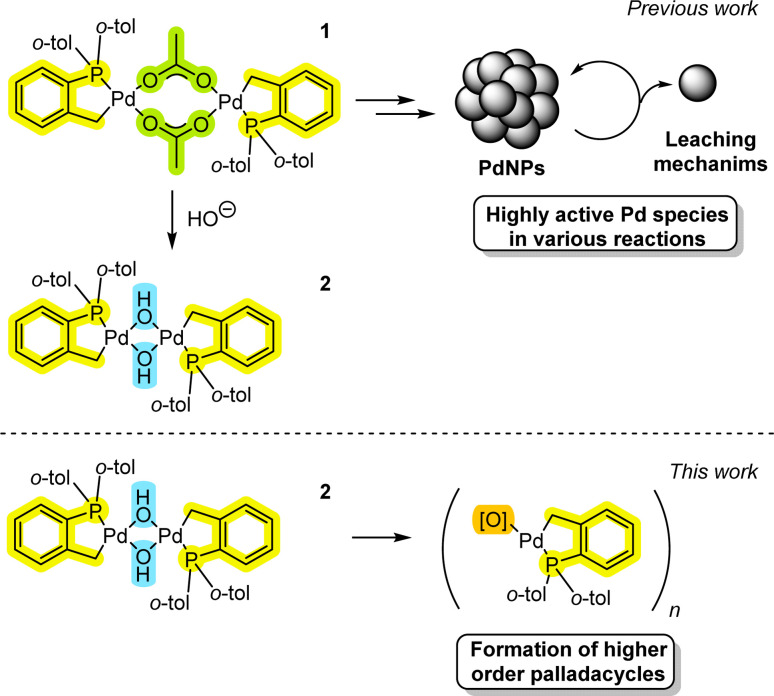
Highlighting the previous work: the historical findings summarized for 1 and recent implication of hydroxo-bridged species 2 in SMCC reactions. This work, revealing the hidden complexity and formation of higher order palladacycles involving P(*o*-tolyl)_3_.

Herrmann–Beller palladacycle [Pd(C^P)(μ_2_-OAc)]_2_1 is particularly effective for high temperature arylative Heck reactions,^7^ allowing for ultra-low Pd-catalyst loadings (TONs up to 1 × 10^6^).^8^ Application of the Pd(OAc)_2_/P(*o*-tol)_3_ pre-catalyst system is also effective (forming 1*in situ*) for an eclectic array of transformations (Fig. 1).^9^ Various mechanistic proposals^8,10^ have been put forward for these pre-catalyst systems, from higher order Pd nanoparticles involving low-order Pd leaching, through to the operation of higher oxidation state Pd^IV^ species.

Despite the mechanistic conundrums^11^ involving the study of Pd^II^ pre-catalyst systems, it is evident that there is an inherent complexity meriting deeper investigation. Indeed, in our recent work we have found that a stable [Pd(C^P)(μ__2__-OH)]_2_2 palladacycle is readily formed from the reaction of 1 with hydroxide base, giving a highly active Pd catalyst system for SMCC reactions under mild conditions.^12^ The facile formation and stability of 2 supports the water-assistive activation of 1 required for arylative Heck reactions.^13^ Our previous findings complement findings on SMCC reaction mechanisms, where intermediate ‘Pd^II^–OH’ species have been implicated in productive catalysis.^14^

Here, we report how 2 and related derivatives can form higher order Pd_*n*_ clusters (*n* = 4, 6 and 8), under different reaction conditions, and show that Pd_4_-cluster 3 is a competent pre-catalyst for Heck reactions. It is clear from our studies that the P(*o*-tol)_3_ ligand readily stabilizes higher order Pd^II^ palladacyclic cluster species, while undergoing P–C bond cleavage under specific conditions. Furthermore, palladacycles derived from P(*o*-tol)_3_ are able to react with cross-coupling reagents.

## Results and discussion

**Fig. 2:**
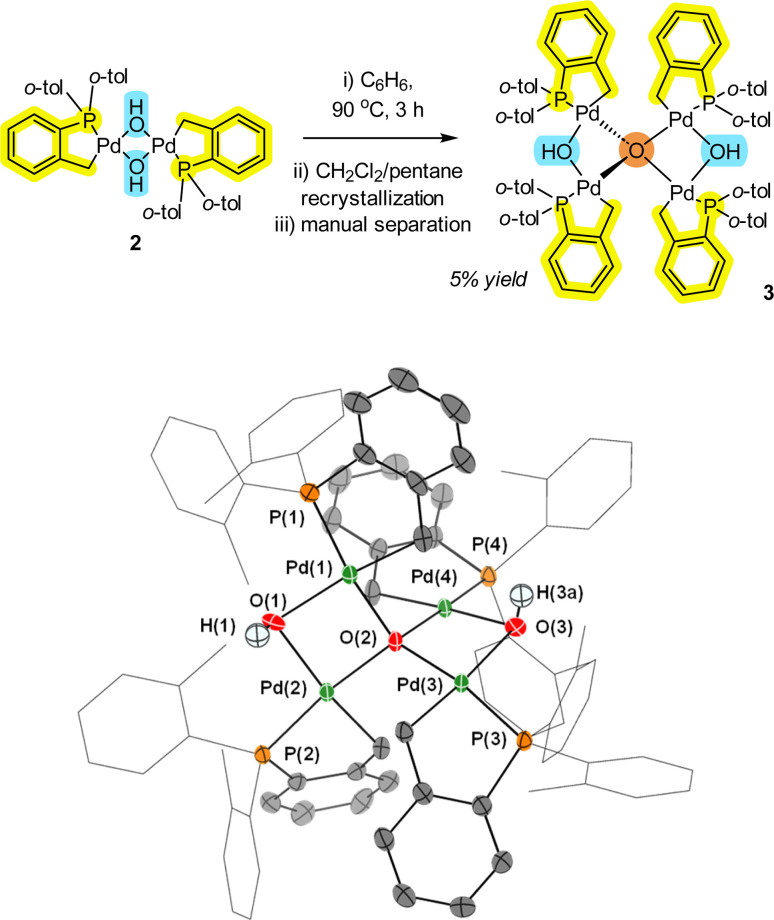
Formation of Pd_4_ cluster 3 from 2. The single crystal XRD structure of 3 is shown with selected ellipsoids (set to 50%) for clarity (all H-atoms with the exception of the bridging-hydroxo ligands have been hidden from view). Selected interatomic lengths/Å: Pd1–C1 = 2.032(4); Pd1–P1 = 2.1841(9); Pd1–O1 = 2.202(2); Pd1–O2 = 2.070(2); Pd1–Pd2 = 3.1785(4). Selected interatomic angles/°: P1–Pd1–C1 = 84.52(12); P1–Pd1–O1 = 102.74(18); Pd1–O1–Pd2 = 92.89(10); Pd1–O2–Pd2 = 99.66(10); Pd3–O2–Pd4 = 97.35(9)>.

While the [Pd(C^P)(μ_2_-OH)]_2_2 pre-catalyst is an air and water stable complex,^12^ under dehydrating or limited water conditions, the complex readily loses H_2_O to form a new species, which was determined to be Pd_4_(μ_4_-O)(μ_2_-OH)_2_ cluster 3 by single crystal X-ray diffraction (XRD) analysis (Fig. 2). In solution, 2 exists as a mixture of *trans* and *cis* isomers (2 : 1 ratio), but only as the *trans*-isomer in the solid-state. The act of removing solvent from a pure sample of 2 in solution is enough to form small quantities of 3 (observed as a color change from a cream to a yellow solid). Due to steric limitations, only the minor *cis* isomer of 2 can dimerize (inferred by the structure of 3). One of the unusual structural features of 3 is a four-coordinate tetrahedral oxygen (μ_4_-O) atom connecting four separate Pd atoms. There are two Pd–O bonds that are shorter (2.070(2) and 2.070(2) Å) and two Pd–O bonds that are marginally longer (2.089(2) and 2.088(2) Å). The former represents the anionic component to the μ_4_-O-bond arrangement, whereas the latter are dative in nature. The Pd–OH bond lengths (2.202(2) Å) are significantly longer than the Pd–O bonds (2.070(2) Å). The Pd–OH bond distances are also longer than seen in 2 {for 2, Pd–OH 2.115(2)}, so there appears to be an element of ‘relaxation’ in the structure of 3 compared with 2.

As the synthesis of 3 involves refluxing 2 at 90 °C for 3 h, it is of particular note that the stability of this palladacycle is maintained at elevated temperatures. Interestingly, Pd_4_ cluster 3 is similar to a structure formed involving reactions of Pd_3_(OAc)_6_ as reported by Bedford *et al.*^15^ namely [{Pd_3_(OAc)_5_}_2_(μ_4_-O)]. Under water-limiting conditions (note: not water-free) this demonstrates that aggregation of the Pd^II^ pre-catalyst species is possible for both 1 and ubiquitous [Pd_3_(OAc)_6_] *vide infra*. Similar higher order Pd_*n*_ clusters have been implicated (by kinetic studies) in arylative Pd-catalyzed cyanation reactions mediated by [Pd(OAc)_2_(HNR_2_)_2_] pre-catalysts.^16^ Our findings therefore make a connection between non-phosphine and phosphine-ligated Pd_*n*_ clusters.

In a separate reaction we found that [Pd(C^P)(μ_2_-Cl)]_2_4 (see ESI† for synthesis), when reacted with AgC_6_F_5_ at room temperature, formed a small number of bright yellow single crystals (compared to red crystals for target compound 6).

**Fig. 3:**
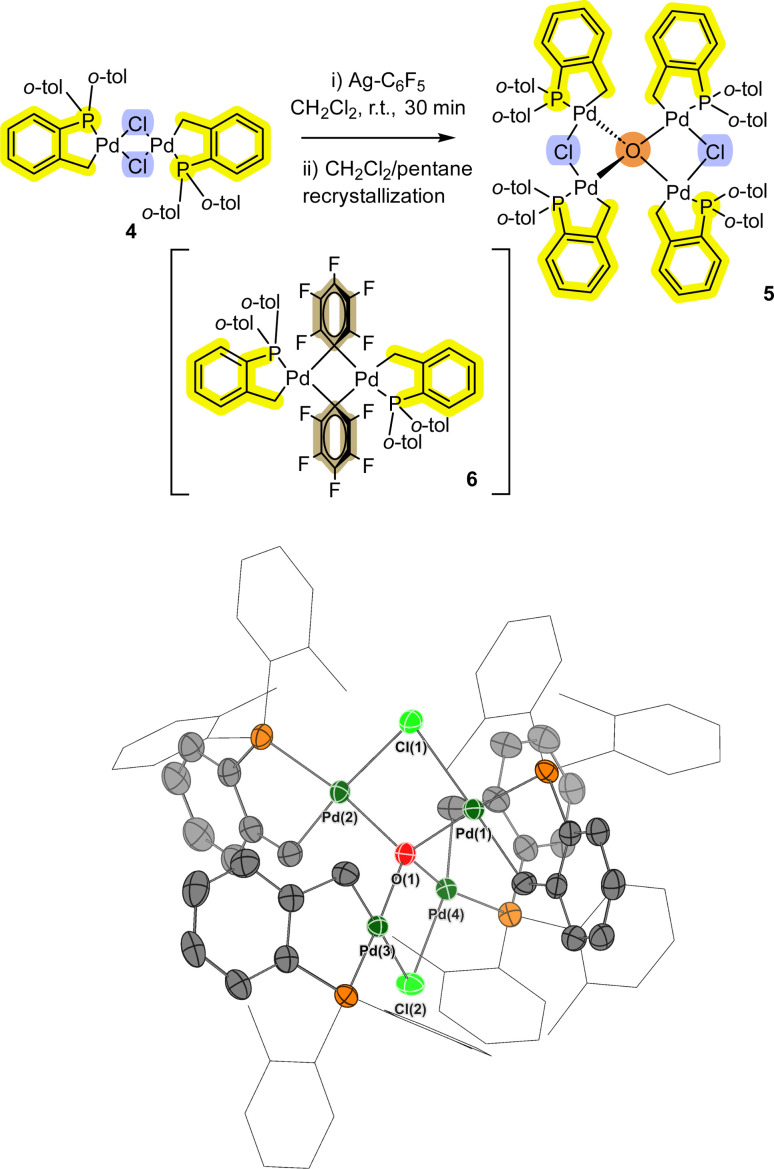
Formation of Pd_4_ cluster 5 from palladacycle 4. The single crystal XRD structure of 5 is shown with selected ellipsoids (set to 50%) for clarity (all H-atoms, with the exception of the bridging-hydroxo ligands, have been hidden from view). Selected interatomic lengths/Å: Pd1–Cl1 = 2.4737(8), Pd1–O1 = 2.101(2), Pd1–P1 = 2.2093(9), Pd1–C1 = 2.052(3). Selected interatomic angles/°: Pd1–Cl1–Pd2 = 84.35(3), Pd3–Cl2–Pd4 = 84.67(3), Pd1–O1–Pd4 = 103.46(9), Pd2–O1–Pd3 = 113.65(10).

Interestingly, pre-heating with AgC_6_F_5_ at 100 °C under vacuum to remove residual EtCN from the synthesis, and changing the reaction solvent from DCM to THF results in the exclusive formation of the transmetallation product 6 and its monomer.^12^ Single crystal XRD analysis of the yellow crystals showed that a related Pd_4_ cluster 5 was formed, which is the chloro-analogue of 3 (Fig. 3). The interesting implication here is that palladacycle compounds containing four Pd atoms can readily form, templated by a (μ_4_-O)-bonding arrangement (the O is likely from adventitious oxygen in the reaction vessel), such as seen earlier for non-phosphine ligated Pd_3_ cluster [{Pd_3_(OAc)_5_}_2_(μ_4_-O)].^15^

**Fig. 4:**
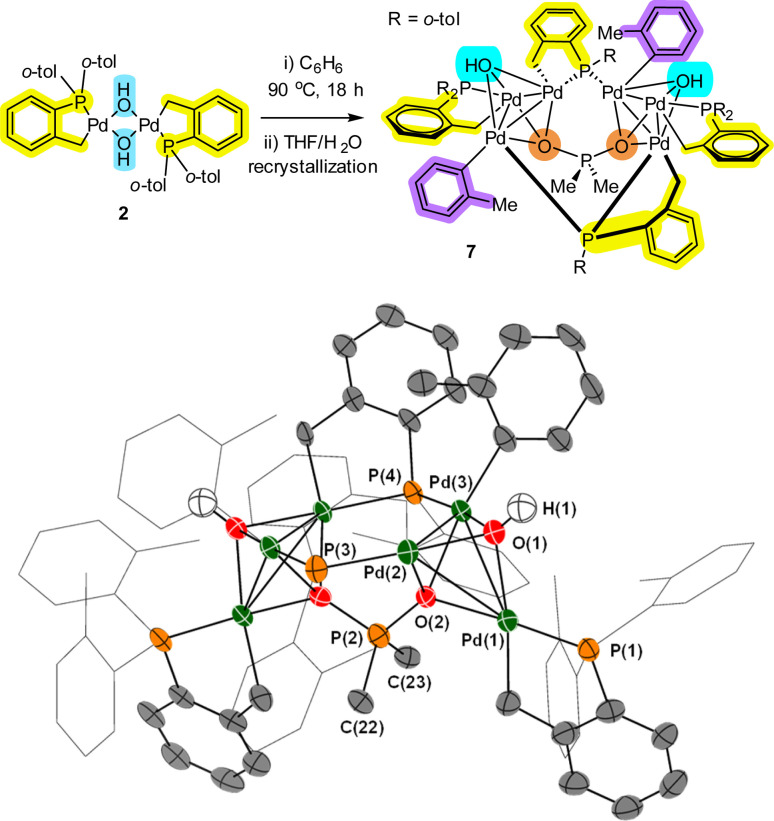
Formation of Pd_6_ cluster 7 from palladacycle 2. The single crystal XRD structure of 7 is shown with selected ellipsoids (set to 50%) for clarity (all H-atoms with the exception of the bridging-hydroxo ligands have been hidden from view). Selected bond lengths (Å) and angles (°); Pd1–P1 = 2.201(2); Pd1–O1 = 2.141(6); Pd1–Pd2 = 3.0422(9); Pd2–P3 = 2.218(2); Pd3–P4 = 2.238(2); O2–P2 = 1.631(6); P2–C22 = 1.878(9); Pd1–Pd2–Pd3 = 59.30(2); O1–Pd1–O2 = 70.5(2); Pd1–O2–P2 = 129.7(3); C22–P2–C23 = 109.7(4).

Returning to Pd_4_ cluster 3, on one occasion under specific reaction conditions we obtained single crystals of a new species which was determined by XRD analysis to be Pd_6_ cluster 7 (Fig. 4). In this case, Pd[(C^P)(μ_2_-OH)]_2_2 was refluxed in benzene, filtered, crystallized from THF/water at 5 °C, which was exposed to air *via* a bleed needle for a period of 2 months. Structural features of note are: (1) a planar Pd_3_ motif with capping OH groups; (2) a bridging P(*o*-tol)_2_ palladacycle which has had one *o*-tol group cleaved onto a neighboring Pd atom; (3) the appearance of an ionic O_2_PMe_2_ group which implies that two *o*-tol methyl substituents have been cleaved from a P(*o*-tol)_3_ ligand.

Alternative possibilities for the O_2_PMe_2_ anion were fully considered. Our computational calculations (using DFT methods) suggest that the O_2_PMe_2_ anion is the most likely (compared with other plausible anions to O_2_SMe_2_, O_2_PO_2_ and O_2_P{(OH)_2_}) (see ESI†). Clearly the formation of Pd_6_ cluster 7 has involved at least two P–C bond activations, with some palladacyclic motifs remaining intact, an observation that is testament to the stabilization conferred by cyclopalladation. The formation of “Pd(*o*-tol)” corner motifs is particularly noteworthy. It is interesting to note that Sunada *et al.* reported a nuclearity expansion in Pd_*n*_ clusters triggered by a migrating phenyl group from cyclooligosilanes, a process that is reminiscent of that observed in the formation of 7.^17^

Along with Pd_4_ clusters 3 and 5, Pd_6_ cluster 7 demonstrates that under water-limiting conditions that higher order Pd^II^ clusters can be formed, which is relevant to catalytic cross-coupling reaction conditions (*vide infra*).

**Fig. 5:**
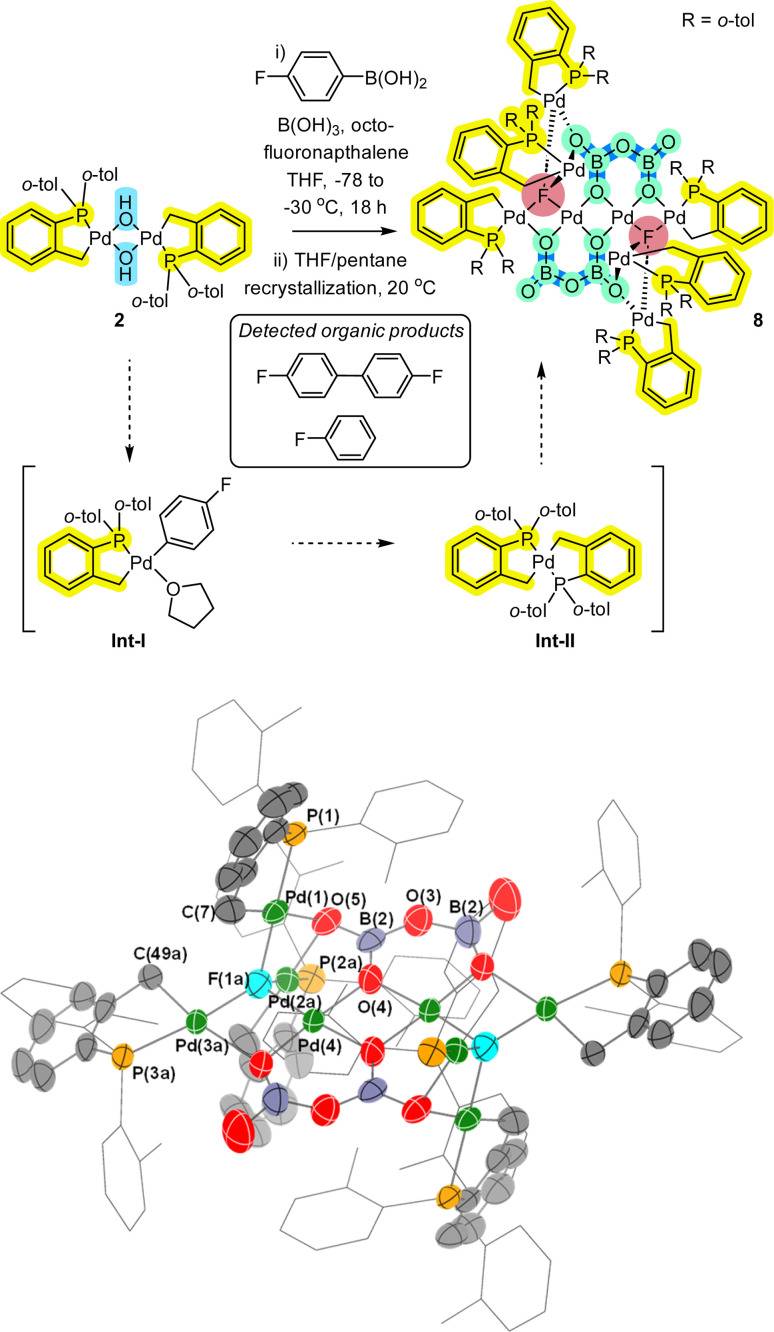
Formation of Pd_8_ cluster 8 from palladacycle 2 (the Pd atoms have been formatted in bold for clarity). The single crystal XRD structure of 8 is shown with selected ellipsoids (set to 50%) for clarity (all H-atoms have been hidden from view). Selected interatomic lengths/Å: P1–Pd1 = 2.1856(10); Pd1–C7 = 2.024(5); Pd1–F1A = 2.02(3); Pd2A–F1A = 2.10(2); Pd3A–F1A = 2.06(3); Pd4–F1A = 2.04(3); Pd4–O1 = 1.991(3); Pd4–O4 = 1.980(3). Selected interatomic angles/°: O4–Pd4–O1 = 95.56(13); Pd1–F1A–Pd2A = 98.7(10); Pd1–F1A–Pd4 = 97.7(13); Pd2A–F1A–Pd3A = 131.2(14); Pd1–F1A–Pd3A = 120.6(13); P1–Pd1–C7 = 83.77(14).

Next, in a study involving reaction of 2 by aryl boronic acids,^12^ further complexity involving higher order Pd_*n*_ species possessing palladacyclic motifs was revealed. Reaction of 2 with arylboronic acid 9 (2 equiv.), in the presence of excess boric acid (4 equiv.) and octafluoronaphthalene (1 equiv.) showed the formation of a broad lump between *δ* 30–40 ppm by ^31^P NMR spectroscopic analysis, accounting for 93% of the observed ^31^P signals (see ESI†). This indicates that other Pd species are likely formed. Upon addition of 4-fluorobromobenzene, no reaction was observed, implying these species contain oxidised Pd^II^ centres *vide infra* (noting that Pd^0^ would undergo an oxidative addition). As such the crude reaction mixture was set-up for crystallisation (*i.e.* using THF/pentane, slow vapor diffusion over 17 months). Single crystal XRD analysis revealed the formation of a remarkable Pd_8_ cluster 8 (Fig. 5). While there is disorder in the structure, a suitable model was constructed (see ESI,† Section 6). Firstly, Pd_8_ cluster 8 possesses a distinctive bridging μ_4_-F atom. Pd_8_ cluster 8 also possesses bridging oxygens from two stabilizing-boronate anions. Therefore, the Pd_8_ cluster core motif in 8 carries an overall +2 charge. Two of the central Pd atoms are not cyclopalladated, leaving 6 palladacyclic motifs within this unique Pd_8_-cluster.

Given the changes seen in both ^31^P and ^19^F NMR spectral data we postulate the involvement of intermediates (Int-I and Int-II),^12^ in the transformation leading to the formation of 8. It is pertinent to mention the formation of organic by-/side-products, fluorobenzene (formed by protodeborylation) and 4,4′-difluoro-biphenyl (formed by homocoupling of the 4′-fluorophenyl boronic acid). The appearance of fluoride ion in 8 suggests an abstraction of fluoride from either octofluoronaphthalene, *p*-F–C_6_H_4_–B(OH)_2_ or other fluoride-containing by- or side-product.

### Catalysis

**Fig. 6:**
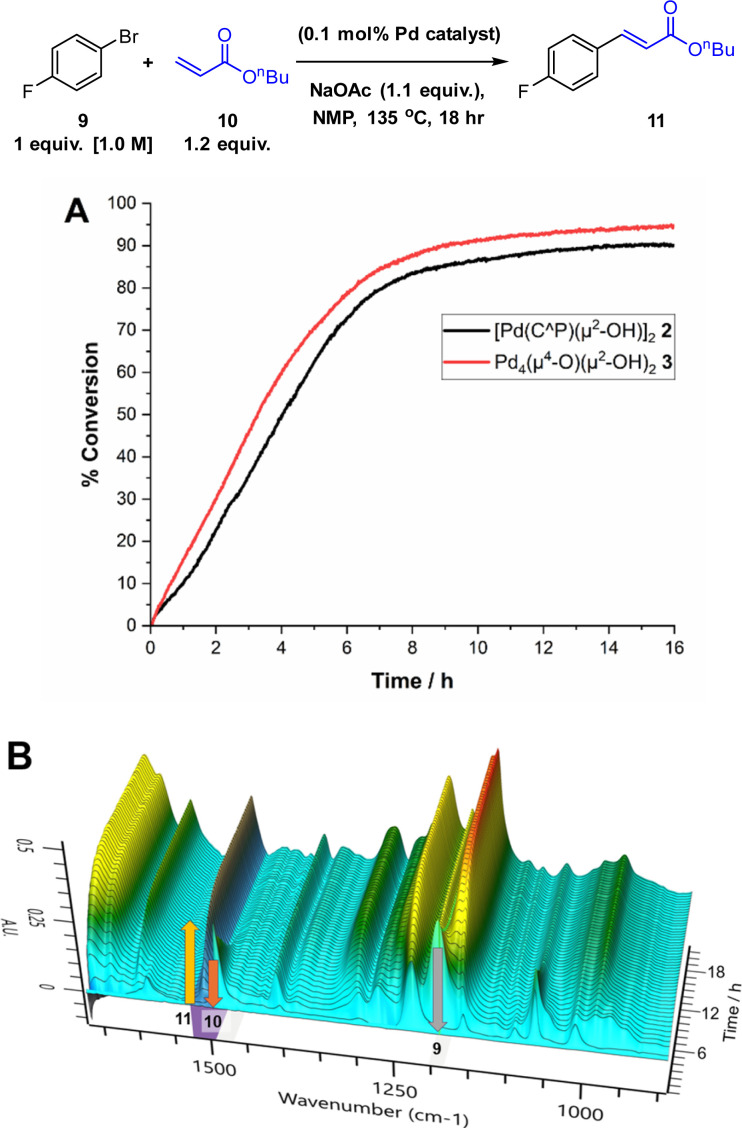
Model Heck arylation reaction to test Pd_4_ cluster 3 against intermediate dinuclear Pd species 2. (A) Kinetic profiles showing the formation of *n*-butyl 4-fluorocinnamate 11 (product appearance at 1509 cm^−1^). (B) Temporal IR spectral changes underpinning the kinetic changes shown in A. Formation of 11 at 1509 cm^−1^ and loss of 1-bromo-4-fluorobenzene 10 at 1484 cm^−1^ and *n*-butyl acrylate 9 at 1190 cm^−1^ (reaction mixture compared with reference standards in NMP solvent).

We chose the Heck reaction of an aryl halide 9 with terminal alkene 10 (forming 11, as a single *E*-isomer) to benchmark the catalytic performance of Pd_4_ cluster 3 relative to known^12^ dinuclear complex 2 (Fig. 6). This class of catalytic reaction is underpinned by a significant body of mechanistic information involving palladacyclic pre-catalysts, making it the ideal candidate in which to assess the catalytic competency of 3.^13^ The catalytic arylative Heck reaction was further amenable to reaction monitoring using *in situ* IR (Mettler-Toledo RIR15 with a flexible AgX diamond probe), which allows the solution changes in the IR stretching bands of the different reaction components (see Fig. 6 caption details) to be monitored in real-time (note: the second derivative was used to process the raw spectral data, in-keeping with the kinetic analyses conducted on Suzuki–Miyaura cross-couplings as part of an extended but related study examining the catalytic behaviour of 2).^12^

Using 2 as the precatalyst, the reaction 9 + 10 → 11 reaches 80% conversion (to product 11) after 8 h (comparable to ^1^H NMR spectroscopic analysis), providing a good window to compare the catalytic performance of Pd_4_ cluster 3. Under anhydrous reaction conditions, single crystals of 3 exhibited catalytic activity commensurate with 2, as shown by the similar rates of product formation and shape of the product (11) evolution curves. In the presence of water (a necessary additive, likely generating 2*in situ*^12^) the Herrmann–Beller catalyst 1 exhibits similar catalytic activity to 2 and 3 (see ESI†). The results taken together allow us to make a link between 1, 2 and 3 for the Heck reaction 9 + 10 → 11 (note ∼2% homocoupled product, 4,4′-difluoro-biphenyl, was also observed by NMR spectroscopic analysis).

The overall outcome from these comparative catalytic reactions support the assertion that under water-limiting conditions (<50 ppm H_2_O at 0.1 mol% Pd catalyst loading), both Pd_2_ complex 2 and Pd_4_ cluster 3 are stepping-stones to higher order Pd_*n*_ species. PdNP catalysed arylative Heck reactions are well known.^8,13*d*^ Thus, the catalytic competency of 3 provides evidence that P(*o*-tol)_3_ based Pd pre-catalysts can bridge the gap to formation of PdNPs *via* this route involving higher order Pd_*n*_ cluster species.

### Further discussion regarding Pd_4_ cluster formation

**Fig. 7:**
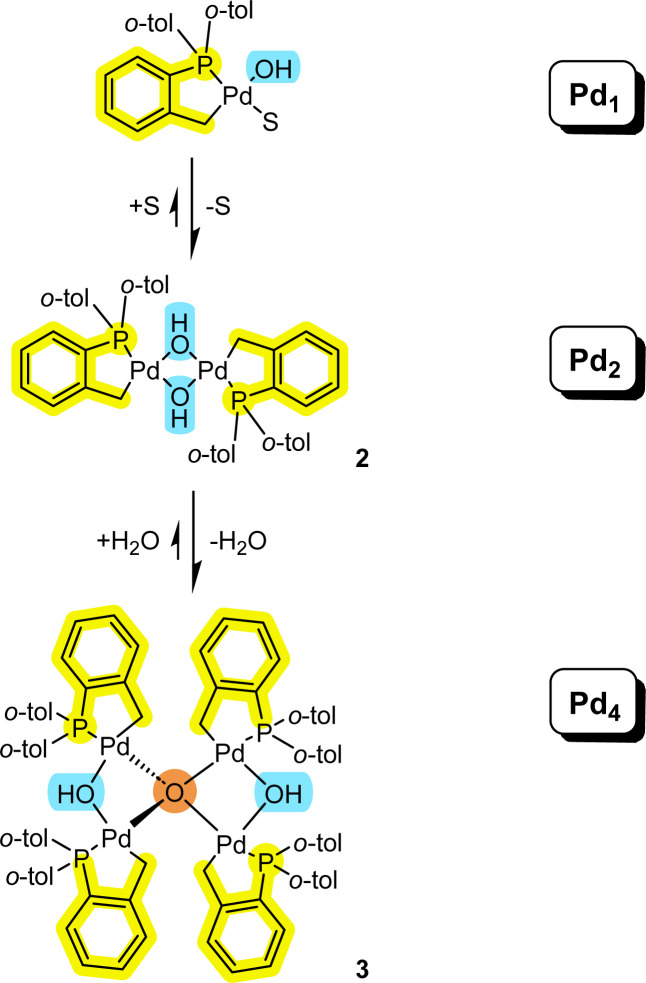
Connecting mononuclear palladacyclic species with the higher order Pd_4_ type cluster 3 (S = solvent).

It is pertinent to reflect on the formation of the higher order Pd_4_ cluster 3 and how it connects to Pd_*n*_ species of lower nuclearity (Fig. 7). It is evident that both solvent and water need to be considered in equilibrium events described in Fig. 7. The equilibrium balance will be dependent on the solvent type and concentration of water. Palladacyclic dinuclear complexes, such as 2, are in equilibrium with mononuclear Pd_1_ complexes.^18^ The equilibrium position will be expected to change when employing polar aprotic solvents and where there are heterocyclic systems present (possessing strong 2-electron nitrogen donor atoms, like pyridine,^18^ for example).

The formation of Pd_*n*_ clusters 7 and 8 is quite remarkable – they are ‘a needle in a haystack’ type result. That said, their formation demonstrates that the P(*o*-tol)_3_ ligand can be chemically-fragmented, templating and stabilising these higher order Pd_*n*_ species. It is an important serendipitous result.

## Conclusions

The ubiquitous P(*o*-tol)_3_ ligand is capable of templating and stabilizing higher order Pd_*n*_ clusters (where *n* = 4, 6 and 8). Our research findings challenge the status quo – higher order palladacycles are readily accessible under different reaction conditions and are stable and catalytically relevant. Pd_4_ cluster 3 was found to exhibit catalytic activity similar to both 1 and 2. Generally, the P(*o*-tol)_3_ ligand has unique electronic and steric properties, which has been widely applied in Pd-catalysed cross-coupling reactions since their discovery in the 1960s/1970s.^1^ It is common to see in the literature simple models used to describe ‘Pd–P(*o*-tol)_3_’ catalyst species, including ligand parameterization methodologies.^2^ Our findings show that higher order species need to be factored into more complete models for catalytic reaction manifolds, particularly with reaction complexity^19^ in mind. Our findings further add to related observations made for the aggregation of Pd_*n*_ species, containing the Trost Modular ligand, made by Lloyd-Jones *et al.* in asymmetric allylic alkylation reactions.^20^ Formation of higher order palladacyclic clusters also provides clues to pathways leading to the generation of PdNPs.

## Data availability

We have provided a comprehensive electronic version of ESI.† The raw spectroscopic data (*e.g.* NMR fid files) and kinetic data (.csv files) will be uploaded to the York Research Database (the open data repository for the University of York) on publication of the manuscript. We can be contacted by email for processed data files (*e.g.* MNova NMR files and Excel/Origin files), although we are unable to provide access to the licensed commercial software. The cif files for the single crystal XRD structures are available through the Cambridge Crystallographic Data Centre (CCDC).^21^

## Author contributions

N.·S.·H., K. M. P. W. and I. J. S. F. jointly conceived and designed the research project focusing on Herrmann's catalyst 1, securing iCASE funding from GSK and EPSRC (UKRI). D. R. H. and I. J. S. F. devised experiments, supported by input from N.·S. H., K. M. P. W. and D. R. H. conducted all of coordination chemistry and catalysis experiments described in the manuscript. I. J. S. F. and D. R. H. co-wrote the manuscript, with input provided by all authors. D. R. H. prepared the electronic ESI† document, with input from all authors. A. C. W. and T. T. were involved in the data collection and processing of single crystal X-ray diffraction data (A. C. W. further refined the XRD structural data following feedback by the expert X-ray crystallographic reviewer). The research results described in this manuscript are presented in the PhD thesis of D. R. H. (graduated 2024). N. S. H., K. M. P. W. and I. J. S. F. provided guidance during regular online/in-person meetings throughout D. R. H.‘s PhD studies. I. J. S. F. supervised the PhD studies of D. R. H. at the University of York.

## Conflicts of interest

There are no conflicts to declare.

## Supplementary Material

SC-OLF-D4SC05346J-s001

SC-OLF-D4SC05346J-s002

SC-OLF-D4SC05346J-s003
