# Supplementary material for: The ubiquitous P(o-tol)3 ligand promotes formation of catalytically-active higher order palladacyclic clusters
Source: Chem Sci. 2024 Oct 23;15(44):18627–33. doi: 10.1039/d4sc05346j (PMC11497114; doi:10.1039/d4sc05346j)
Supplement: SC-015-D4SC05346J-s002 [file SC-015-D4SC05346J-s002.pdf]

Supplementary Information (SI) for Chemical Science.
This journal is © The Royal Society of Chemistry 2024

Supporting Information for

The ubiquitous $P(o\text{-}tol)_3$ ligand promotes formation of catalytically active higher order palladacyclic clusters

David R. Husbands,[a] Theo Tanner,[a] Adrian C. Whitwood,[a] Neil S. Hodnett,[b] Katherine M. P. Wheelhouse[b] and Ian J. S. Fairlamb[a,*]

[a] Department of Chemistry, University of York, Heslington, York, YO10 5DD, United Kingdom. [b] Medicine Development & Supply, GSK, Gunnels Wood Road, Stevenage, Hertfordshire, SG1 2NY, United Kingdom

Table of Contents

1. General Information 2
  1.1 Compound Preparative Techniques 2
  1.2 Instrument Details and Methods for Compound Characterization 2
2. Experimental Details 4
  2.1 General Procedure: Heck Alkenylation Reaction 4
  2.2 Synthesis of Compounds 5
3. *In situ* IR Data – Methodology and Kinetic profiles 31
4. X-Ray Crystallography 36
5. NMR Spectral Data for Organic and Inorganic Compounds 45
6. Density Functional Theory (DFT) Calculations 60
  6.1 DFT Calculations – Hexamer Variant Optimised Structure Coordinates 60
7. References 73

1. General Information

1.1 Compound Preparative Techniques

Reagents and solvents were purchased from Merck, Fluorochem, Alfa Aesar, and Fisher and used without further purification. The purity of chemicals was confirmed by NMR spectroscopy prior to use.

1.2 Instrument Details and Methods for Compound Characterization

NMR spectra were obtained in the solvent indicated in the text below, using a Bruker AVIIIHD 600 Widebore instrument (600 MHz [^{1}H], 565 MHz [^{19}F], 243 MHz [^{31}P], 151 MHz [^{13}C]) or a Bruker AVIIIHD 500 instrument (500 MHz [^{1}H], 471 MHz [^{19}F], 203 MHz [^{31}P], 125 MHz [^{13}C]). Chemical shifts (δ) are reported in parts per million (ppm) and were referenced to the residual non-deuterated solvent of the deuterated solvent used; $CDCl_3$: δ ^{1}H = 7.26 ($C\mathit{H}Cl_3$) and ^{13}C = 77.16 ($\mathit{C}DCl_3$); DCM-d_2 : ^{1}H = 5.32 ($CD\mathit{H}Cl_2$) and ^{13}C = 54.0 ($\mathit{C}D_2Cl_2$); THF-d_8 : δ ^{1}H = 3.59 ($OC\mathit{H}_2CH_2$), ^{13}C = 67.57 ($O\mathit{C}H_2CH_2$), ^{1}H = 1.73 ($OCH_2C\mathit{H}_2$) ^{13}C = 25.37 ($OCH_2\mathit{C}H_2$); C_6D_6 : δ ^{1}H = 7.16 ($C_6D_5\mathit{H}$), ^{13}C = 128.06 (C_6D_6). Spectral data were typically collected at 298 K (25 °C), unless stated otherwise. All ^{1}H NMR signals are reported as they appear in the spectrum, which in some cases may lead to there being more protons reported than there are in the compound (according to HRMS). This is often due to residual solvent peaks (either from the NMR solvent or solvent trapped in the compounds during crystallization) overlapping in the aromatic region.

^{31}P NMR spectral data were collected with proton decoupling, unless otherwise stated. Chemical shifts for ^{31}P resonances were calibrated by externally referencing to 85% H_3PO_4 in H_2O (w/w). This was practically carried out by inserting a sealed, vacuum-dried capillary tube containing 85% H_3PO_4 in H_2O (w/w) into an NMR tube containing the sample of interest, collecting a ^{31}P NMR spectrum and setting the $H_3\mathit{P}O_4$ resonance to 0 ppm. ^{19}F spectral data were referenced in the same manner using α,α,α-trifluorotoluene (-63.72 ppm with respect to $CFCl_3$). All ^{13}C NMR spectra were obtained with ^{1}H decoupling. All NMR spectra were processed using MestReNova (MNova) software (v. 14).

HRMS ESI-MS spectra were measured using a Bruker Daltronics micrOTOF MS, Agilent series 1200LC with electrospray ionization (ESI) or on a Thermo LCQ using electrospray ionization, with <5 ppm error recorded for all HRMS samples. LIFDI (Liquid Injection Field Desorption Ionization) mass spectrometry was carried out using a JEOL AccuTOF GCx-plus instrument (JMS-T200GC), fitted with a probe produced by Linden CMS. The probe was equipped with 13 μm emitters on an AccuTOF. Alternatively, LIFDI-MS was carried out using a Waters GCT Premier MS Agilent 7890A GC instrument. Mass to charge ratios (*m/z*) are reported in Daltons. High resolution mass spectra (HRMS) are reported with <5 ppm error (ESI and LIFDI). For clarity, LIFDI data are reported for ^{106}Pd, the most abundant natural isotope of Pd, which is part of ‘exact mass’ values. LIFDI ions are reported as the radical cation $M^{+\bullet}$. ESI ions are reported as the $[M+H]^+$ cation,

unless a Na or K is present in the molecular formula, in which case the $[M+Na]^+$ or $[M+K]^+$ ion is being measured.

Infrared spectra were obtained using a Bruker ALPHA-Platinum FTIR Spectrometer with a platinum-diamond ATR sampling module. Far IR spectra were obtained using a Bruker Tensor 37 FTIR Spectrometer with a platinum-diamond ATR sampling module. Melting points were determined using a Stuart® SMP3 Melting Point machine. All Heck reaction kinetic experiments were followed using a Mettler Toledo React-IR spectrometer with a diamond probe. IR spectra were collected in real-time every 60 seconds between 4000 and 649 cm^{-1}, with a spectral resolution of 4 cm^{-1}. All reaction temperatures were measured independently from the heating bath using a TENMA 72-7715 electronic thermometer and thermocouple to ensure accurate reaction temperature regulation. All data analysis was carried out on raw, uncorrected data.

For single crystal X-ray crystallographic analysis details, please see Section 4.

2. Experimental Details

2.1 General Procedure: Heck Alkenylation Reaction

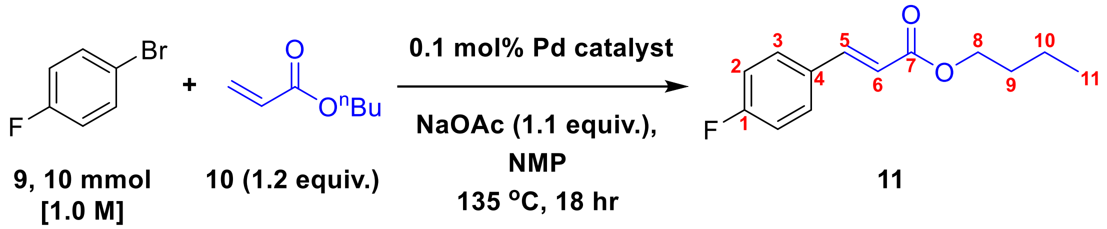

9, 10 mmol [1.0 M] **10 (1.2 equiv.)** **11**

An oven-dried three-necked 100 mL round bottom flask was fitted onto a Mettler-Toledo ReactIR probe (flexible silver halide diamond probe), fitted with a condenser. This was evacuated and backfilled with N_2 (three cycles). The flask was charged with NMP (10.0 mL, dried, degassed), then heated to 135 °C, at which point a solvent background IR spectrum was recorded. NaOAc (0.903 g, 11.0 mmol, 1.1 equiv., anhydrous) was added, followed by 1-bromo-4-fluorobenzene **9** (1.1 mL, 1.75 g, 10.0 mmol, 1.0 equiv., stored over molecular sieves), then catalyst (0.005 mmol, 0.1 mol% Pd) was added. The reaction was initiated by the addition of n-butyl acrylate **10** (1.539 g, 1.71 mL, 12.0 mmol, 1.2 equiv., stored over molecular sieves). The reaction progress was monitored by measuring the increase in absorbance at 1509 cm^{-1}, relating to the formation of n-butyl 4-fluorocinnamate **11**, and the decrease in absorbances at 1484 cm^{-1}, relating to the consumption of 1-bromo-4-fluorobenzene **9**, and 1190 cm^{-1}, relating to n-butyl acrylate **10**. To deconvolute the peaks and obtain reasonable quality data, the second derivative of the spectra was used. The reaction was halted when the peaks at these values reached a constant value, indicating completion. At the end of the reaction, a 0.5 mL aliquot of the reaction was analysed by ^{19}F NMR (20 s relaxation delay) to measure the conversion of each reaction. The final conversion was calculated by measuring the ratio of the diagnostic peaks by (^{19}F δ) -115.9 ppm for 1-bromo-4-fluorobenzene **9** and -109.8 ppm for n-butyl 4-fluorocinnamate **11**. The peak absorption data were imported into Excel after subtracting the NMP solvent reference spectrum. All kinetic traces (conversions) were normalised using the final reaction conversion (as calculated *vide supra*) and the value of an appropriate data point just before the aliquot was taken.

The identity of the product was confirmed by performing a reaction at half-scale in a Schlenk tube following the same procedure. The reaction mixture was extracted into EtOAc (3 x 20 mL), the organic layers were combined and washed with water (5 x 20 mL), dried ($MgSO_4$), filtered and the solvent removed *in vacuo*. The crude was purified by column chromatography (automated Combiflash, 12 g silica cartridge, 5% EtOAc in hexane), R_f = 0.34 (5% EtOAc in hexane)) to give the product **11** as a colourless oil (exclusively the *trans* isomer, 812 mg, 73% yield, using Herrmann-Beller palladacycle **1**). (Note: upon isolation, 2% of 4,4'-difluoro-biphenyl by-product co-eluted, which is visible in the ^1H and ^{19}F NMR as a trace impurity) ^1H NMR (500 MHz, $CDCl_3$, 298 K) δ 7.63 (d, *J* = 16.0 Hz, 1H, H-5), 7.54 – 7.47 (m, 2H, H-3), 7.10 – 7.03 (m, 2H, H-2), 6.36 (dd, *J* = 16.0, 0.6 Hz, 1H, H-6), 4.20 (t, *J* = 6.7 Hz, 2H, H-8), 1.73 – 1.63 (m, 2H, H-9), 1.48 – 1.38

(m, 2H, H-10), 0.96 (t, *J* = 7.4 Hz, 3H, H-11); ^{19}F NMR (470 MHz, C*D*Cl$_3$, 298 K) δ -109.85 (tt, *J* = 8.4, 5.4 Hz); ^{13}C NMR (126 MHz, C*D*Cl$_3$, 298 K) δ 167.1 (C-7), 164.0 (d, *J* = 251.3 Hz, C-1), 143.3 (C-5), 130.9 (d, *J* = 3.4 Hz, C-4), 130.0 (d, *J* = 8.5 Hz, C-3), 118.2 (d, *J* = 2.3 Hz, C-6), 116.1 (d, *J* = 22.0 Hz, C-2), 64.6 (C-8), 30.9 (C-9), 19.3 (C-10), 13.9 (C-11); HRMS (ESI$^+$) ($C_{13}H_{15}FO_2Na$)$^+$ *m/z* (calculated) 245.0948 (found) 245.0949, mass difference 0.3 ppm; FTIR (ATR): ṽ (cm^{-1}) 2960 (C-H aromatic), 2874 (C-H aliphatic), 1708 (s, C=O acrylate), 1639 (C=C alkene), 1600 (C=C aromatic), 1509, 1466, 1415, 1384, 1312, 1279, 1257, 1230, 1196, 1158 (vs, C-O stretch) 1095, 1063, 1025, 980, 868, 829, 790, 738, 509, 414. Data matches the literature for this compound.[1]

Lab book ref. DRH-03-159 (for characterisation data)

2.2 Synthesis of Compounds

Synthesis of [Pd(P^C)(μ$_2$-OH)]$_2$ Palladacycle 2

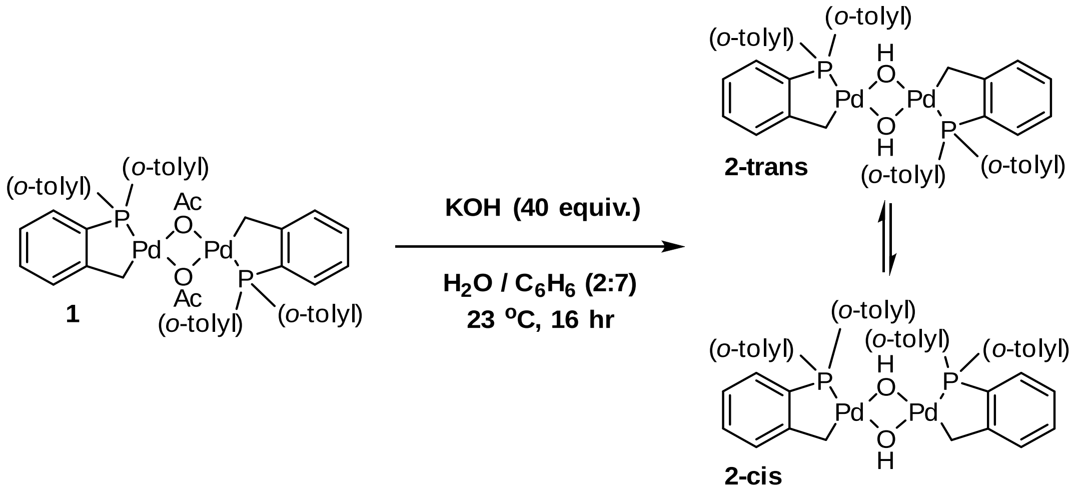

All solvents were degassed with N_2, and all reactions and manipulations were performed under a nitrogen atmosphere. The Herrmann-Beller palladacycle **1** (750 mg, 0.80 mmol, 1 eq.) was added to benzene (15 mL) and stirred to give a yellow suspension. To this, KOH (2290 mg, 40.8 mmol, 51 eq.) dissolved in deionised water (4 mL) was added, and the biphasic mixture stirred vigorously for 18 h. The cream suspension in the benzene layer was decanted, and the aqueous layer extracted with warm benzene (60 °C, 4 × 6 mL). The organic layers were collected, washed with water (2 × 10 mL) and the solvent removed *in vacuo*, giving a cream coloured product **2** as *cis*/*trans* isomers in a 1:2 ratio in solution (683 mg, 66%); ^{1}H NMR (500 MHz, DCM-*d*$_2$, 203 K) (Note: due to the presence of several isomers in solution at low temperatures, this is a complex NMR spectrum with many peaks. As such, individual environments have only been assigned as aromatic (Ar-H), aliphatic (CH_3, CH_2), or bridging OH (μ-OH). The reported nucleotides here have been scaled to give an overall count of 42H, which is the theoretical number of protons on the structure, and have been reported to the nearest 0.5 (0.1 for the μ-OH environments) δ; 7.46

– 7.33 (m, 3.5H, Ar-*H*), 7.33 – 7.22 (m, 9H, Ar-*H*), 7.10 (dd, *J* = 13.2, 7.8 Hz, 3H, Ar-*H*), 7.08 – 6.98 (m, 1.5H, Ar-*H*), 6.95 (d, *J* = 11.0 Hz, 1H, Ar-*H*), 6.81 – 6.70 (m, 2.5H, Ar-*H*), 6.71 – 6.61 (m, 1H, Ar-*H*), 3.07 (d, *J* = 14.2 Hz, 0.5H, CH_2), 2.99 (s, ~3H (overlapping with impurity), CH_3), 2.99 – 2.90 (m, 2.5H, CH_2), 2.85 (d, *J* = 6.8 Hz, 1.5H, CH_2), 2.82 (d, *J* = 3.3 Hz, 0.5H, CH_2), 2.79 (s, 1.5H, CH_3), 2.75 (dd, *J* = 14.0, 3.3 Hz, 0.5H, CH_2), 2.64 (d, *J* = 2.9 Hz, 2.5H, CH_3), 2.53 (s, 3.5H, CH_3), 2.36 (s, 1H, CH_3), 2.22 (s, 1.5H, CH_3), -0.65 (s, 0.2H, μ-OH *cis*), -0.93 (s, 0.1H, μ-OH *cis*), -1.26 (d, 1.5H, *J* = 2.7 Hz, μ-OH *trans*), -1.40 (d, 0.5H, *J* = 2.8 Hz, μ-OH *trans*), -2.31 (s, 0.2H, μ-OH *cis*), -2.44 (s, 0.1H, μ-OH *cis*); ^{13}C NMR (135 MHz, DCM-d_2, 298 K) δ; 158.9 (dd, *J* = 31.5, 8.7 Hz, Ar-*C*), 142.8 (Ar-*C*), 135.8 (d, *J* = 56.3 Hz, Ar-*C*), 132.7 (Ar-*C*), 132.6 (Ar-*C*), 132.1 (Ar-*C*), 132.0 (Ar-*C*), 131.2 (d, *J* = 2.2 Hz, Ar-*C*), 131.2 (d, *J* = 2.5 Hz, Ar-*C*), 131.0 (Ar-*C*), 130.7 (Ar-*C*), 128.3 (dd, *J* = 21.5, 8.1 Hz, Ar-*C*), 126.2 (Ar-*C*), 125.8 (Ar-*C*), 125.7 (Ar-*C*), 125.7 (Ar-*C*), 26.6 (CH_2), 24.9 (CH_2), 23.0 (CH_3), 22.9 (CH_3), 22.8 (CH_3); ^{31}P NMR (202.5 MHz, DCM-d_2, 203 K) δ; 34.02 (*trans* isomer), 33.56 (*cis* isomer), 33.37 (*cis* isomer), 33.18 (*trans* isomer); HRMS (ESI$^+$) $(C_{42}H_{43}O_2P_2Pd_2)^+$ *m/z* (calculated) 853.0802 (found) 853.0785, mass difference 1.7 ppm, $(C_{21}H_{22}OPPd)^+$ *m/z* (calculated) 427.0438, (found) 427.0453, mass difference 1.6 ppm. FTIR (ATIR): $\tilde{\nu}$ (cm^{-1}) 3646 (O-H), 3051 (C-H aromatic), 2857 (C-H aliphatic), 1582 (C=C aromatic), 1466, 1440 (C-P), 1283, 1069, 750; Far IR (ATIR) $\tilde{\nu}$ (cm^{-1}) 586, 560, 525, 509, 477, 459, 441, 417, 362, 316, 269, 249; mp. decomposed above 188 °C. Lab book ref. DRH-01-108.

Synthesis of the Palladacyclic $Pd_4(\mu_4\text{-}O)(\mu_2\text{-}OH)_2$ Cluster 3

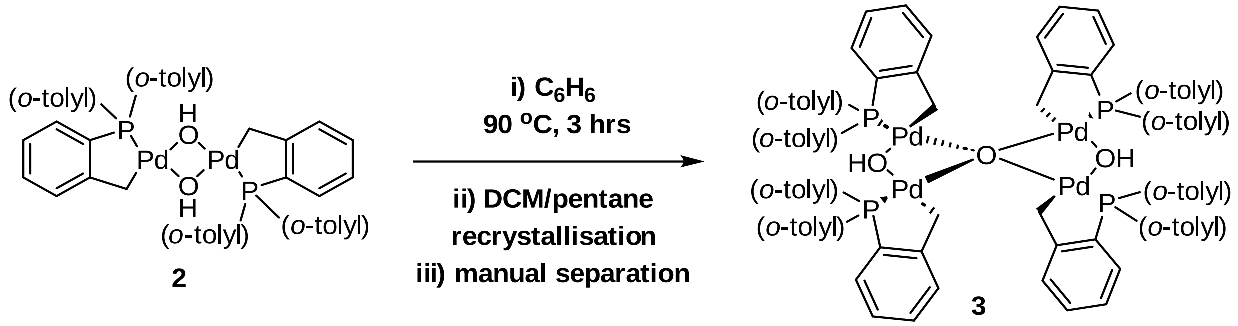

$[Pd(P^\wedge C)(\mu_2\text{-}OH)]_2$ palladacycle **2** (45 mg, 0.053 mmol) was added to still-dried degassed benzene (3 mL) under Schlenk conditions. The yellow solution was vigorously stirred and refluxed under a static N_2 atmosphere for 2.5 h at 90 °C. The solvent was rapidly removed under vacuum, giving a pale-yellow powder. This powder was transferred to an argon glove box, where it was dissolved in *ca.* 0.8 mL of dry, degassed DCM and placed in a 4 small vials. These vials were placed in larger vials containing *ca.* 1.5 mL pentane, and the system was sealed. After 1 week, a mixture of colourless crystals and vibrant yellow crystals of $Pd_4(\mu_4\text{-}O)(\mu_2\text{-}OH)_2$ cluster **3** suitable for X-ray diffraction were obtained. The yellow crystals from two of the vials were manually separated under a microscope, giving 2.4 mg of **3** (projected total yield = 4.8 mg, 0.0028 mmol, 5%). ^1H NMR (500 MHz, DCM-d_2, 203 K) δ; 7.59 – 7.47 (m, 3H, Ar-*H*), 7.44 – 7.33 (m, 2H, Ar-*H*), 7.33 – 7.24 (m, 9H, Ar-*H*), 7.23 – 7.02 (m, 4H, Ar-*H*), 7.01 – 6.93 (m, 4H, Ar-*H*), 6.79 (dt, *J* = 18.8, 8.8 Hz, 4H, Ar-*H*), 6.69 (q, *J* = 9.3 Hz, 1H, Ar-*H*), 6.61 (d, *J* = 7.9 Hz, 1H, Ar-*H*), 6.56 – 6.48 (m, 1H,

Ar-*H*), 6.41 (t, *J* = 8.6 Hz, 1H, Ar-*H*), 6.26 (d, *J* = 7.7 Hz, 1H, Ar-*H*), 6.22 (d, *J* = 7.9 Hz, 0.5H, Ar-*H*), 5.56 (d, *J* = 7.5 Hz, 1H), 4.54 (d, *J* = 14.6 Hz, 0.5H, CH_2), 4.48 (d, *J* = 14.0 Hz, 1H, CH_2), 4.42 (d, *J* = 13.6 Hz, 1H, CH_2), 4.36 (d, *J* = 13.8 Hz, 0.5H, CH_2), 4.26 (d, *J* = 13.6 Hz, 1H, CH_2), 3.98 (d, *J* = 14.0 Hz, 1H, CH_2), 3.71 (d, *J* = 14.5 Hz, 1H, CH_2), 3.51 – 3.38 (m, 1H, CH_2), 3.21 (d, *J* = 12.7 Hz, 1H, CH_2), 3.10 – 2.93 (m, 5H, CH_3), 2.88 (s, 6H, CH_3), 2.76 (d, *J* = 7.3 Hz, 4H, CH_3), 2.73 (s, 4H, CH_3), 2.71 – 2.63 (m, 4H, CH_3), 2.60 (s, 1H, CH_3), 2.56 (s, 4H, CH_3), 2.36 (s, 2H, CH_3), 2.25 (s, 1H, CH_3), -3.46 (s, μ-OH, 0.5H), -3.51 (s, μ-OH, 1H), -3.68 (s, μ-OH, 0.5H) (Note: Due to the complexity of the spectra, it is impossible to differentiate between peaks belonging to different isomers of **3** at low temperature. These isomer peaks have been denoted with 0.5H, indicating they are not fully populated proton environments. Additionally, because of the presence of a small amount of **2** forming in solution, only peaks relevant to the Pd_4 cluster **3** have been selected for ^{31}P NMR); ^{31}P NMR (203 MHz, DCM-d_2, 203 K) δ; 32.09, 30.08, 29.99, 29.38, 29.04, 28.85, 28.61; HRMS (LIFDI) $(C_{84}H_{82}O_3P_4Pd_4)^{+\bullet}$ *m/z* (calculated) 1686.13483, (found) 1686.14148, mass difference 3.95 ppm; (ATIR): $\tilde{v}$ (cm^{-1}) 3626 (O-H), 3050 (C-H aromatic), 2858 (C-H aromatic), 1582 (C=C aromatic), 1465, 1448 (C-P), 1282, 1071, 752, 440; Far IR (ATIR) $\tilde{v}$ (cm^{-1}) 583, 559, 523, 494, 468, 451 (Pd-O), 401, 374, 270, 248.

Lab book ref. DRH-01-131 (X-ray diffraction structure ijsf21009), DRH-01-146, DRH-01-152

Special Details for the Characterization and Catalytic use of 3

Due to difficulties in synthesizing and purifying $Pd_4(\mu_4\text{-}O)(\mu_2\text{-}OH)_2$ cluster **3**, the complex was isolated by recrystallizing a mixture of colourless $[Pd(P^\wedge C)(\mu_2\text{-}OH)]_2$ palladacycle **2** and yellow **3** by the method described above. The yellow crystals were manually separated and isolated using needles and scalpels in oil, yielding approximately 2 mg of **3** (2.4 mg by mass, but the presence of oil made accurate weighing impossible). To verify the identity of the yellow crystals, the unit cell parameters of one single crystal was checked against the known unit cell parameters of 3, giving a 100% match (Lab book ref. of crystal structure DRH-01-131). Microscope images of the yellow crystals during crystal picking are shown below. These crystals were characterised by ATIR, dissolved in DCM-d_2 (0.5 mL, dried over CaH_2, freeze-pump-thaw degassed) in an Ar glove box, cooled to -78 °C and characterised by NMR spectroscopy at 203 K. For catalytic use, approximately 1 mg of **3** was dissolved in DCM-d_2 and transferred to the reaction vessel before any other solvents or reagents were charged, and the solvent removed *in vacuo*. The reagents were then charged in accordance with General Procedure 2.1.

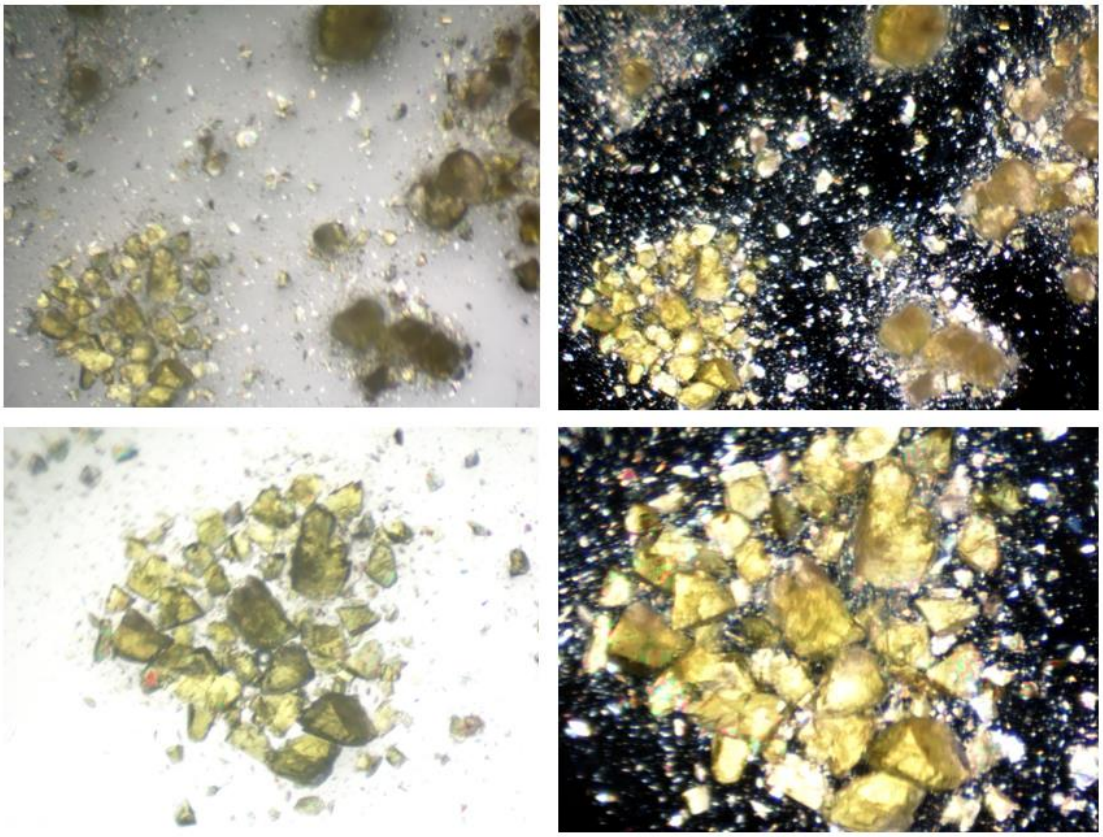

Figure 1: Vivid yellow crystals of the $Pd_4(\mu_4\text{-}O)(\mu_2\text{-}OH)_2$ cluster 41 being manually isolated. Top Left: a mixture of cluster and impure crystals (cloudy/brown). Top Right: same image under plane polarised light. Bottom Left: magnified image of the pure cluster crystals. Bottom Right: same image under plane polarised light.

Synthesis of the $[Pd(P^\wedge C)(\mu_2\text{-}Cl)]_2$ Palladacycle 4

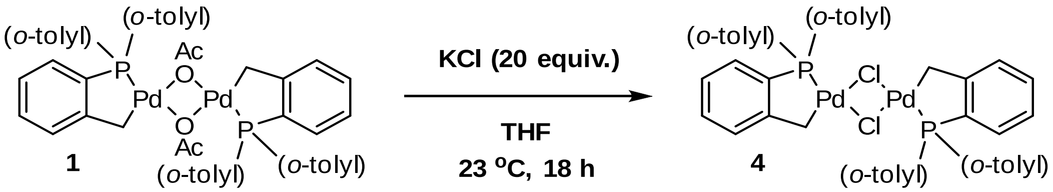

The Herrmann-Beller palladacycle **1** (150 mg, 0.16 mmol, 1 eq.) and anhydrous KCl (239 mg, 3.2 mmol, 20 eq.) were added to THF (10 mL, dry, degassed) under N_2. The resulting mixture was rapidly stirred for 18 hours and stirred to give a yellow suspension. Deionised water (10 mL) then DCM (40 mL) were added (Note: This complex is sparingly soluble in DCM, so larger volumes may be needed). The organic layers were collected, washed (water, 2 × 20 mL), and the solvent evaporated (without drying) in vacuo to give the $[Pd(P^\wedge C)(\mu_2\text{-}Cl)]_2$ palladacycle **4** as a bright yellow powder (134 mg, 94%); ^1H NMR (500 MHz, DCM-d_2, 203 K) δ 7.47 – 7.18 (m, 12H, Ar-*H*), 7.18 – 6.94 (m, 8H, Ar-*H*), 6.86 – 6.65 (m, 4H, Ar-*H*), 3.59 – 3.42 (m, 2H, C*H*H), 3.37 (q, *J* = 13.8, 11.3 Hz, 2H, CH*H*), 2.79 (s, 2H, tolyl-*H*), 2.73 (s, 1H, tolyl-*H*), 2.66 (s, 1H,

tolyl-*H*), 2.65 (s, 3H, tolyl-*H*), 2.54 (s, 2H, tolyl-*H*), 2.44 (s, 1H, *tolyl-H*), 2.40 (s, 1H, tolyl-*H*), 1.70 (s, 1H, tolyl-*H*); ^{31}P NMR (202 MHz, DCM-d_2, 203 K) δ 39.06 (s), 38.86 (s), 38.76 (s), 38.64 (s). (Note: due to extremely low solubility in tested solvents, it was not possible to get a ^{13}C NMR of this complex. At 203 K, the broad ^{31}P environment observed at room temperature splits into 4 peaks, each representing a structural isomer of this complex); HRMS (LIFDI) $(C_{42}H_{40}P_2Pd_2Cl_2)^{+\bullet}$ m/z (calculated) 888.00465, (found) 888.00508, mass difference 0.49 ppm; (ATIR): ṽ (cm-1) 3053 (C-H aromatic), 2969 (C-H aromatic), 2940 (C-H aliphatic), 2869 (C-H aliphatic), 1586 (C=C aromatic), 1578 (C=C aromatic), 1464, 1440 (C-P), 1283, 1264, 1203, 1162, 1130, 1083, 1052, 805, 757, 714, 579, 559, 518, 479, 469, 461; mp. decomposed above 280 °C.

Lab book ref. DRH-02-130, DRH-03-78

Synthesis of Ag-C_6F_5 Complex

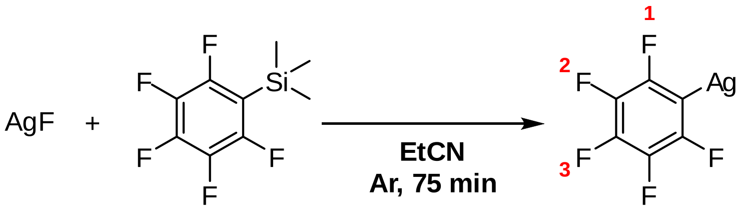

Complex prepared according to a literature procedure by Athavan *et al.*[2]

In an argon glovebox, AgF (302 mg, 2.38 mmol, 1.0 eq.) and dry, degassed EtCN (5 mL) were stirred for 5 min at room temperature under darkness. $Me_3SiC_6F_5$ (450 µL, 2.38 mmol, 1.02 eq.) was added, and the resulting mixture was stirred for a further 75 min. The reaction was removed from the glovebox, and volatiles removed *in vacuo*, giving the product as a pale grey powder (442 mg, 68%). (Note: this complex is unstable in air and sensitive to light and temperature, so was stored in an argon glovebox freezer in a foiled vial) ^{19}F NMR (471 MHz, Acetonitrile-d_3) δ -106.93 (m, 2F, F-1), -159.80 (t, *J* = 19.2 Hz, 1F, F-3), -163.13 (m, 2F, F-2). The ^{19}F NMR data matches the literature.[2]

Lab book ref. DRH-03-73

Isolation of Palladacyclic Pd_4 Cluster 5

Lab book ref. DRH-03-75

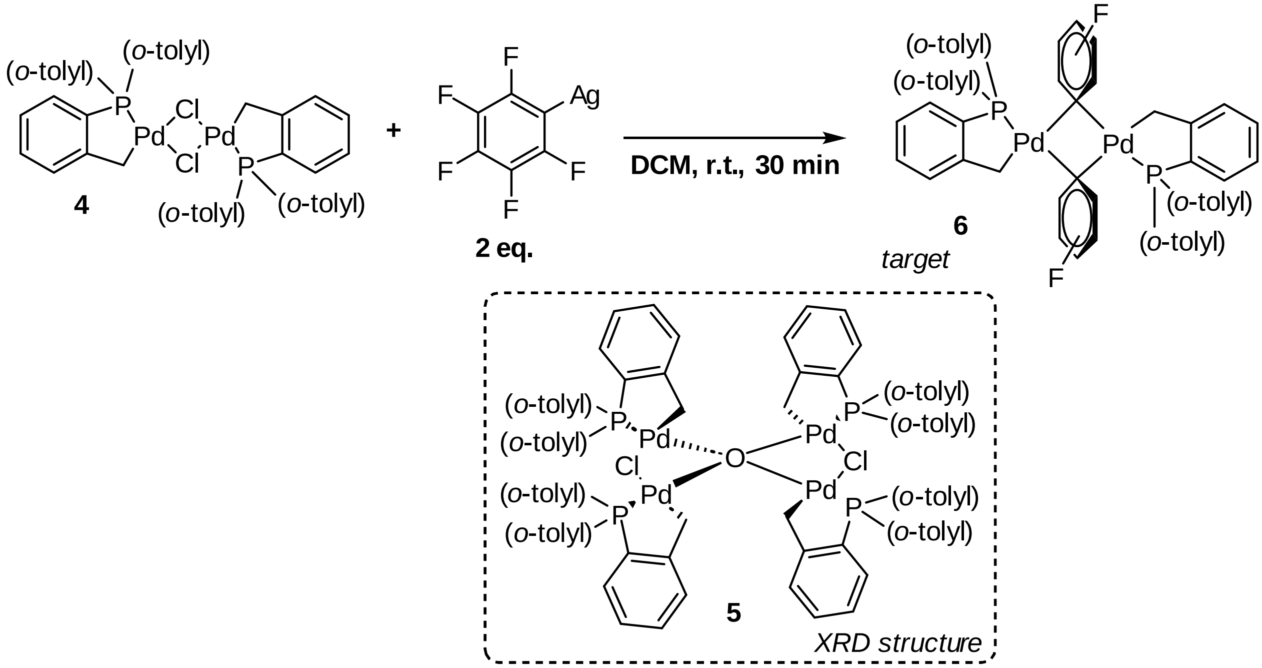

In an argon glovebox under darkness, [Pd(P^C)(μ_2-Cl)]$_2$ palladacycle **4** (20 mg, 0.0225 mmol, 1.0 equiv.), AgC_6F_5 (15 mg, 0.0450 mmol, 2.0 equiv.) and dry, degassed DCM (5 mL) were charged in a Schlenk tube and rapidly stirred for 30 min. After this time, 0.5 mL of the dark orange reaction supernatant was removed to a J-young tab NMR tube and analysed by NMR spectroscopy, showing a mixture of species. Of particular interest is the broad peak from ^{31}P δ 40 – 38 ppm, which is symbolic of a bridging cluster complex. This sample was returned to the Ar glovebox and crystallised using slow vapor diffusion in a sealed vial (DCM/pentane, 0.25 mL : 1.5 mL). After several days, a number of bright yellow crystals of **5** suitable for XRD were obtained (~ 2 mg), along with amorphous orange solid that was likely the result of decomposition.

The rest of the sample was removed from the Ar glovebox, the supernatant was decanted, and solvent removed *in* vacuo, giving a red solid with mass 14.4 mg, but LIFDI mass spectrometric analysis showed no presence of **5** or the target product **6**.

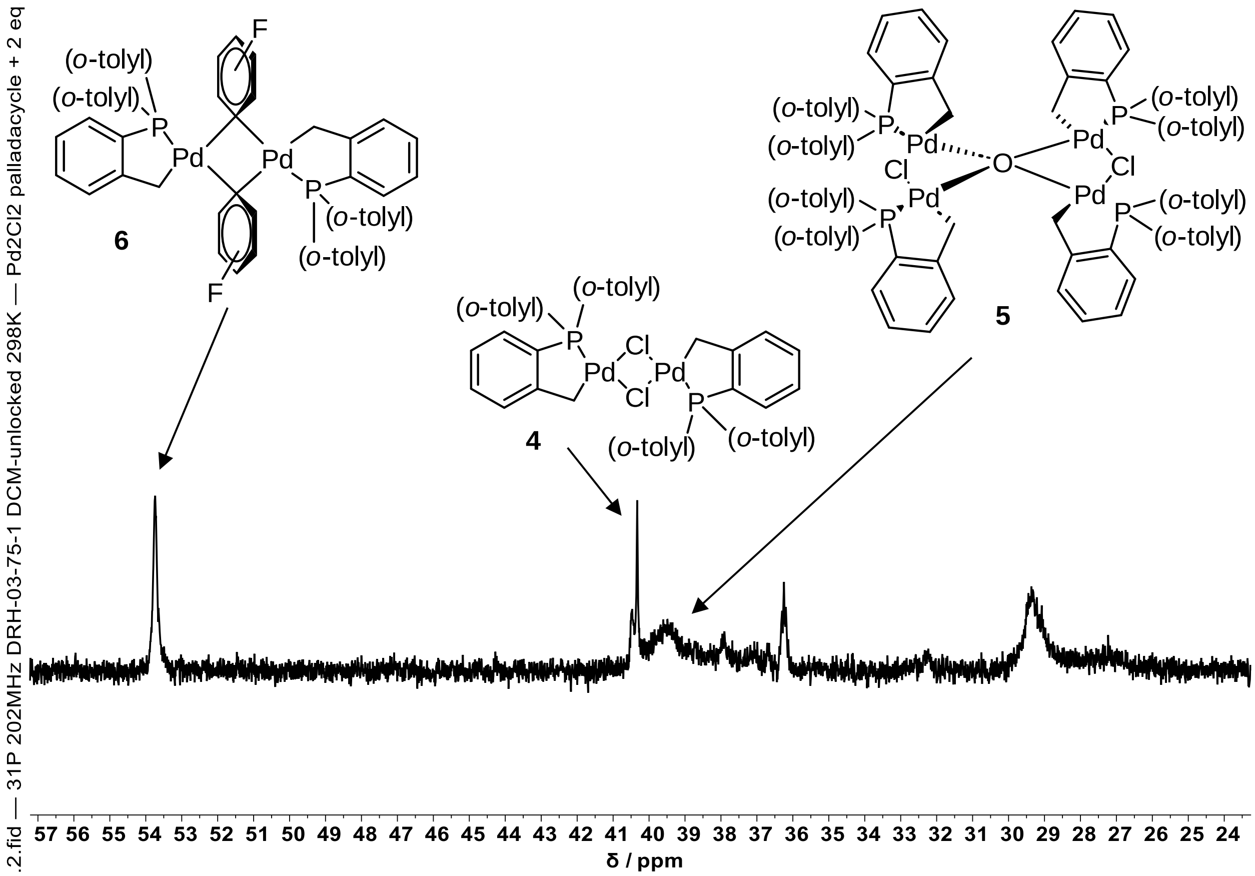

Figure 2: ^{31}P NMR (203 MHz, DCM-unlocked, 128 sc, 298 K) of the reaction mixture showing a potential peak for Pd_4 cluster 5 and the proposed identities of some other relevant peaks. Lab book ref. DRH-03-75-1

Synthesis of $[Pd(P^\wedge C)(\mu_2\text{-}Ar^F)]_2$ Palladacycle 6

Lab book ref. DRH-03-76, DRH-03-79

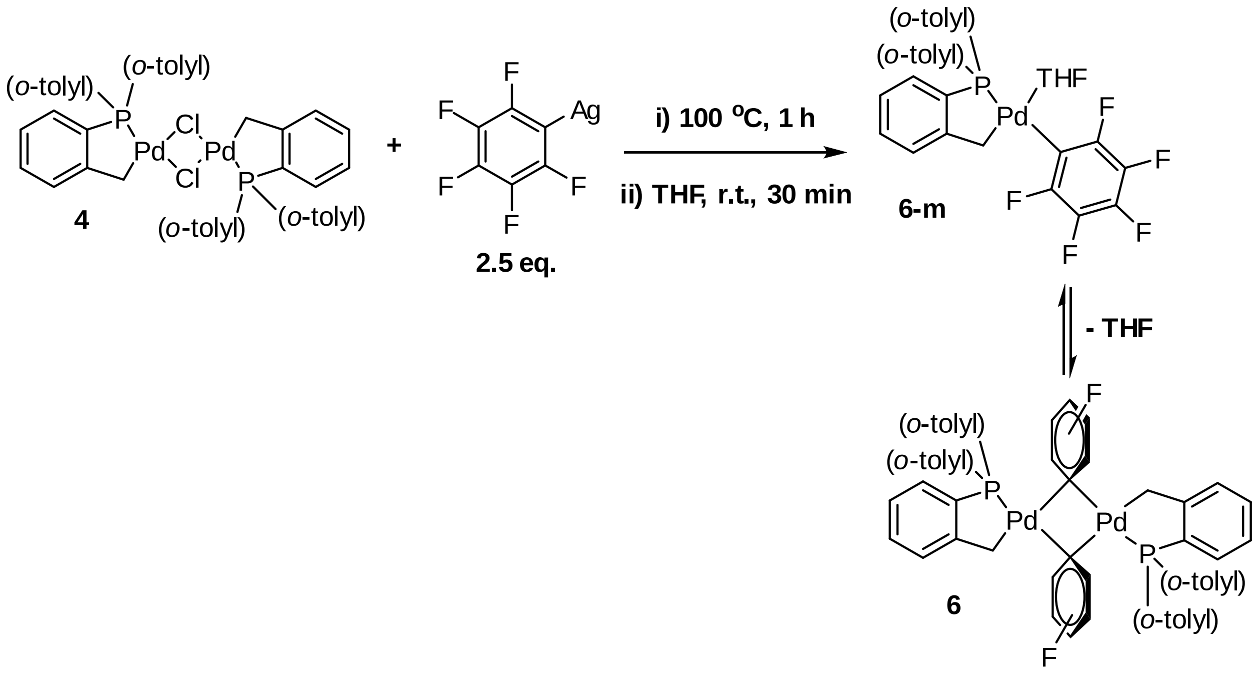

The synthesis of this complex was based on that from Espinet's work, where bridging Pd-Ar-Pd complexes were synthesised and characterised.[3] The complex is highly water sensitive, rapidly decomposing to Pd black and pentafluorobenzene when exposed to water.

In an argon glovebox, Ag-C_6F_5 complex (47 mg, 0.141 mmol, 2.1 equiv.) was charged into a Schlenk flask under darkness. The complex was heated at 100 °C under vacuum for 1 h to remove any coordinating EtCN ligands, then allowed to cool to room temperature. $[Pd(P^\wedge C)(\mu_2\text{-}Cl)]_2$ palladacycle **4** (60 mg, 0.0674 mmol) was added under N_2, followed by THF (6 mL, dry, degassed). The colourless mixture was stirred for 30 min at room temperature, after which a direct NMR aliquot was taken for analysis. The mixture was allowed to settle, and the liquor isolated by canula filtration. The solvent was removed *in vacuo*, giving a dark red residue. This residue was analysed by NMR (C_6D_6, dry, degassed), and ~10 mg was set up for recrystallization overnight (C_6D_6/pentane) under argon (slow vapor diffusion). A small quantity of orange-yellow crystals of **6** suitable for single crystal XRD were obtained, and these were analysed by FTIR and mass spectrometry.

The bridging Pd-Ar bond is weak and easily cleaved by even a mildly coordinating solvent. As such, in THF, there is exclusively the monomeric complex **6-m**, likely coordinated with THF. As the coordinating solvent is removed, the more stable dimer **6** is formed, and this is what crystallises out. Analysis in dry Benzene-d_6 reveals that even in non-coordinating solvent, the monomer forms. Over longer time periods (ca. 2 days)

degradation of the complex is observed, which has made larger scale synthesis and purification impossible. LIFDI mass spectrometry analysis shows only the monomer present, as the dimer **6** likely cleaves under mass spectrometry conditions.

Mixture of monomer and dimer in Benzene-d_6; ^{31}P NMR (243 MHz, Benzene-d_6, 298 K) δ 53.17 (dimer), 26.09 (monomer); ^{19}F NMR (565 MHz, Benzene-d_6, 298 K) δ -95.92 (dimer), -113.77 (d, J = 35.5 Hz, monomer), -145.28 (dimer), -160.79 – -161.08 (m, dimer/monomer), -162.34 – -162.55 (m, dimer/monomer).

6-m in THF: ^{31}P NMR (203 MHz, THF-unlocked, 298 K) δ 25.60 (tt, J = 12.2, 9.2 Hz); ^{19}F NMR (471 MHz, THF-unlocked, 298 K) δ -114.77 (dt, J = 34.2, 9.6 Hz), -163.80 (d, J = 21.5 Hz), -164.54.

HRMS (LIFDI) $(C_{27}H_{20}F_5PPd)^{+\bullet}$ *m/z* (calculated) 576.02521, (found) 576.02786, mass difference 4.59 ppm. (ATIR): $\tilde{\nu}$ (cm^{-1}) 3060 (C-H aromatic), 2925 (C-H aliphatic), 2857 (C-H aliphatic), 1624 (C=C aromatic), 1590 (C=C aromatic), 1580, 1492 (C-P), 1441 (s, C-F), 1320, 1282, 1263, 1246, 1203, 1132, 1048, 951, 803, 752, 713, 671, 577, 559, 521, 507, 460.

Lab book ref. DRH-03-76, DRH-03-79 (X-ray diffraction structure ijsf22048)

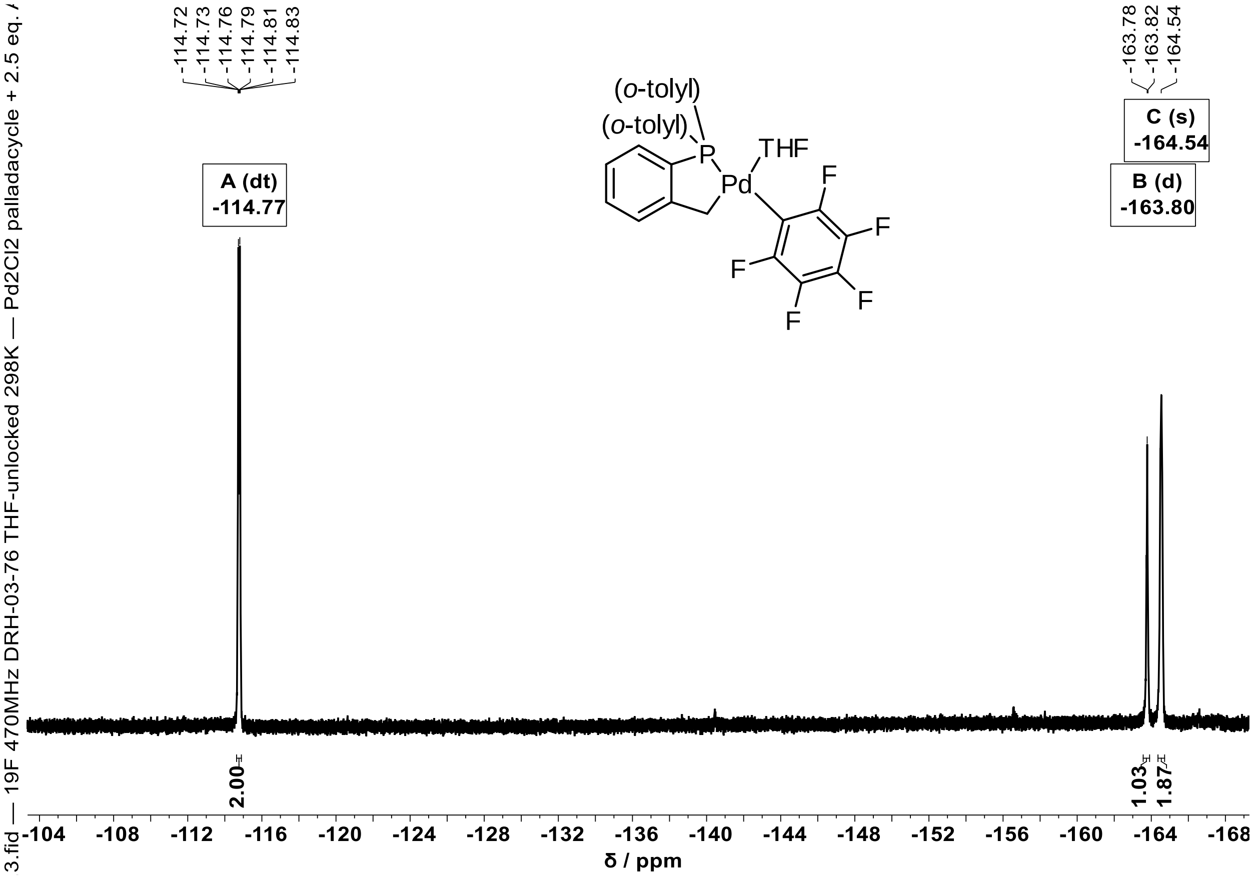

Figure 3: ^{19}F NMR (470 MHz, THF-unlocked, 128 sc, 298 K) of the [Pd(P^C)(Ar^F)] palladacyclic monomer (6-m), with suspected THF coordination. Lab book ref. DRH-03-76

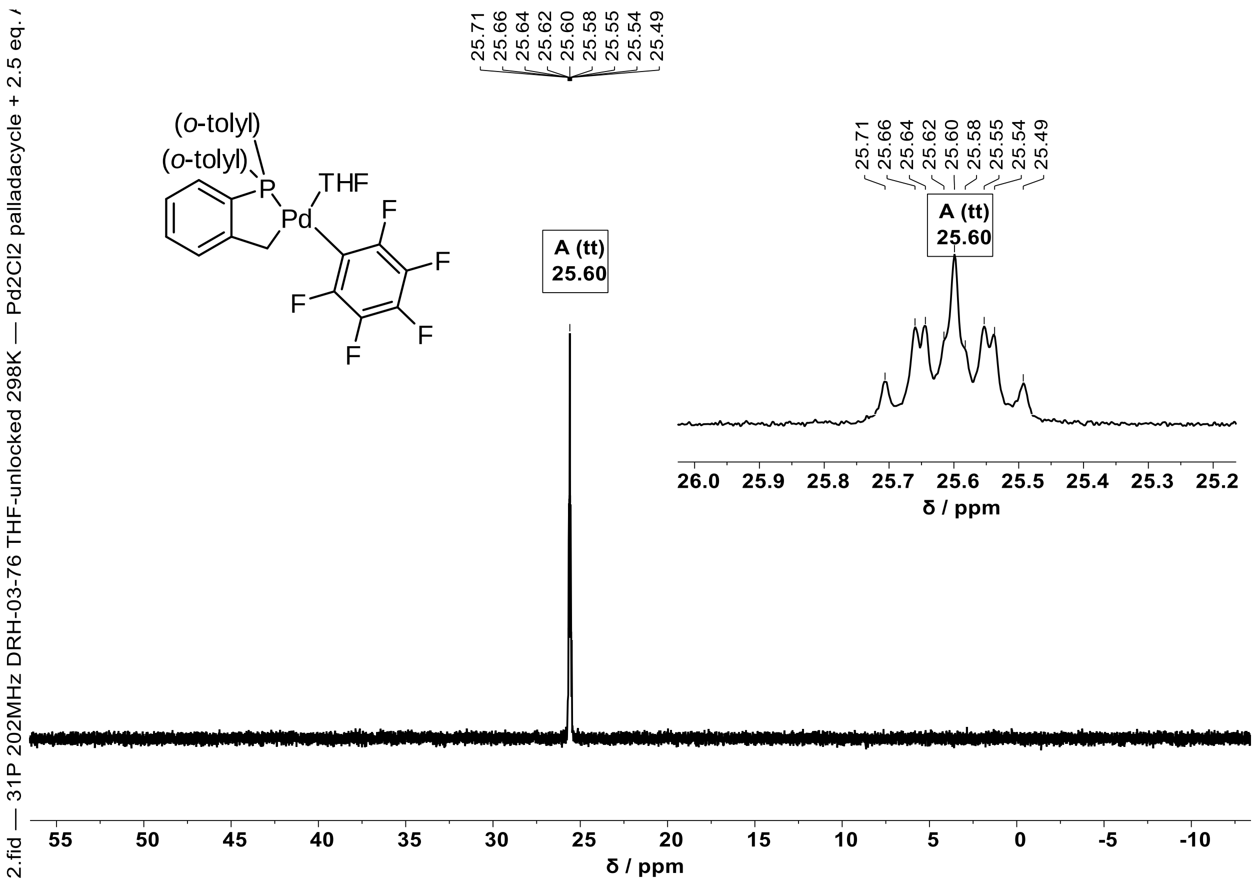

Figure 4: ^{31}**P NMR (203 MHz, THF-unlocked, 128 sc, 298 K) of the [Pd(P^C)(Ar^{F})] palladacyclic monomer 6-m with suspected THF coordination. Lab book ref. DRH-03-76**

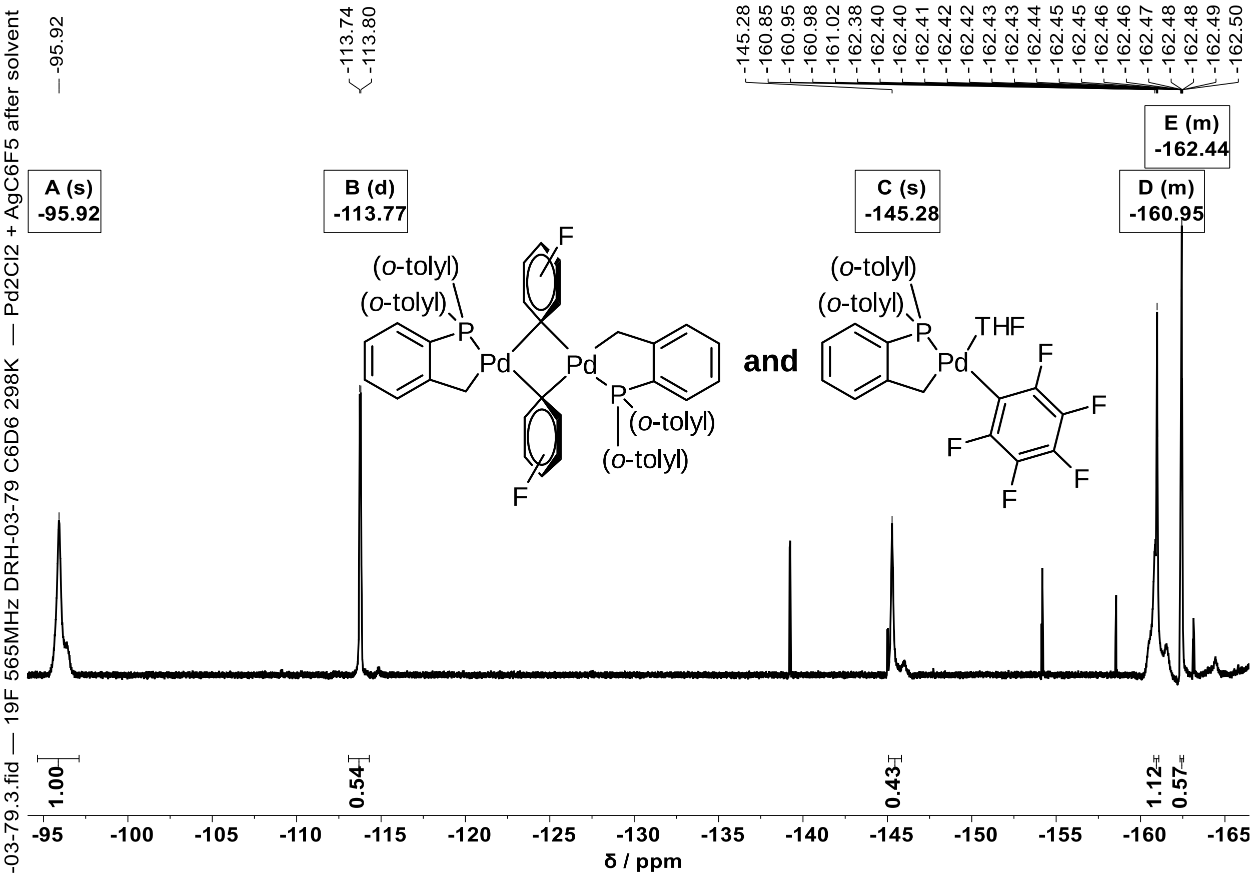

Figure 5: **^{19}F NMR (565 MHz, C_6D_6, 128 sc, 298 K) of the [Pd(P^C)(μ$_2$-Ar^F)]$_2$ palladacycle 6 and the [Pd(P^C)(Ar^F)] palladacyclic monomer 6-m in solution. Lab book ref. DRH-03-79**

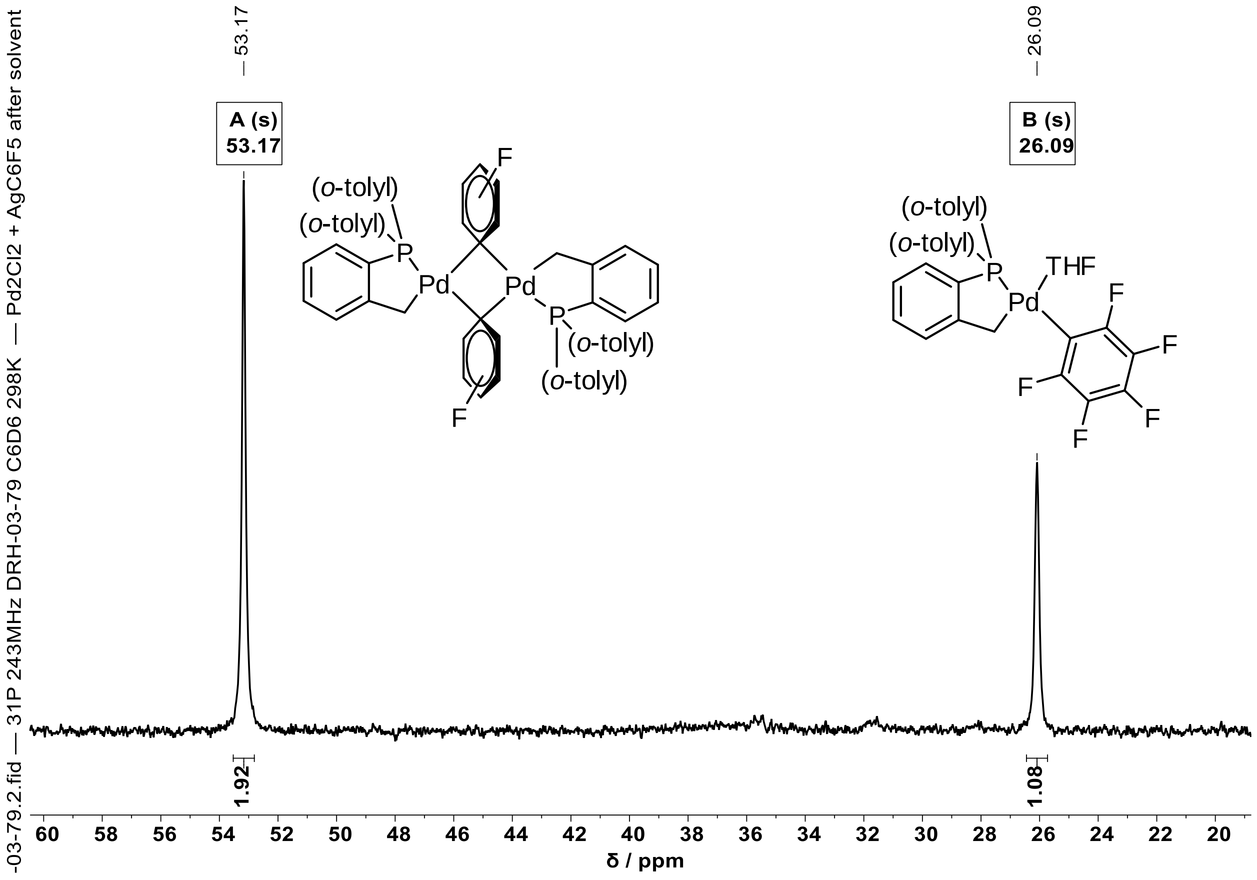

Figure 6: **^{31}P NMR (243 MHz, C_6D_6, 128 sc, 298 K) of the [Pd(P^C)(μ_2-Ar^F)]$_2$ palladacycle 6 and the [Pd(P^C)(Ar^F)] palladacyclic monomer 6-m in solution. Lab book ref. DRH-03-79**

3. Isolation and Confirmation of Structure of Palladacyclic Pd$_6$ cluster 7

Lab book ref. DRH-01-123

[Pd(P^C)(μ_2-OH)]$_2$ palladacycle **2** (approx. 30 mg) was added to still-dried (Na) degassed benzene (1mL per 10 mg) under Schlenk conditions. The yellow solution was vigorously stirred and refluxed under a static N_2 atmosphere for 21 h at 90 °C. The yellow-brown solution was filtered under N_2 to remove Pd black, then the solvent removed *in vacuo*. The solubility of the residue (for the purposes of recrystallisation) was tested by the addition then removal *in vacuo* of 1 mL of the following solvents: dichloromethane, toluene, hexane, pentane, diethyl ether. As the residue was soluble in all these solvents, water was then tested. To the residue, 1.5 mL of degassed water was added, then dry, degassed THF (ca. 1.5 mL) until the solid dissolved. A vent needle was added, and the sample placed in a fridge at 5 °C. After 4 weeks, a small quantity of orange-yellow crystals suitable for X-ray diffraction had formed (< 1 mg). This complex was primarily characterised by single crystal X-ray diffraction and revealed an unexpected complex containing 6 Pd centres arranged in two linked μ_3-OR (R = H, P) trigonal bipyramid structures. The two structures are

linked via an O-P-O bridge containing two methyl groups. Due to crystal twinning, there is uncertainty as to the identity of these methyl groups, but the P-C bond length of 1.878(9) Å and DFT modelling of alternative groups confirms that this is the most likely identity. This result is the most surprising aspect of the complex, as no obvious source of Me was used – indeed, the only available source is the o-tolyl groups. During crystallization or preparation, there was no DSMO used to supply a linker that would be indistinguishable by XRD. DFT was used to aid identification of this linker (see Section 6 for more details).

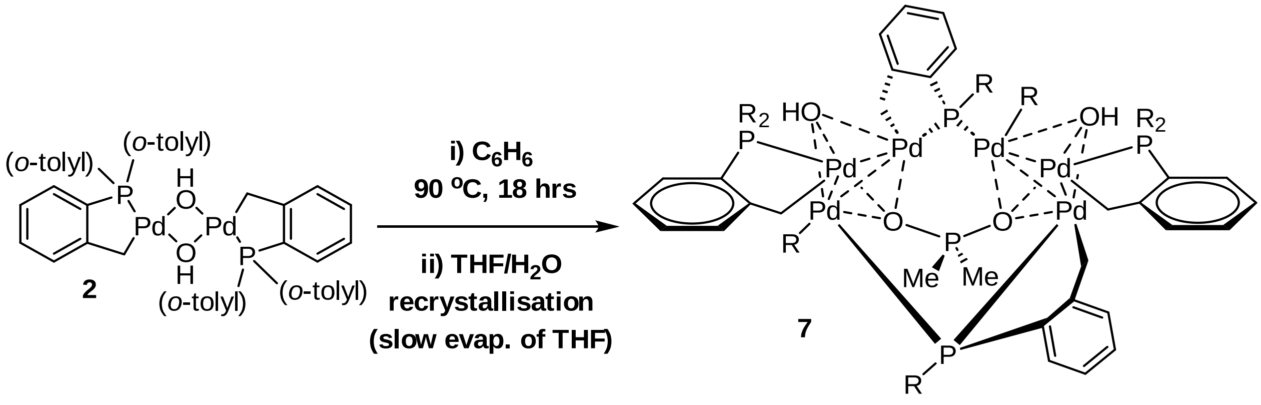

R = *o*-tolyl

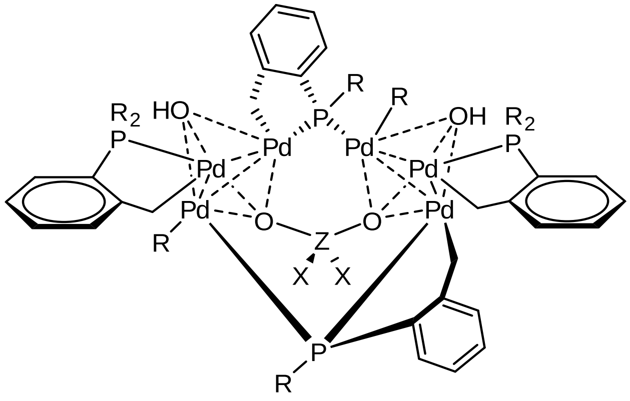

R = *o*-tolyl

Table 1: Relevant bond lengths and angles for computed structures of the Pd_6 cluster 7. Calculations performed at the b3lyp/def2svp level of theory, with Grimme's 3rd dispersion correction with Becke-Johnson damping. Solvent model (SVD, THF), and a superfine integration grid.

	Bond lengths / Å		Bond angles / °	
**Complex**	**O-Z**	**Z-X**	**O-Z-O**	**X-Z-X**
Crystal structure	***1.631(6)***	***1.878(9)***	***107.9(3)***	***109.7(4)***
Z = P, X = Me (**7a**)	1.58073	1.79819	109.44704	107.53866
Z = S, X = Me (**7b**)	1.86013	1.82205	159.87070	95.60422
Z = P, X = O (**7c**)	1.65532	1.50942	97.62349	121.71443
Z = P, X = OH (**7d**)	1.55819	1.59359	110.95213	99.43078

Due to the highly unusual structure and uncertainty in the fitting, DFT was used to model the Pd_6 cluster **7**. Above are shown selected bond lengths and angles for the atoms of interest. Although there is a significant discrepancy between the crystal structure parameters and those modelled computationally, the proposed identity of the atoms of P and Me seem to give the best agreement with the experimental data.

The other linking bridge is via a phosphido ligand, where one of the o-tolyl groups forms a palladacycle. It is assumed that the other *o*-tolyl group has migrated from the P ligand to the neighboring Pd center. The formal charge on each Pd center is + 5/3, indicating that some reduction compared to the dimer (2) has occurred. There has also been the loss of one phosphine from the structure compared to the dimer. One remarkable thing about this structure is that the palladacycles are retained, even after heating at 90 °C for 21 hours and after 4 weeks of recrystallisation under atmospheric conditions. This is a testament to the robustness of the Pd-C bond and raises questions about the activation of palladacycles under reaction conditions. Unfortunately, not enough material was obtained to fully characterise the complex. ESI mass spectrometry and LIFDI mass spectrometry yielded inconclusive results, but more success was had with NMR and FTIR of the obtained single crystals.

As a brief aside, it has proved challenging to repeat the crystallization of this complex, with several experiments yielding an amorphous glass rather than single crystals.

Calculated IR spectra were also obtained by the same DFT method and used to compare the experimental FTIR data collected.

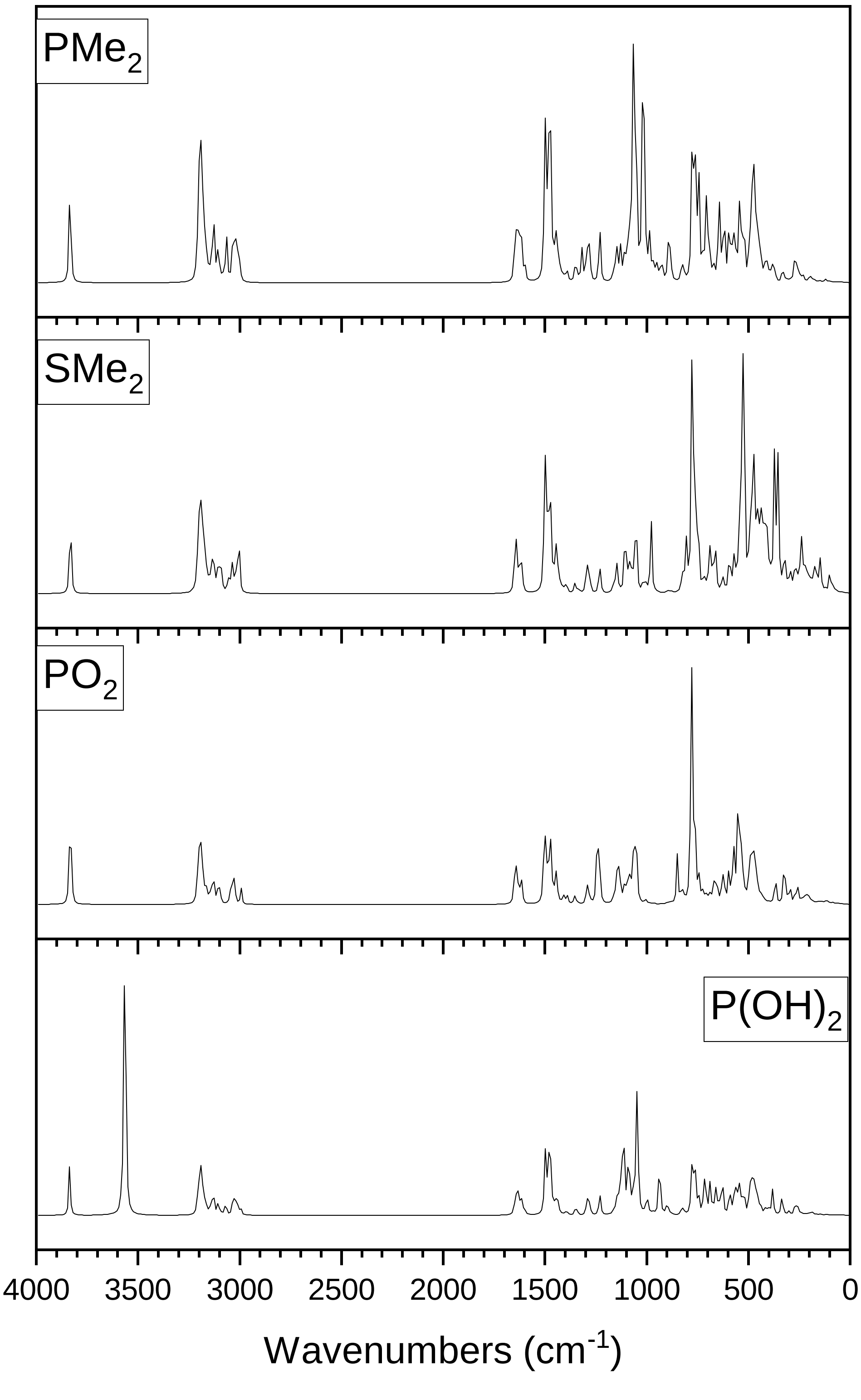

Figure 7: Stacked simulated IR spectra from DFT modelling of the Pd_6 cluster 7, where the linking group = PMe_2, SMe_2, PO_2, $P(OH)_2$.

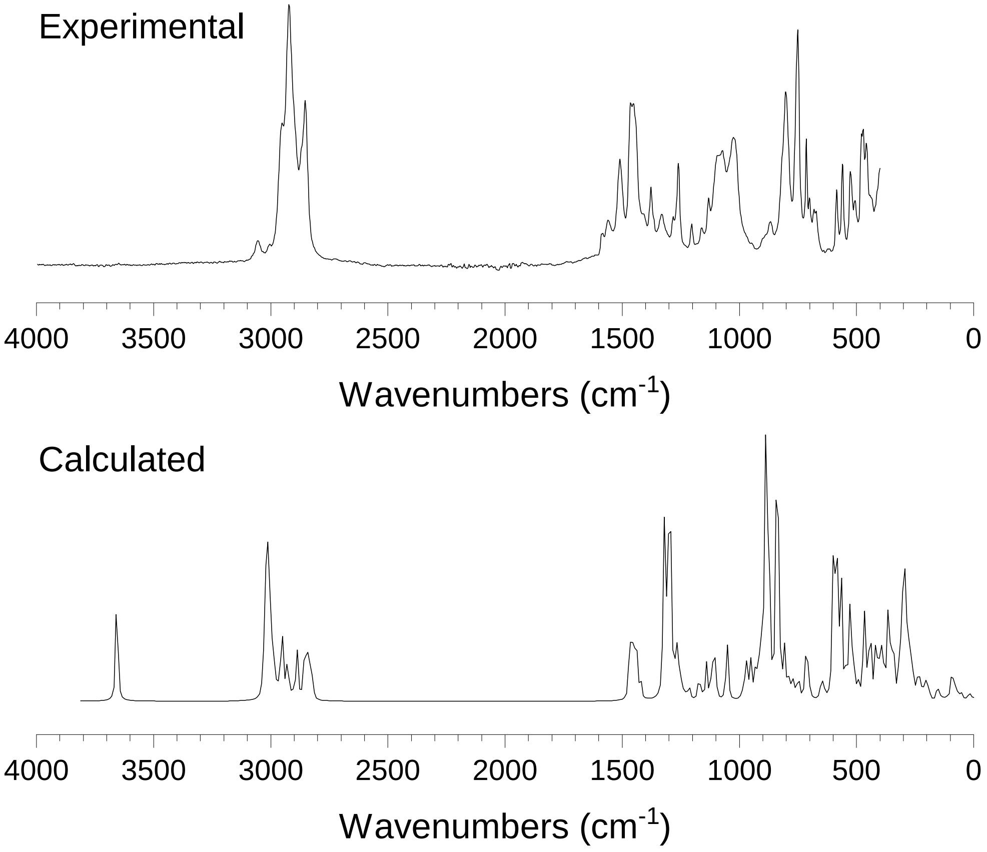

Figure 8: Stacked simulated IR spectra from DFT modelling of the Pd_6 cluster 7; top-experimental, bottom, calculated IR spectrum. The calculated IR spectrum was calibrated by matching the weak O-H stretch at 3660 cm^{-1} in the experimental spectrum.

P-C IR stretches (deformation) are characteristically strong in 1400-1450 cm^{-1} region, which are seen here. P-O stretches are also strong in the 900-1100 region in IR. Difficult to ascribe, but possible matches are highlighted in the spectra above. There are no P-OH bonds (weak at 1740-1600 cm^{-1}) present, indicating that this structure is unfeasible.

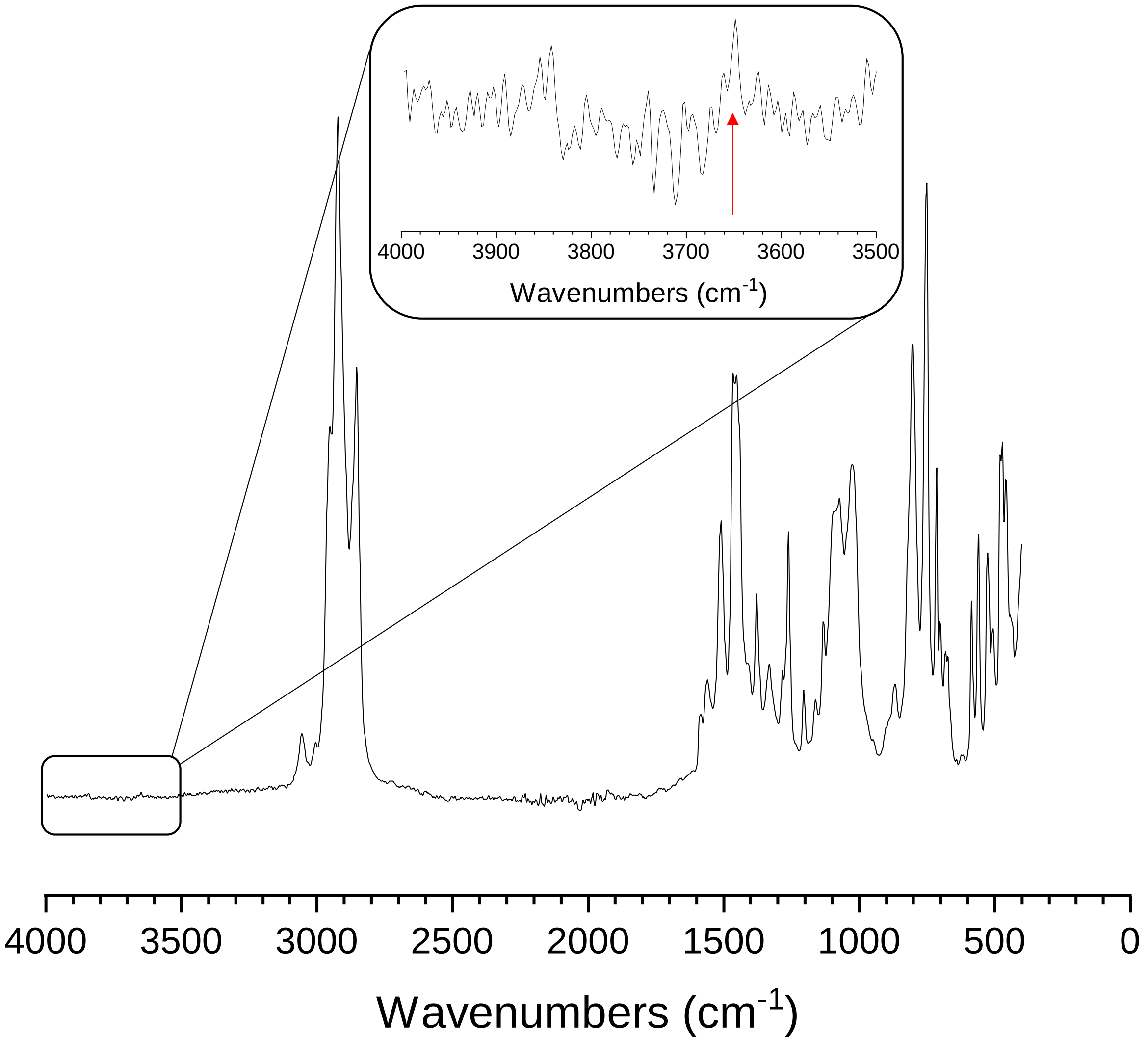

Figure 9: FTIR spectrum of the Pd_6 cluster 7. There remains some grease from the XRD mounting of the single crystals, resulting in an amplified C-H stretch peak at 2900 cm^{-1}. The very weak O-H stretch is magnified here.

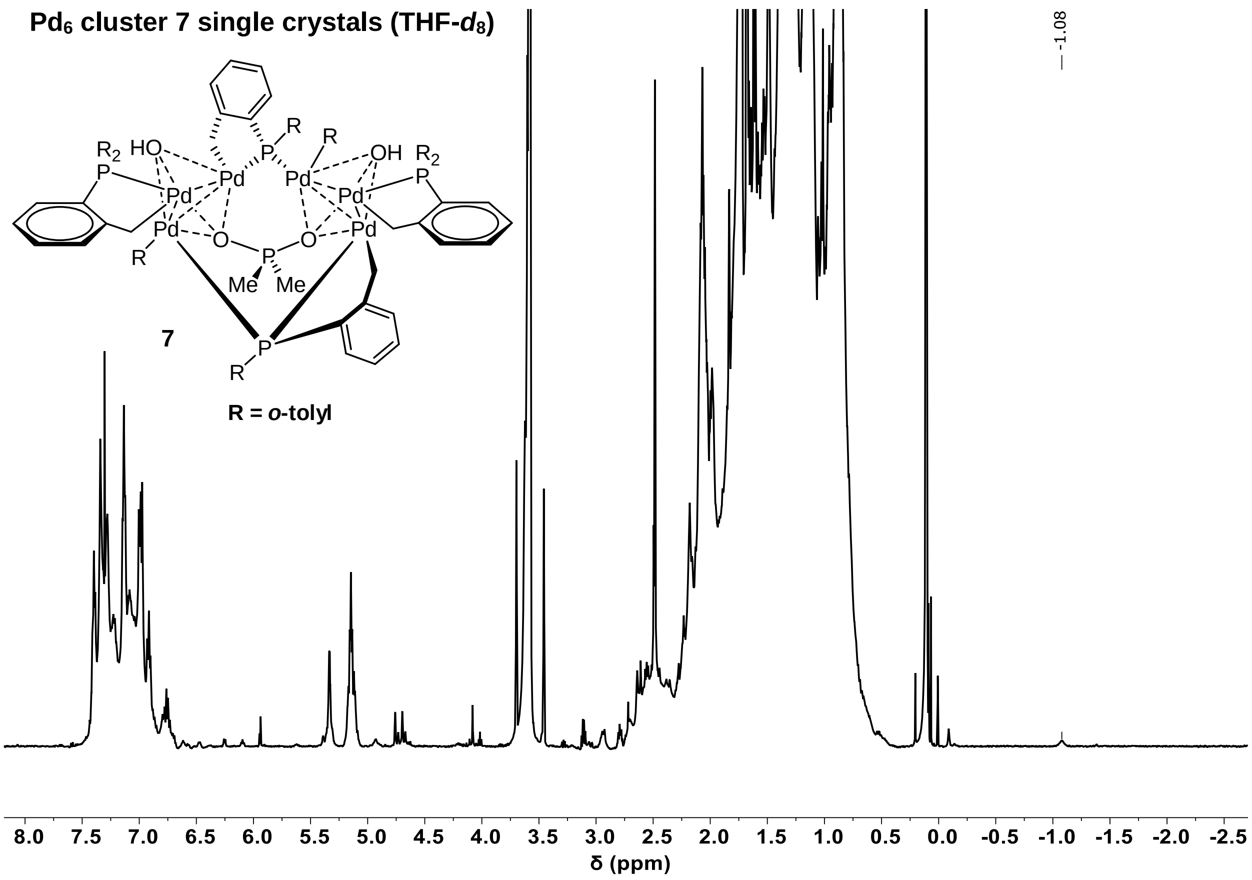

Figure 10: ^{1}H NMR (600 MHz, THF-d_8, 4096 scans, 298 K) spectrum of the Pd$_6$ cluster 7. Due to the sample being made from individually separated single crystals, there are significant grease peaks from the XRD mounting oil. The relevant Pd$_3$(μ_3-OH) proton signal is shown at -1.08 ppm. Lab book ref. DRH-01-123

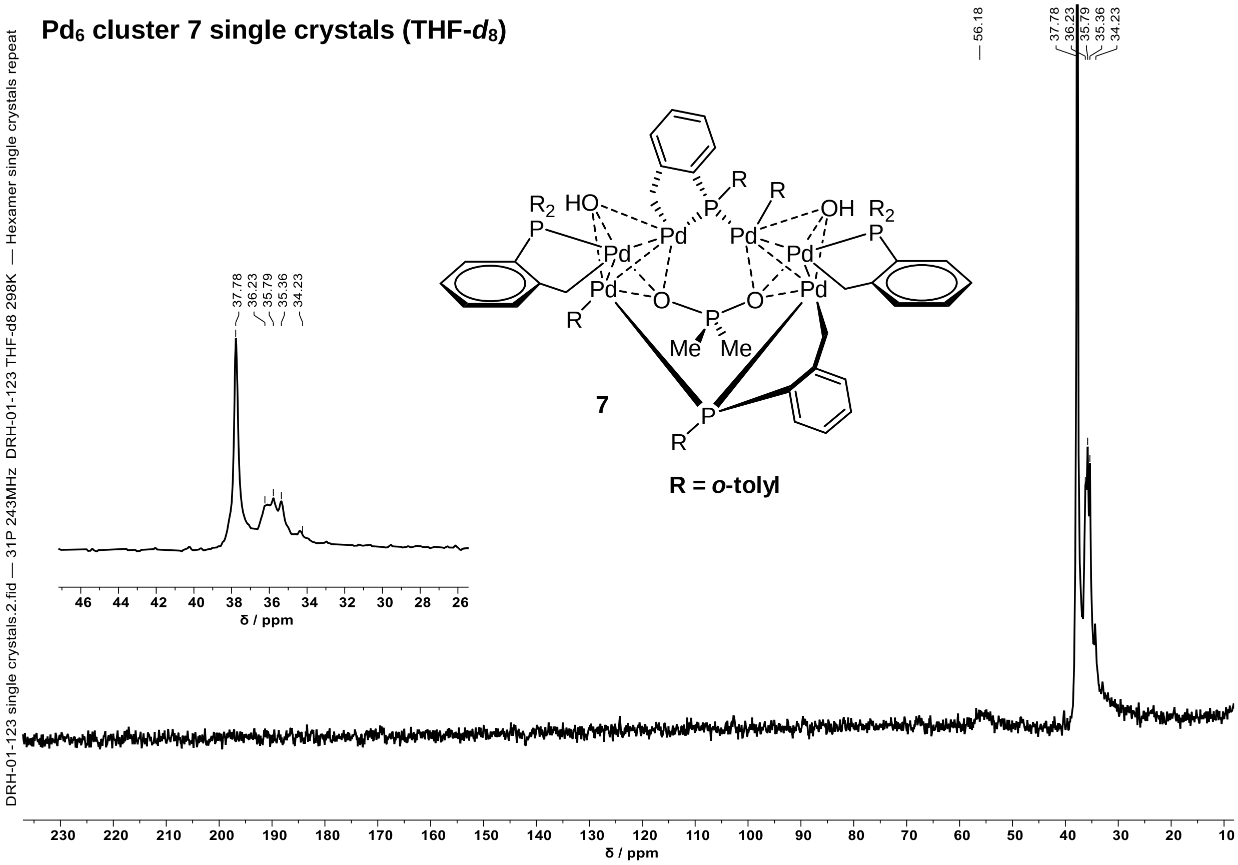

Figure 11: ^{31}P **NMR (243 MHz, THF-*d*$_8$, 51200 scans, 298 K) spectrum of the Pd$_6$ cluster 7. Lab book ref. DRH-01-123**

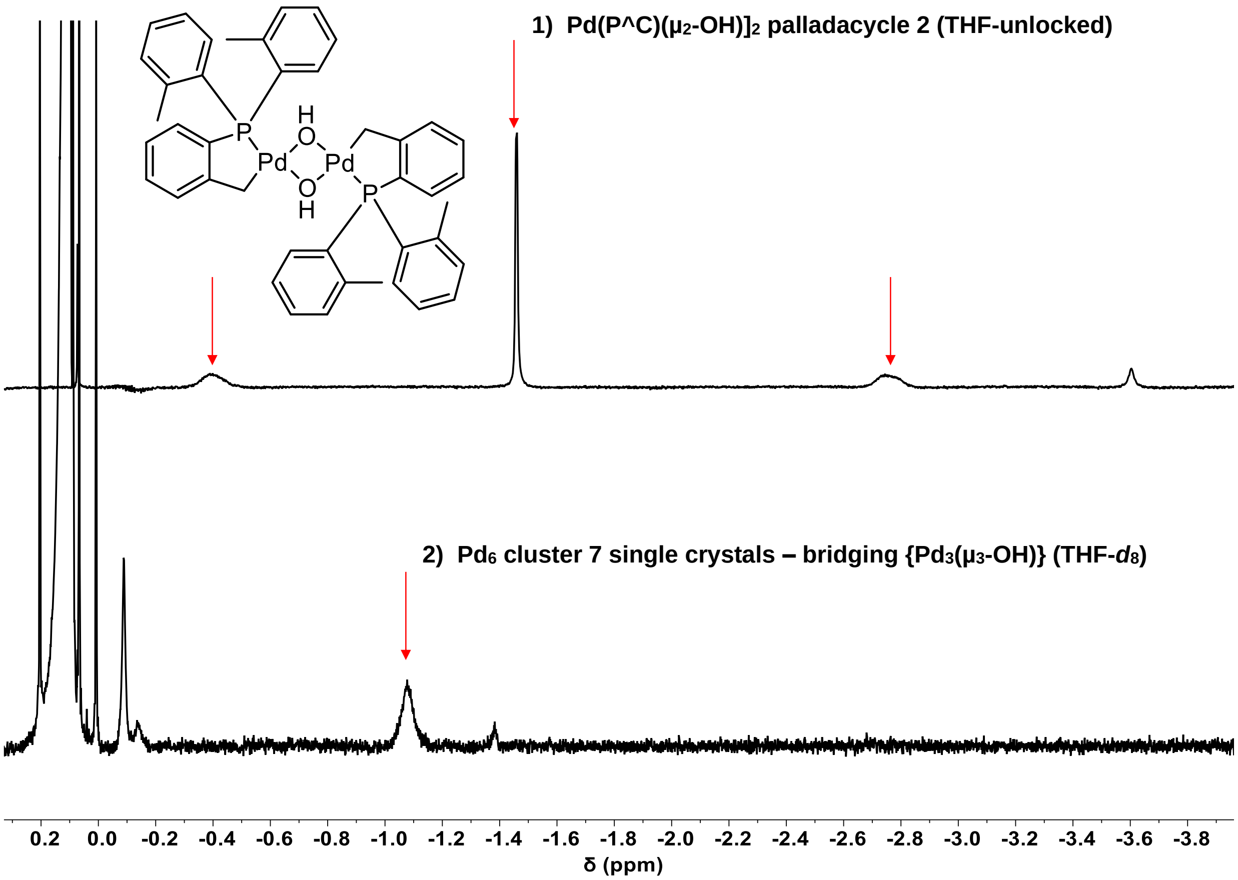

Figure 12: ^{1}H NMR (600 MHz, THF-unlocked, 298 K) spectrum of the [Pd(P^C)(μ_2-OH)]$_2$ palladacycle 2 (top) and (600 MHz, THF-*d*$_8$, 298 K) Pd_6 cluster 7 (bottom). The unlocked spectrum was referenced to the residual solvent peaks of THF-*d*$_8$ (3.58 ppm). The relevant OH proton peaks are highlighted for each spectrum.

Synthesis of palladacyclic Pd_8 8

Lab book ref. DRH-03-32, DRH-03-32-2

[Pd(P^C)(μ_2-OH)]$_2$ palladacycle **2** (5 mg, 0.00586 mmol, 1 equiv.) and octafluoronapthalene (1.6 mg, 0.00586 mmol, 1 equiv.) were dissolved in THF (0.40 mL, dry, degassed) in a J-Young tap NMR tube under a N_2 atmosphere. The tube was cooled to -78 °C, and 0.10 mL of a stock solution of 4-fluorophenylboronic acid and boroic acid in THF (16.4 mg, 7.2 mg in 1.0 mL dry, degassed THF, 2 equiv., 4 equiv. after addition) was added to the vessel. The tube was sealed under a N_2 atmosphere and vortexed at -78 °C, not shaken, to ensure mixing. The sample was loaded into a pre-cooled NMR spectrometer (500 MHz) at 243 K, and analysed by ^{19}F NMR timecourse (the data is not reported here, as it is not relevant to the cluster formation) over 18 h. After this time, the sample was warmed to room temperature and a ^{31}P NMR spectrum was taken. To see if an active $Pd^{(0)}$ species was present, 4-fluorobromobenzene (1.3 μL, 0.0047 mmol, 0.8

equiv.) was added to the NMR tube under N_2 and the ^{31}P NMR spectrum was recorded over several hours, showing no change. The reaction mixture was set up for crystallisation by slow vapour diffusion (THF / pentane, 0.25 mL : 1.5 mL) in a sealed vial under air, and after 17 months a small number of yellow crystals suitable for XRD (< 1 mg) were obtained, along with a quantity of amorphous solid.

It would appear palladacyclic cluster **8** formed from a proposed [Pd(P^C)]$_2$ bipalladacycle reacting with a "Pd$^{(0)}$" species and abstracting a fluoride from octafluoronapthalene. The broad peak between ^{31}P δ 40 – 32 ppm after the NMR timecourse at -30 °C is likely representative of **8** and did not change upon the addition of 4-fluorobromobenzene. In the 19F NMR spectrum, a small peak at δ -131.3 ppm can be observed, which could be indicative of a deshielded F in the tetrahedral geometry observed in **8**. The stabilizing $[H_2B_2O_5]^{2-}$ group is from the stabilising boric acid additive.

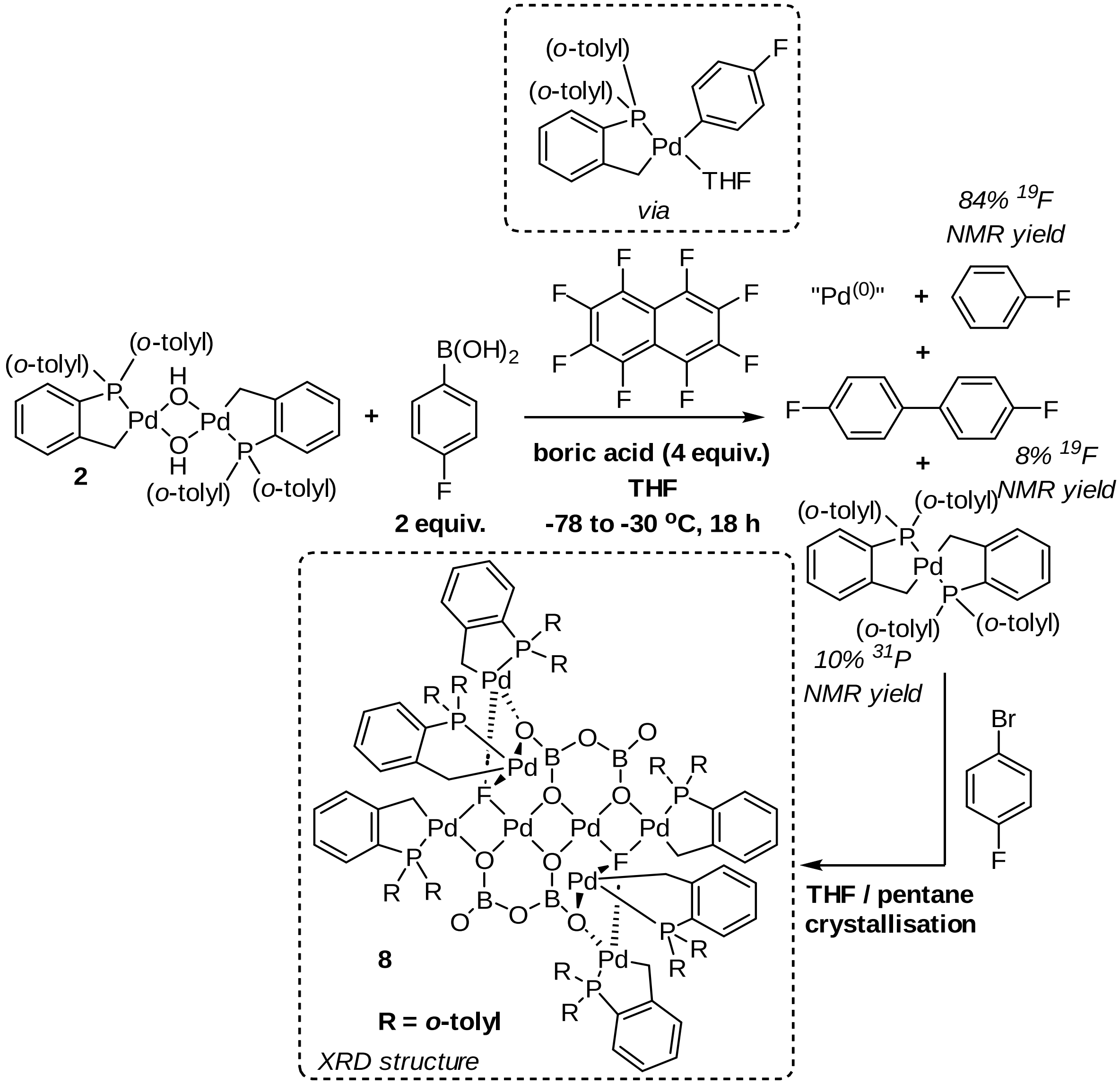

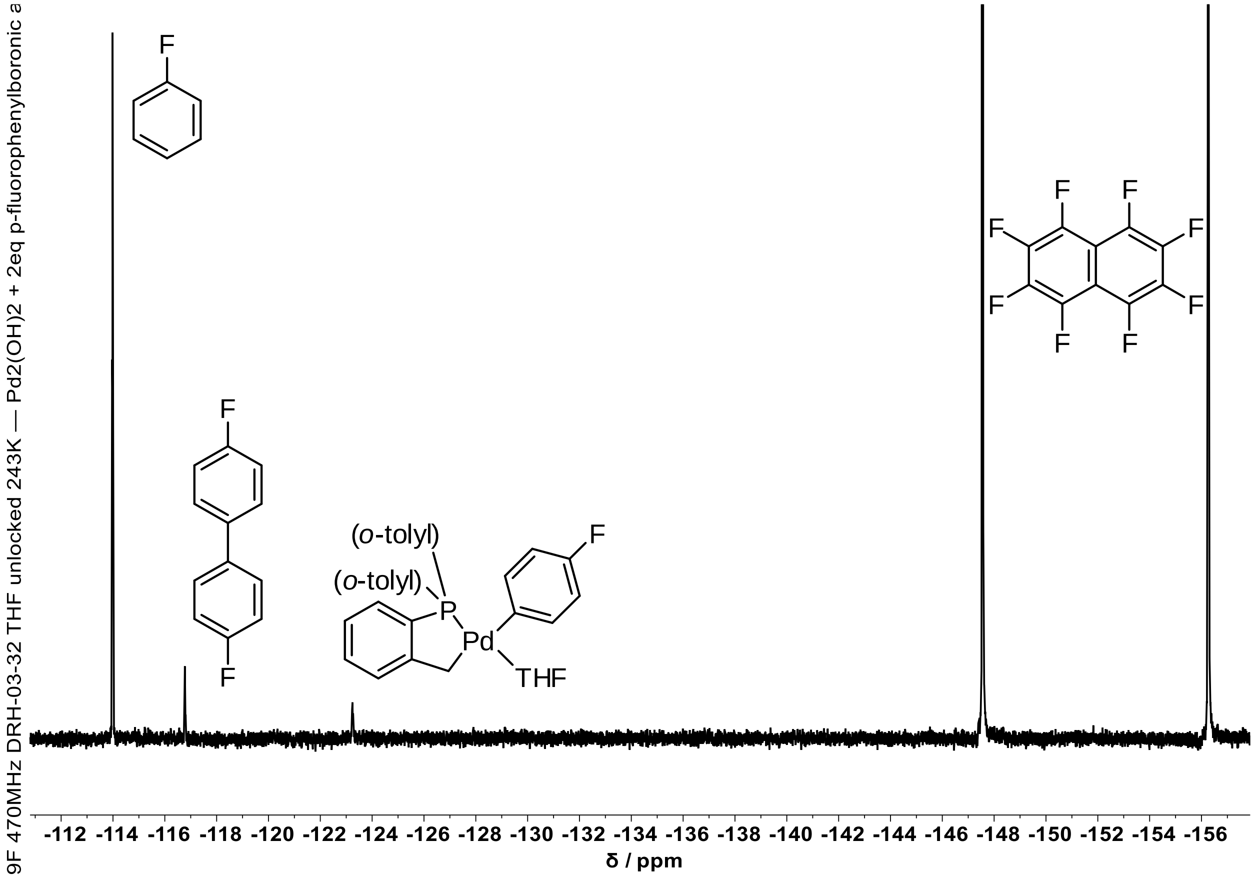

Figure 13: ^{19}F NMR (470 MHz, THF-unlocked, 243 K) spectrum of the reaction mixture potentially containing Pd_8 cluster after the kinetic time course (fluorobenzene = 84%, biaryl = 8%, Pd intermediate = 8%). Lab book ref. DRH-03-32 (162)

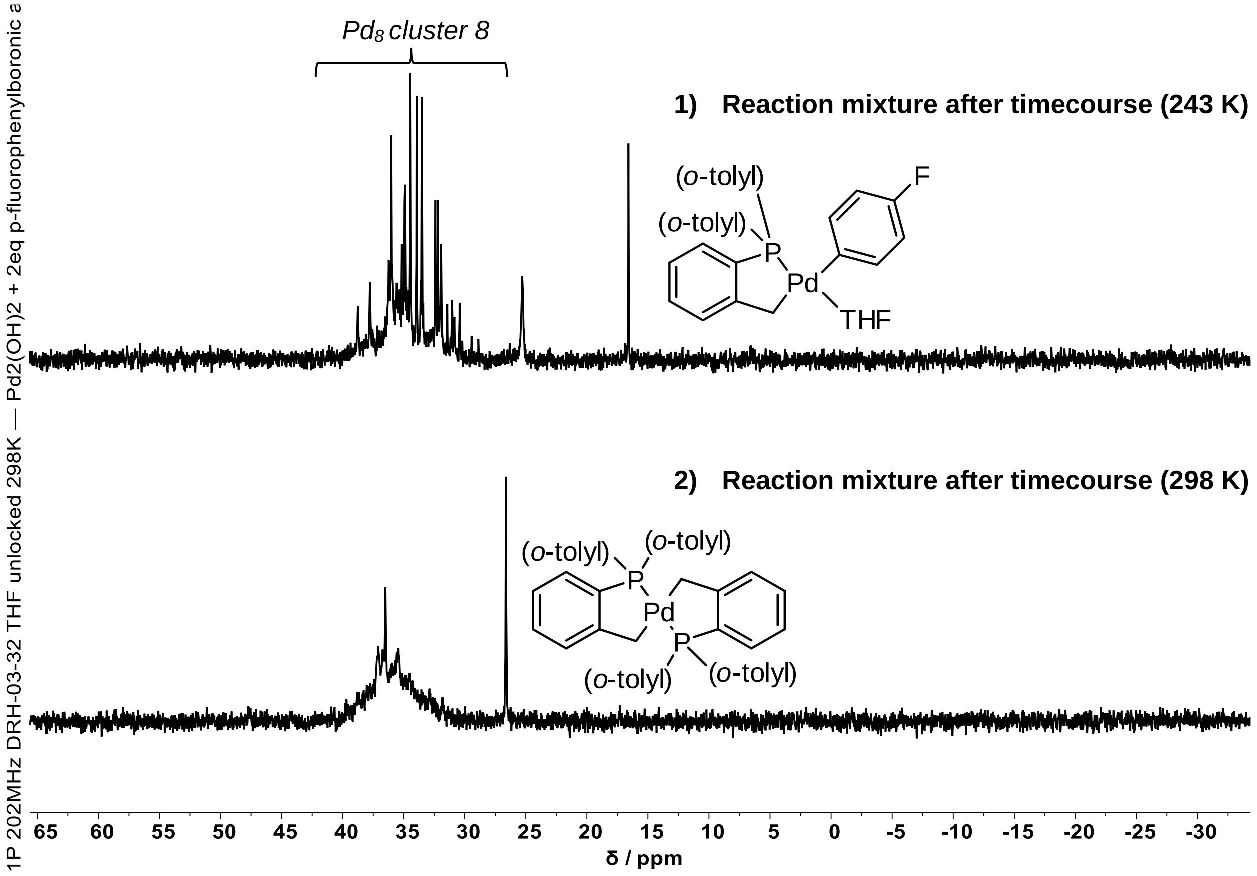

Figure 14: Stacked ^{31}P NMR (202 MHz, THF-unlocked) spectrum of the reaction mixture potentially containing Pd_8 cluster 8, 1) after the kinetic time course at 243 K (integral of 40 – 28 ppm region represents 90% of the ^{31}P environments, Pd intermediate = 3%), 2) the same sample at 298 K (integral of 40 – 30 ppm region represents 93% of the ^{31}P environments). Lab book ref. DRH-03-32 (163, 167)

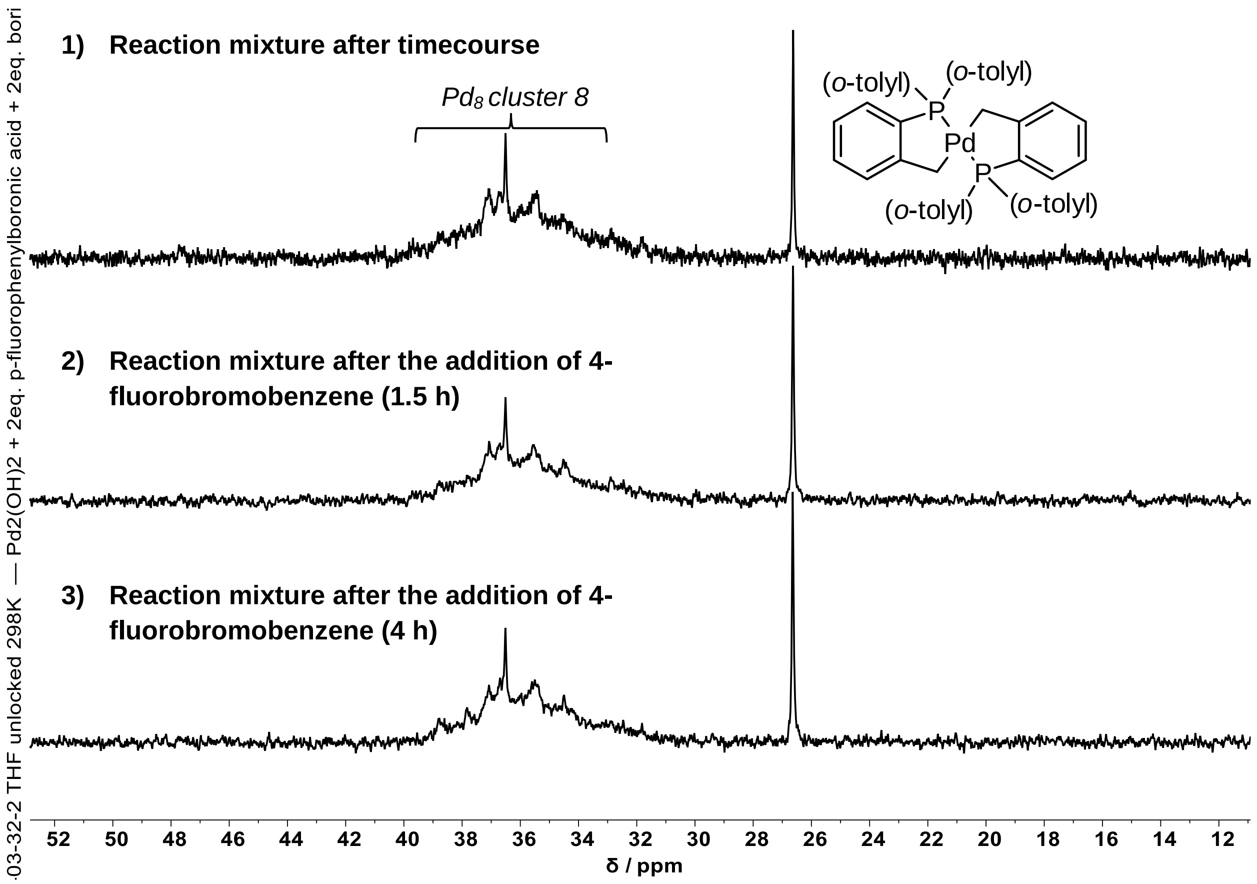

Figure 15: Stacked ^{31}P NMR ((top 202 MHz, others 243 MHz), THF-unlocked, 298 K) spectrum of the reaction mixture potentially containing Pd_8 cluster 8 1) after the kinetic time course at -30 °C, 2) after the addition of 4-fluorobromobenzene (1.5 h), 3) the same sample after 4 h. Lab book ref. DRH-03-32, DRH-03-32-2

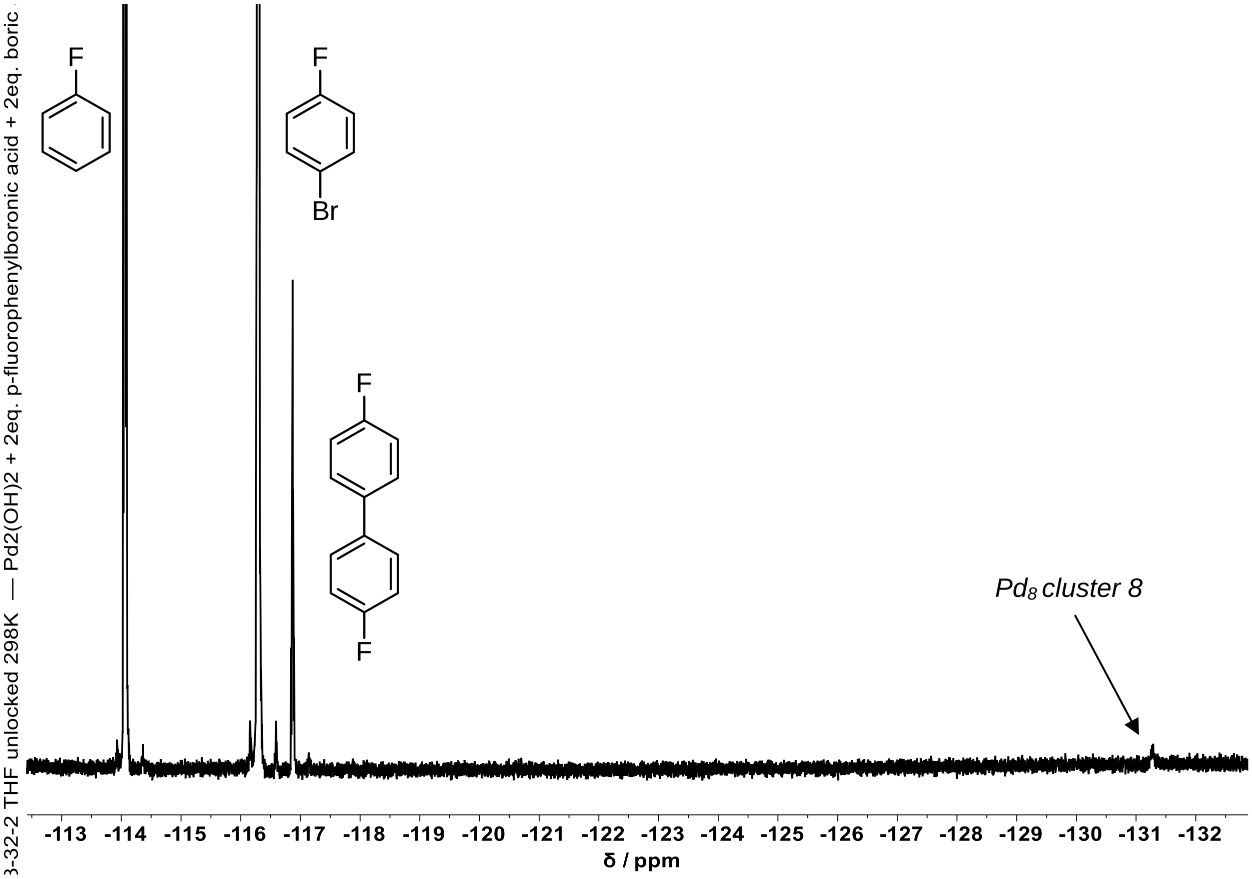

Figure 16: ^{19}F NMR (565 MHz, THF-unlocked, 298 K) spectrum of the reaction mixture containing Pd_8 cluster 8 following addition of 4-fluorobromobenzene after 4 days. Lab book ref. DRH-03-32-2

3. *In situ* IR Data – Methodology and Kinetic profiles

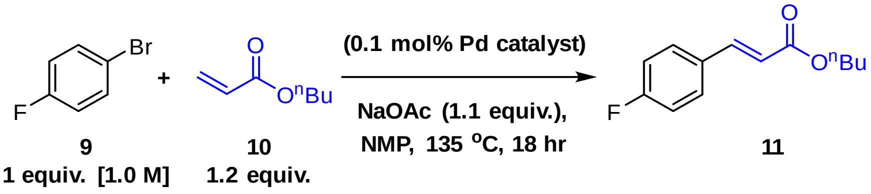

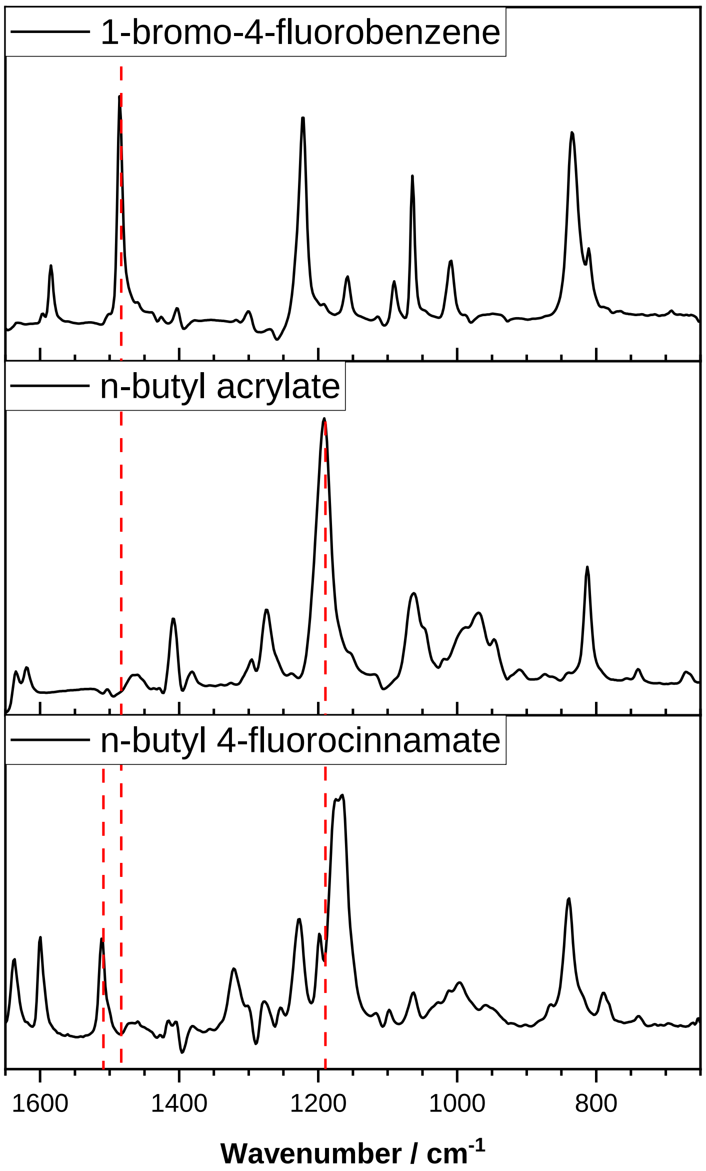

Figure 17: Reference spectra of (top) 1-bromo-4-fluorobenzene (9), (middle) n-butyl acrylate (10) (bottom), n-butyl 4-fluorocinnamate product (11) in NMP solvent. Spectra taken using ReactIR 15 (with Diamond probe) at 23 °C. The key peaks that are followed by IR during the reaction are indicated by the dashed red lines (1484 cm^{-1} for 1-bromo-4-fluorobenzene 9, 1190 cm^{-1} for n-butyl acrylate 10 and 1509 cm^{-1} for n-butyl 4-fluorocinnamate 11.

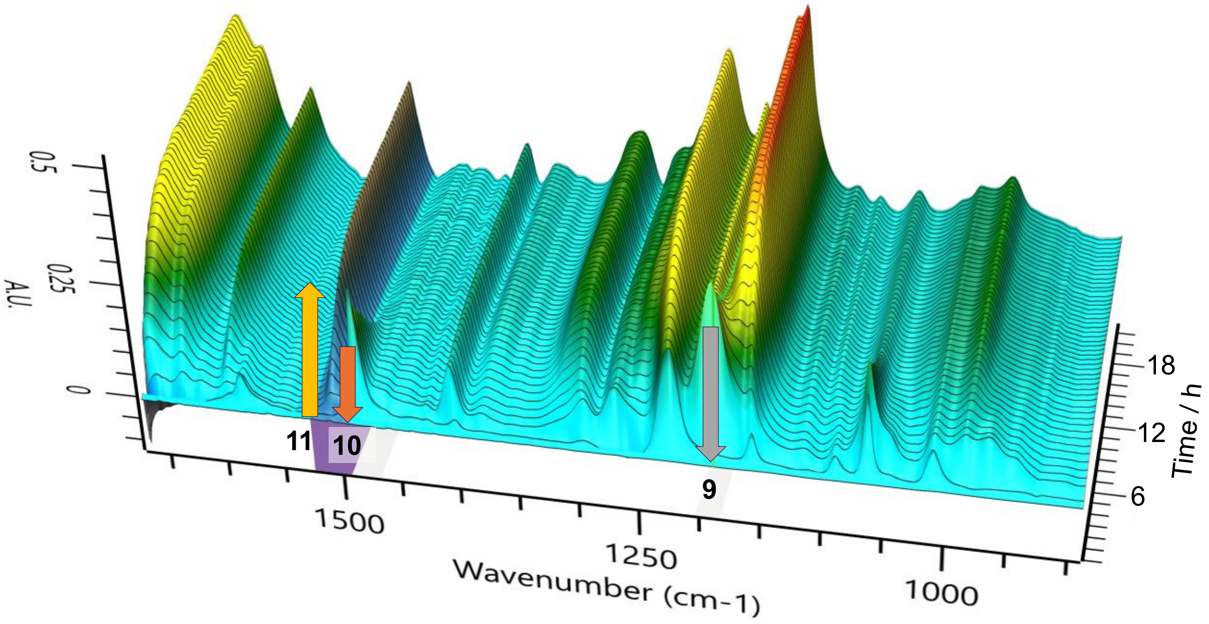

Figure 18: ***in situ*** **IR compilation surface generated by iC IR 7.1 software for the Heck reaction (lab book ref. DRH-01-144)**

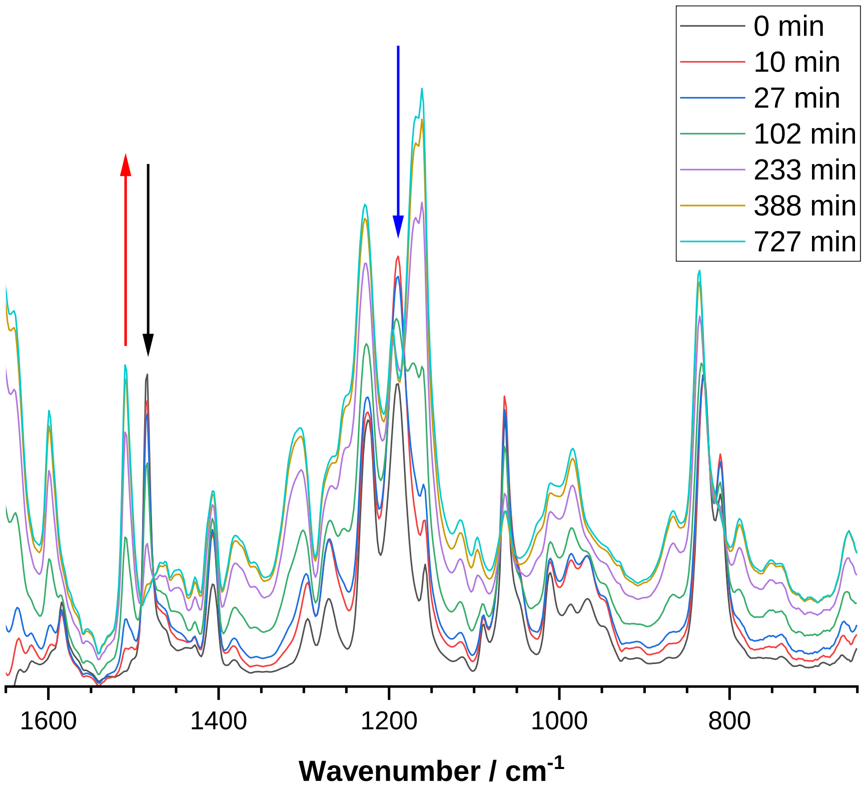

Figure 19: Selected spectra for the Heck reaction (lab book ref. DRH-01-144) over the course of the reaction. The reaction was sampled every 1 min. The direction of key peaks are indicated by arrows (blue for n-butyl acrylate 10 (1190 cm^{-1}), black for 1-bromo-4-fluorobenzene 9 (1484 cm^{-1}) and red for n-butyl 4-fluorocinnamate 11 (1509 cm^{-1})).

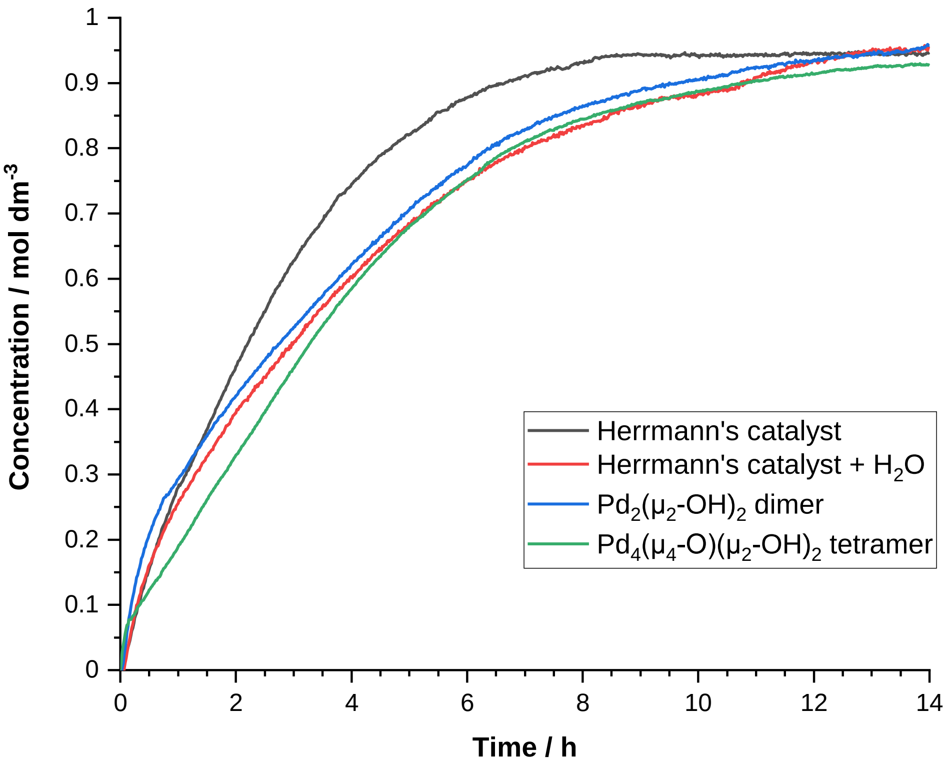

Figure 20: Comparison of Heck reactions (product 11 formation) with different pre-catalysts (General Procedure 2.1)

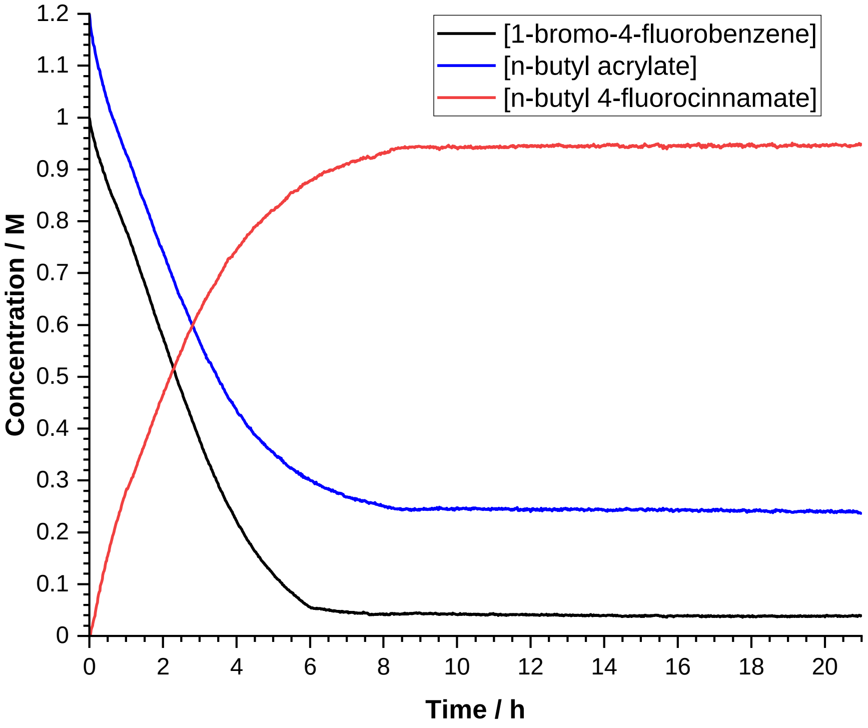

Figure 21: Heck reaction catalysed with commercial Herrmann's catalyst 1 (General Procedure 2.1). Lab book ref. DRH-01-144

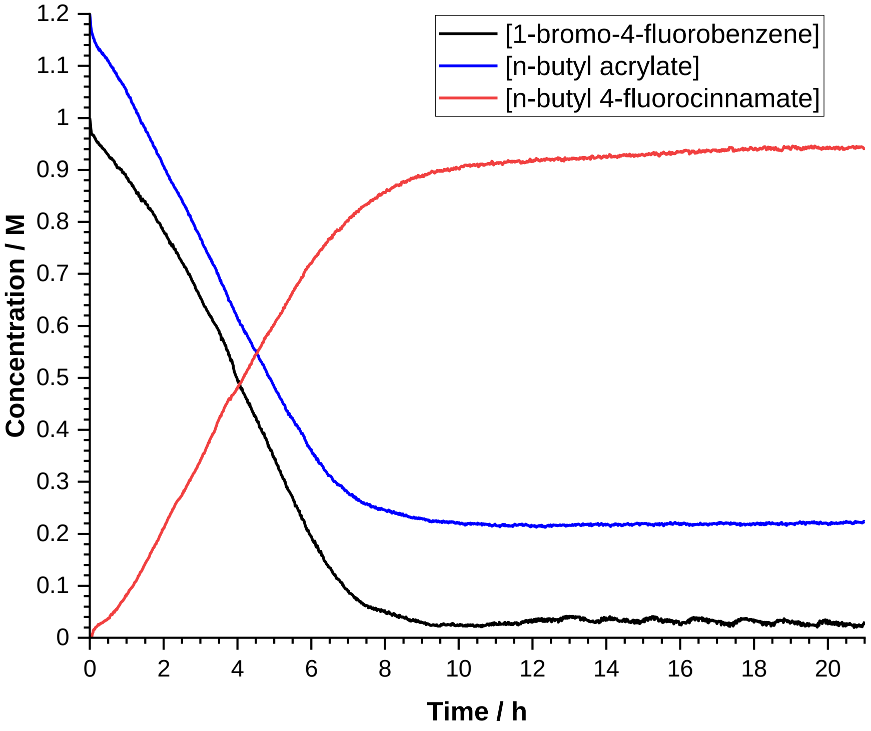

Figure 22: Heck reaction catalysed by commercial Herrmann's catalyst 1 with 50 μL water added (General Procedure 2.1). Lab book ref. DRH-01-145

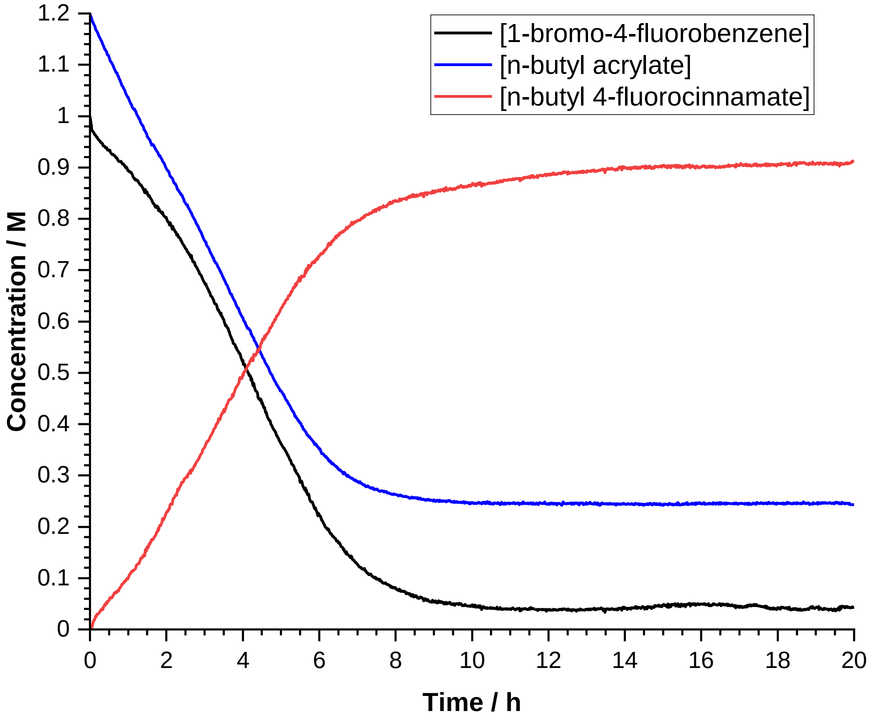

Figure 23: Heck reaction catalysed by $[Pd(P^\wedge C)(\mu_2\text{-}OH)]_2$ palladacycle 2 (General Procedure 2.1). Lab book ref. DRH-01-151

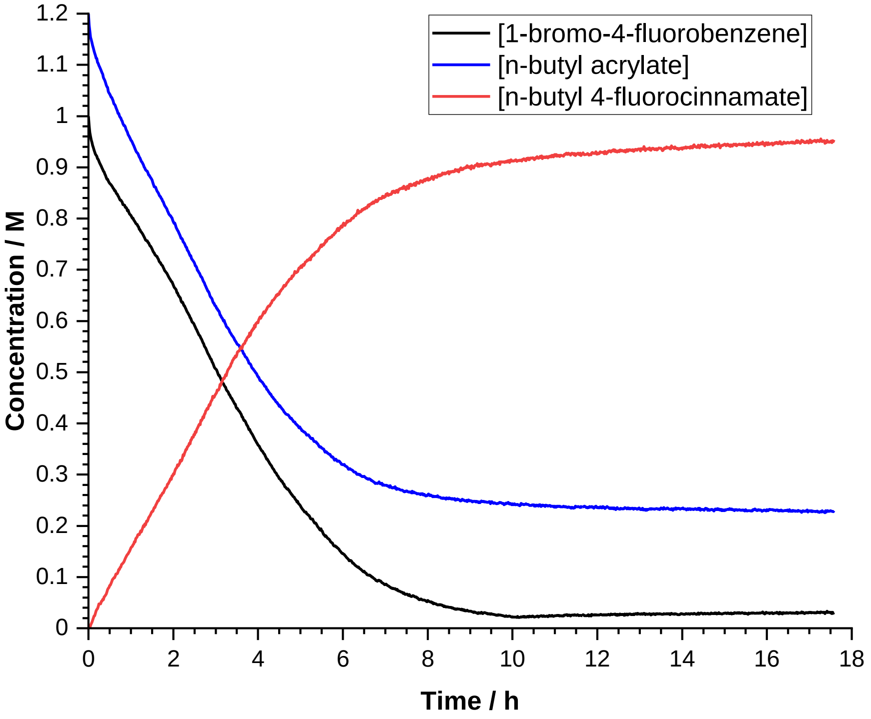

Figure 24: Heck reaction catalysed by palladacyclic Pd_4 cluster 3 (General Procedure 2.1). Lab book ref. DRH-01-146-2

4. X-Ray Crystallography

Diffraction data were collected at 110 K on an Oxford Diffraction SuperNova diffractometer with Cu-K_α radiation (λ = 1.54184 Å) or Mo-K_α radiation (λ = 0.71073), using an EOS CCD camera. The crystal was cooled with an Oxford Instruments Cryojet. Diffractometer control, data collection, initial unit cell determination, frame integration and unit-cell refinement was carried out with "Crysalis".[4] Face-indexed absorption corrections were applied using spherical harmonics, implemented in SCALE3 ABSPACK scaling algorithm.[5] OLEX2[6] was used for overall structure solution and refinement . Within OLEX2, the algorithm used for structure solution was "ShelXT dual-space".[7] Refinement was carried out by full-matrix least-squares used the SHELXL-97[7] algorithm within OLEX2. All non-hydrogen atoms were refined anisotropically. Crystalmaker® software was used to visualise the structures as well as generating the figures presented herein.

ijsf21009: Palladacyclic $Pd_4(\mu_4\text{-}O)(\mu_2\text{-}OH)_2$ Cluster 3 CCDC 2288572 (lab book ref. DRH-01-131)

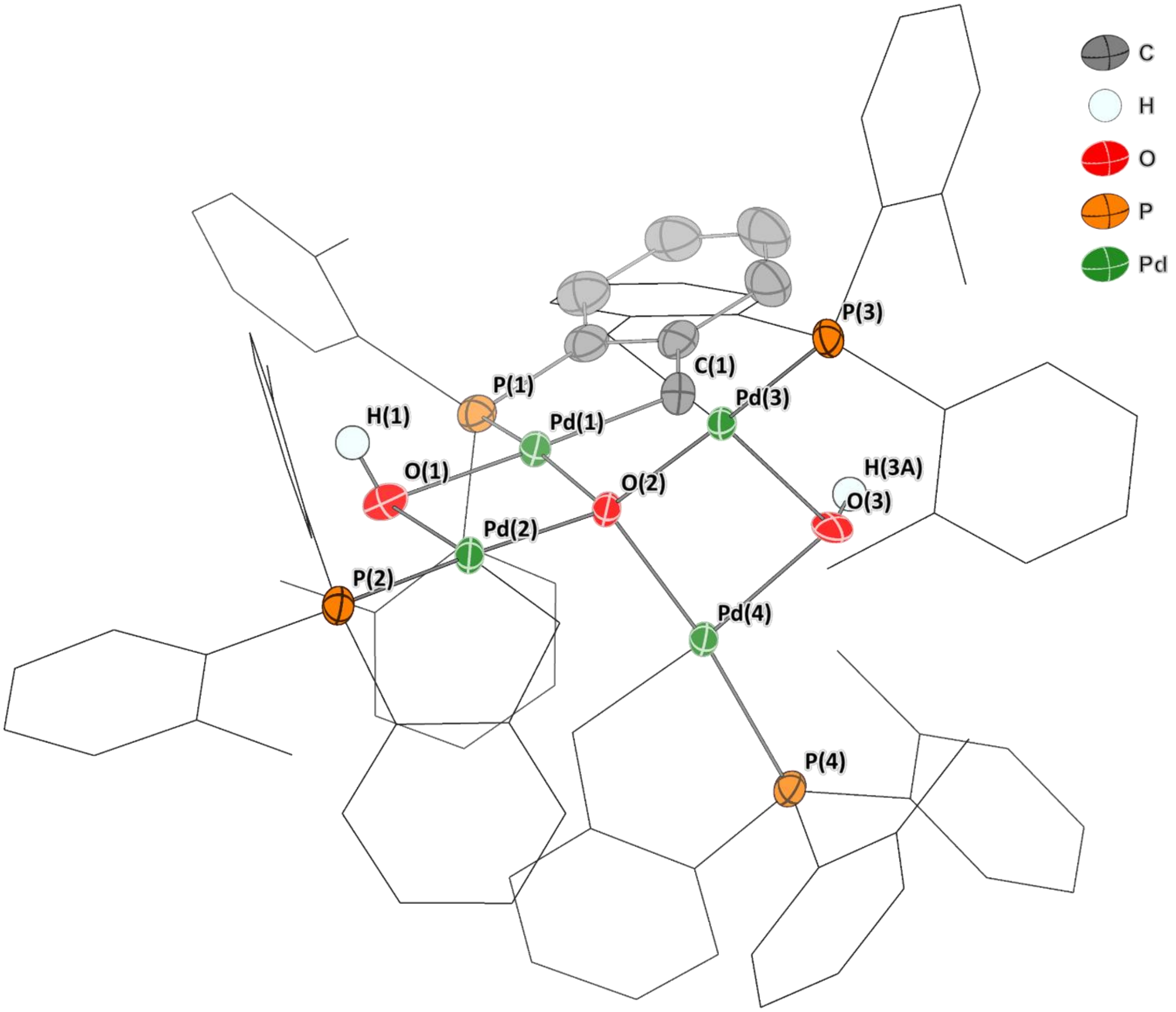

Figure 25: Structure, obtained by X-ray diffraction, of a single crystal of di-*cis*-(μ_2-hydroxido)(μ_4-oxido) di[*o*-(di-*o*-tolylphosphino)benzyl] dipalladium(II) 3 (thermal ellipsoids are set at 50% probability, selected H-atoms are omitted for clarity, for structural clarity most carbon atoms are displayed as a wireframe structure). Selected interatomic lengths /Å: Pd1-C1 = 2.032(4); Pd1-P1 = 2.1841(9); Pd1-O1 = 2.202(2); Pd1-O2 = 2.070(2); Pd1-Pd2 = 3.1785(4). Selected interatomic angles /°: P1-Pd1-C1 = 84.52(12); P1-Pd1-O1 = 102.74(18); Pd1-O1-Pd2 = 92.89(10); Pd1-O2-Pd2 = 99.66(10); Pd3-O2-Pd4 = 97.35(9).

Refinement Special Details

Hydroxyl hydrogens were restrained in position with an O-H bond length of 0.87 angstroms and an H-Pd distance of 2.57 angstroms.

Table 2: X-Ray Diffraction Data for the Pd_4 cluster 3

Identification code	ijsf21009
Empirical formula	$C_{84}H_{82}O_3P_4Pd_4$
Formula weight	1688.97
Temperature/K	110.00(10)
Crystal system	triclinic
Space group	P-1
a/Å	13.6277(4)
b/Å	14.8406(4)
c/Å	20.3507(5)
α/°	98.300(2)
β/°	93.130(2)
γ/°	116.152(3)
Volume/Å^3	3623.25(19)
Z	2
ρ_{calc}g/cm^3	1.548
μ/mm^{-1}	9.099
F(000)	1708.0
Crystal size/mm^3	0.13 × 0.07 × 0.04
Radiation	Cu Kα (λ = 1.54184)
2Θ range for data collection/°	6.756 to 134.16
Index ranges	-16 ≤ h ≤ 15, -14 ≤ k ≤ 17, -24 ≤ l ≤ 24
Reflections collected	25088
Independent reflections	12912 [R_{int} = 0.0291, R_{sigma} = 0.0404]
Data/restraints/parameters	12912/6/870
Goodness-of-fit on F^2	1.025
Final R indexes [I>=2σ (I)]	R_1 = 0.0308, wR_2 = 0.0740
Final R indexes [all data]	R_1 = 0.0378, wR_2 = 0.0777
Largest diff. peak/hole / e Å^{-3}	1.05/-0.68

ijsf22044: Palladaycyclic $Pd_4(\mu_2\text{-}Cl)_2(\mu_4\text{-}O)$ Cluster 5 CCDC 2288580 (lab book ref. DRH-03-75)

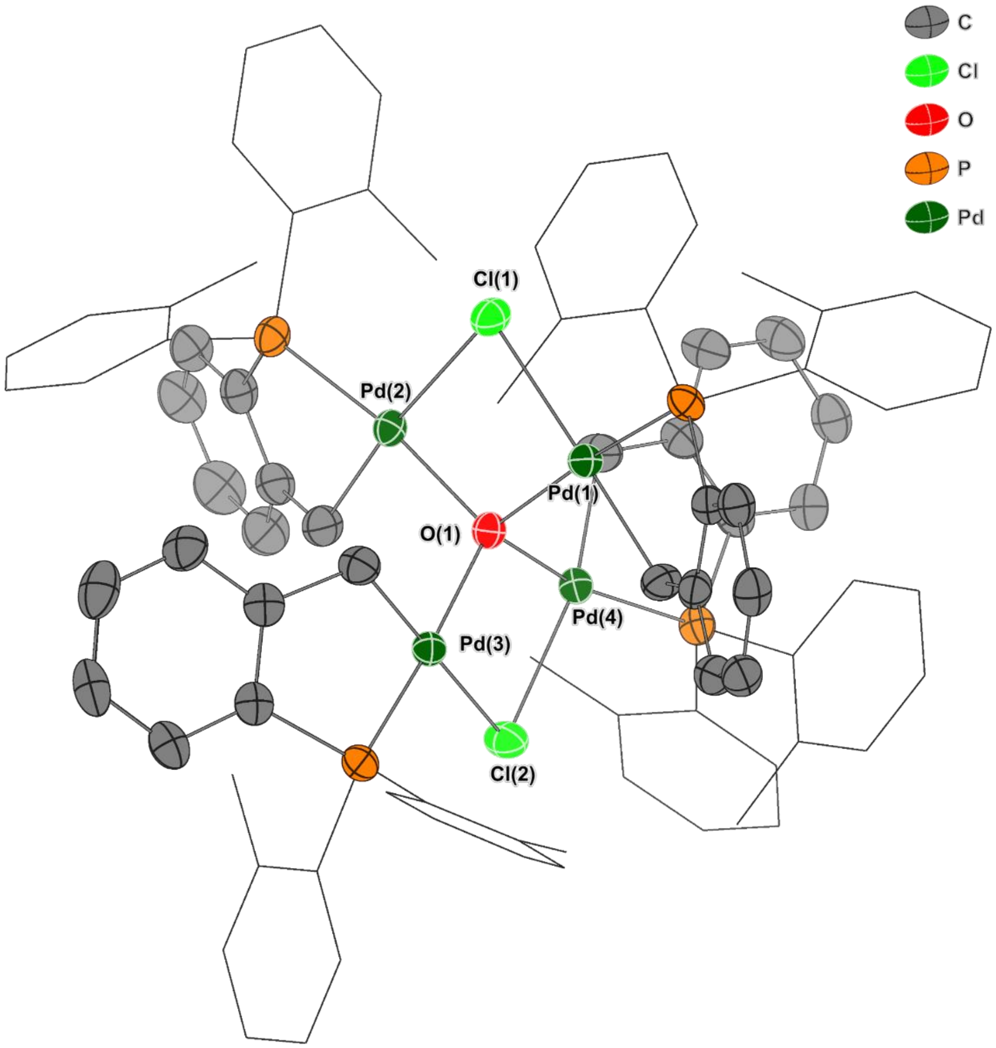

Figure 26: Structure, obtained by X-ray diffraction, of a single crystal of $Pd_4(\mu_2\text{-}Cl)_2(\mu_4\text{-}O)$ cluster 5 (thermal ellipsoids are set at 50% probability, H-atoms are omitted for clarity, for structural clarity most carbon atoms are displayed as a wireframe structure). Selected interatomic lengths /Å: Pd1-Cl1 = 2.4737(8), Pd1-O1 =2.101(2), Pd1-P1 = 2.2093(9), Pd1-C1 = 2.052(3). Selected interatomic angles /°: Pd1-Cl1-Pd2 = 84.35(3), Pd3-Cl2-Pd4 = 84.67(3), Pd1-O1-Pd4 = 103.46(9), Pd2-O1-Pd3 = 113.65(10).

Refinement special details

One of the tolyl groups was disordered and modelled in two positions with refined occupancies of 0.73:0.23(1). The aromatic ring of the minor form was constrained to be a regular hexagon with a C-C bond length of 1.39 angstroms. There was also a site containing a mixture of dichloromethane and pentane in a refined ratio of 0.615:0.385(5). For the pentane, the C-C bond lengths were restrained to be 1.53 angstroms and the C-C-C bond distances to be 2.48 angstroms.

Table 3: X-Ray data for $Pd_4(\mu_2\text{-}Cl)_2(\mu_4\text{-}O)$ cluster 5

Identification code	ijsf22044
Empirical formula	$C_{86.92}H_{86.43}Cl_{4.89}OP_4Pd_4$
Formula weight	1869.86
Temperature/K	110.00(10)
Crystal system	triclinic
Space group	P-1
a/Å	13.9902(2)
b/Å	15.1119(2)
c/Å	19.6630(3)
α/°	82.3168(14)
β/°	83.1543(13)
γ/°	82.3307(13)
Volume/Å^3	4061.14(12)
Z	2
ρ_{calc}g/cm^3	1.529
μ/mm^{-1}	9.604
F(000)	1886.0
Crystal size/mm^3	0.149 × 0.043 × 0.012
Radiation	Cu Kα (λ = 1.54184)
2Θ range for data collection/°	7.046 to 134.16
Index ranges	-14 ≤ h ≤ 16, -18 ≤ k ≤ 15, -23 ≤ l ≤ 23
Reflections collected	50244
Independent reflections	14484 [R_{int} = 0.0426, R_{sigma} = 0.0399]
Data/restraints/parameters	14484/20/952
Goodness-of-fit on F^2	1.023
Final R indexes [I>=2σ (I)]	R_1 = 0.0333, wR_2 = 0.0859
Final R indexes [all data]	R_1 = 0.0384, wR_2 = 0.0888
Largest diff. peak/hole / e Å^{-3}	1.10/-0.86

ijsf21006a: Palladacyclic Pd_6 Cluster 7 CCDC 2288584 (lab book ref. DRH-01-123)

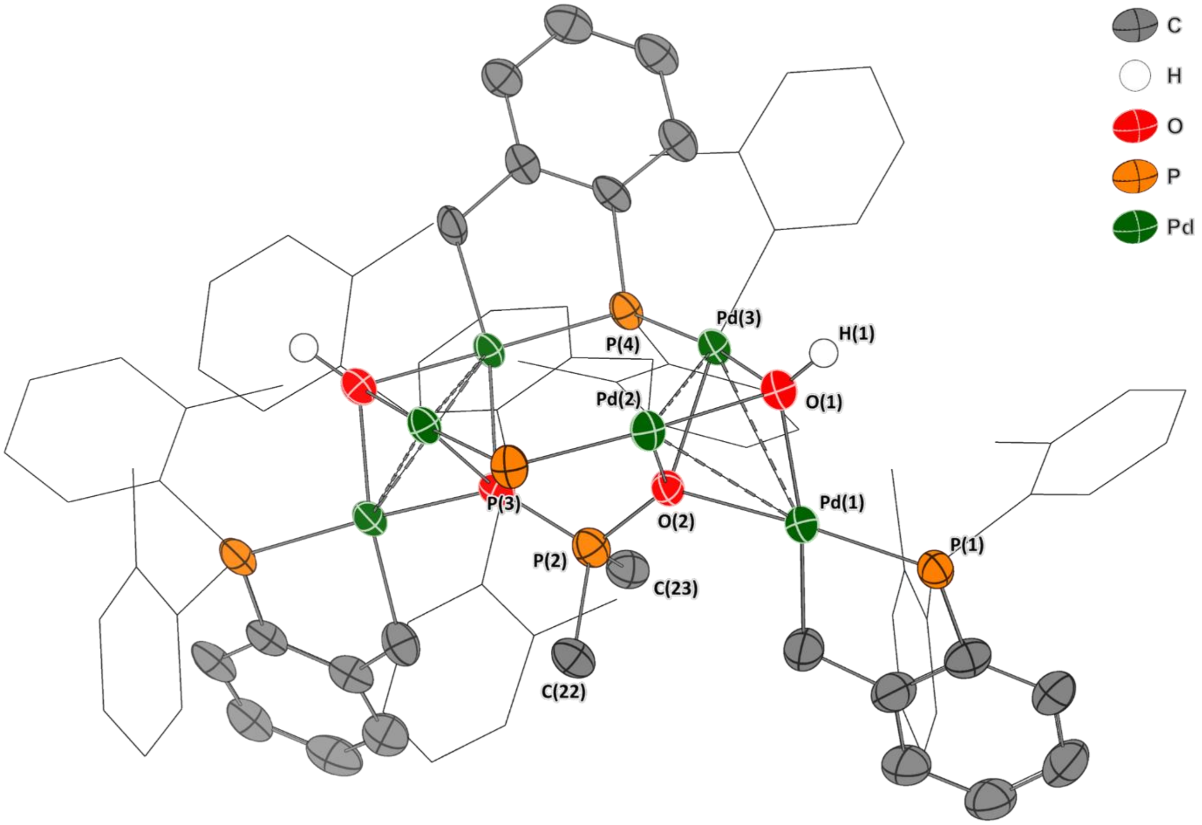

Figure 27: Structure, obtained by X-ray diffraction, of a single crystal of the Palladacyclic Pd_6 Cluster 7 (thermal ellipsoids are set at 50% probability, selected H-atoms are omitted for clarity, for structural clarity most carbon atoms are displayed as a wireframe structure). The unit cell contained two hexameric species and nine tetrahydrofurans of crystallization. Selected bond lengths (Å) and angles (°); Pd1-P1 = 2.201(2); Pd1-O1 = 2.141(6); Pd1-Pd2 = 3.0422(9); Pd2-P3 = 2.218(2); Pd3-P4 = 2.238(2); O2-P2 = 1.631(6); P2-C22 = 1.878(9); Pd1-Pd2-Pd3 = 59.30(2); O1-Pd1-O2 = 70.5(2); Pd1-O2-P2 = 129.7(3); C22-P2-C23 = 109.7(4).

Refinement Special Details

The presence of a large number of relatively high residual density peaks close to the palladium atoms is believed to be due to twinning. Unfortunately, this could be resolved with neither a non-merohedral nor a pseudo-merohedral approach.

The ADP of 4 carbons in one phenyl ring and a number of carbons were restrained to be approximately isotropic. 4 very disordered THFs were modelled using a solvent mask rather than a discrete atom model.

Table 4: X-Ray Diffraction data for the Palladacyclic Pd_6 Cluster 7

**Identification code**	**ijsf21006a**
**Empirical formula**	$C_{94}H_{104}O_6P_5Pd_6$
**Formula weight**	2123.02
**Temperature/K**	110.00(10)
**Crystal system**	triclinic
**Space group**	P-1
**a/Å**	15.4929(2)
**b/Å**	20.2644(3)
**c/Å**	32.5025(5)
**α/°**	75.5537(14)
**β/°**	85.6199(13)
**γ/°**	84.3876(13)
**Volume/Å^3**	9820.0(3)
**Z**	4
**ρ_{calc}g/cm^3**	1.436
**μ/mm^{-1}**	9.814
**F(000)**	4268.0
**Crystal size/mm^3**	0.232 × 0.179 × 0.02
**Radiation**	Cu Kα (λ = 1.54184)
**2Θ range for data collection/°**	7.01 to 134.156
**Index ranges**	-18 ≤ h ≤ 18, -23 ≤ k ≤ 24, -38 ≤ l ≤ 32
**Reflections collected**	95656
**Independent reflections**	34970 [R_{int} = 0.0570, R_{sigma} = 0.0565]
**Data/restraints/parameters**	34970/60/2019
**Goodness-of-fit on F^2**	1.046
**Final R indexes [I>=2σ (I)]**	R_1 = 0.0645, wR_2 = 0.1806
**Final R indexes [all data]**	R_1 = 0.0750, wR_2 = 0.1890
**Largest diff. peak/hole / e Å^{-3}**	3.91/-1.58

ijsf23078: Palladacyclic Pd_8 Cluster Complex 8 CCDC 2308805 (lab book ref. DRH-03-32-2)

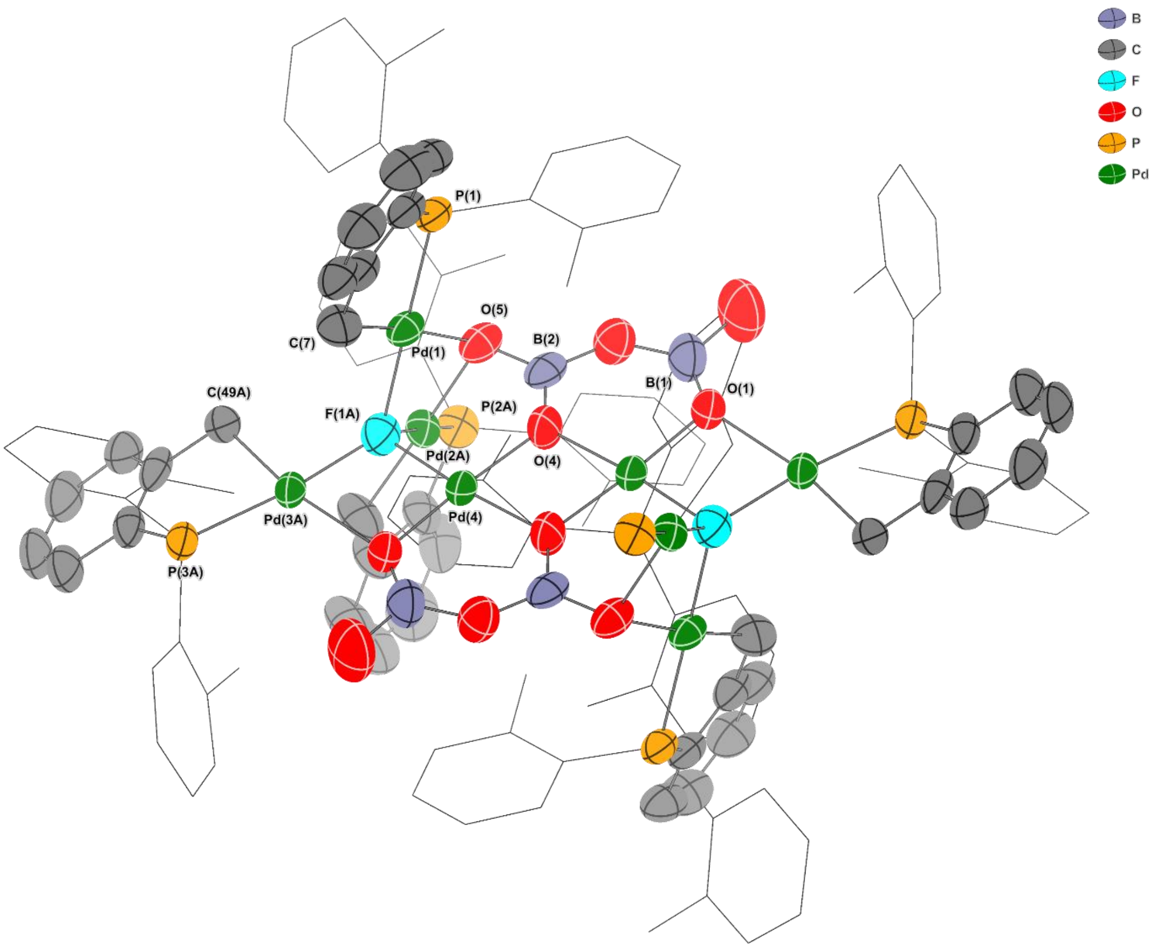

Figure 28: Structure, obtained by X-ray diffraction, of a single crystal of a palladacyclic Pd_8 cluster complex 8 (thermal ellipsoids are set at 50% probability, H-atoms are omitted for clarity). Due to high levels of disorder, only one isomer is displayed, and selected carbon atoms are displayed as wireframe structures for clarity. Selected interatomic lengths /Å: P1-Pd1 = 2.1856(10); Pd1-C7 = 2.024(5); Pd1-F1A = 2.02(3); Pd2A-F1A = 2.10(2); Pd3A-F1A = 2.06(3); Pd4-F1A = 2.04(3); Pd4-O1 = 1.991(3); Pd4-O4 = 1.980(3). Selected interatomic angles /°: O4-Pd4-O1 = 95.56(13); Pd1-F1A-Pd2A = 98.7(10); Pd1-F1A-Pd4 = 97.7(13); Pd2A-F1A-Pd3A = 131.2(14); Pd1-F1A-Pd3A = 120.6(13); P1-Pd1-C7 = 83.77(14).

Refinement Special Details

The structure exhibited considerable disorder. All but one benzyl was modelled in two positions with equal occupancy (free refinement of occupancies revealed a 1:1 ratio). In addition, two palladiums, two phosphorus and the fluorine were also modelled in two positions with equal occupancy. The ADPs of each pair of disordered atoms were constrained to be equal (e.g. Pd2a & Pd2b, C8a & C8b). 5 aromatic rings were constrained to be regular hexagons with a C-C bond length of 1.39 angstroms (8a-13a, 8b-13b, 15b-20b, 29a-34a, 29b-24b). The crystal contained highly disordered solvent for which a discrete atom model could not be obtained. Use of a solvent mask gave an estimated void volume of 170 cubic angstroms

containing 42 electrons per asymmetric unit. This could be either a tetrahydrofuran, a pentane or a mixture of both.

Table 5: X-Ray Diffraction Data for the palladacyclic Pd_8 cluster complex 8

Identification code	ijsf23078
Empirical formula	$C_{63}H_{60}B_2FO_5P_3Pd_4$
Formula weight	1456.24
Temperature/K	110.00(10)
Crystal system	triclinic
Space group	P-1
a/Å	15.0801(3)
b/Å	15.0825(3)
c/Å	16.2726(2)
α/°	79.7260(10)
β/°	74.1320(10)
γ/°	63.091(2)
Volume/Å^3	3168.19(11)
Z	2
ρ_{calc}g/cm^3	1.527
μ/mm^{-1}	10.116
F(000)	1452.0
Crystal size/mm^3	0.104 × 0.067 × 0.044
Radiation	Cu Kα (λ = 1.54184)
2Θ range for data collection/°	7.136 to 134.152
Index ranges	-14 ≤ h ≤ 18, -13 ≤ k ≤ 18, -19 ≤ l ≤ 19
Reflections collected	36300
Independent reflections	11292 [R_{int} = 0.0394, R_{sigma} = 0.0407]
Data/restraints/parameters	11292/103/862
Goodness-of-fit on F^2	1.052
Final R indexes [I>=2σ (I)]	R_1 = 0.0376, wR_2 = 0.1006
Final R indexes [all data]	R_1 = 0.0469, wR_2 = 0.1056
Largest diff. peak/hole / e Å^{-3}	1.34/-0.54

5. NMR Spectral Data for Organic and Inorganic Compounds

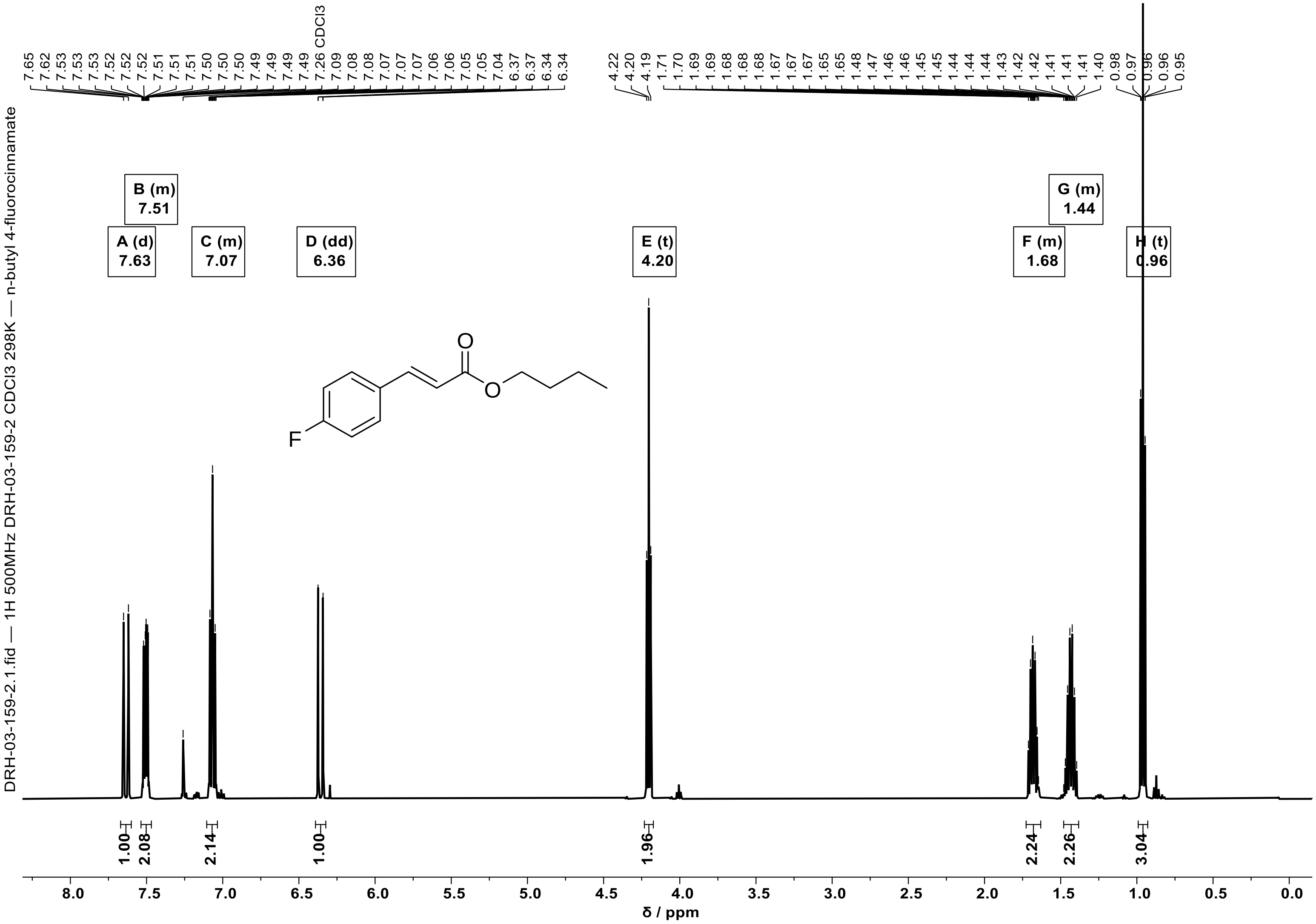

Figure 29: ^{1}H NMR (500 MHz, C*D*Cl$_3$, 32 scans, 298 K) spectrum of n-butyl 4-fluorocinnamate 11. 2% of the biaryl side-product (derived from 9) co-eluted, and is present as a small impurity. Lab book ref. DRH-03-159

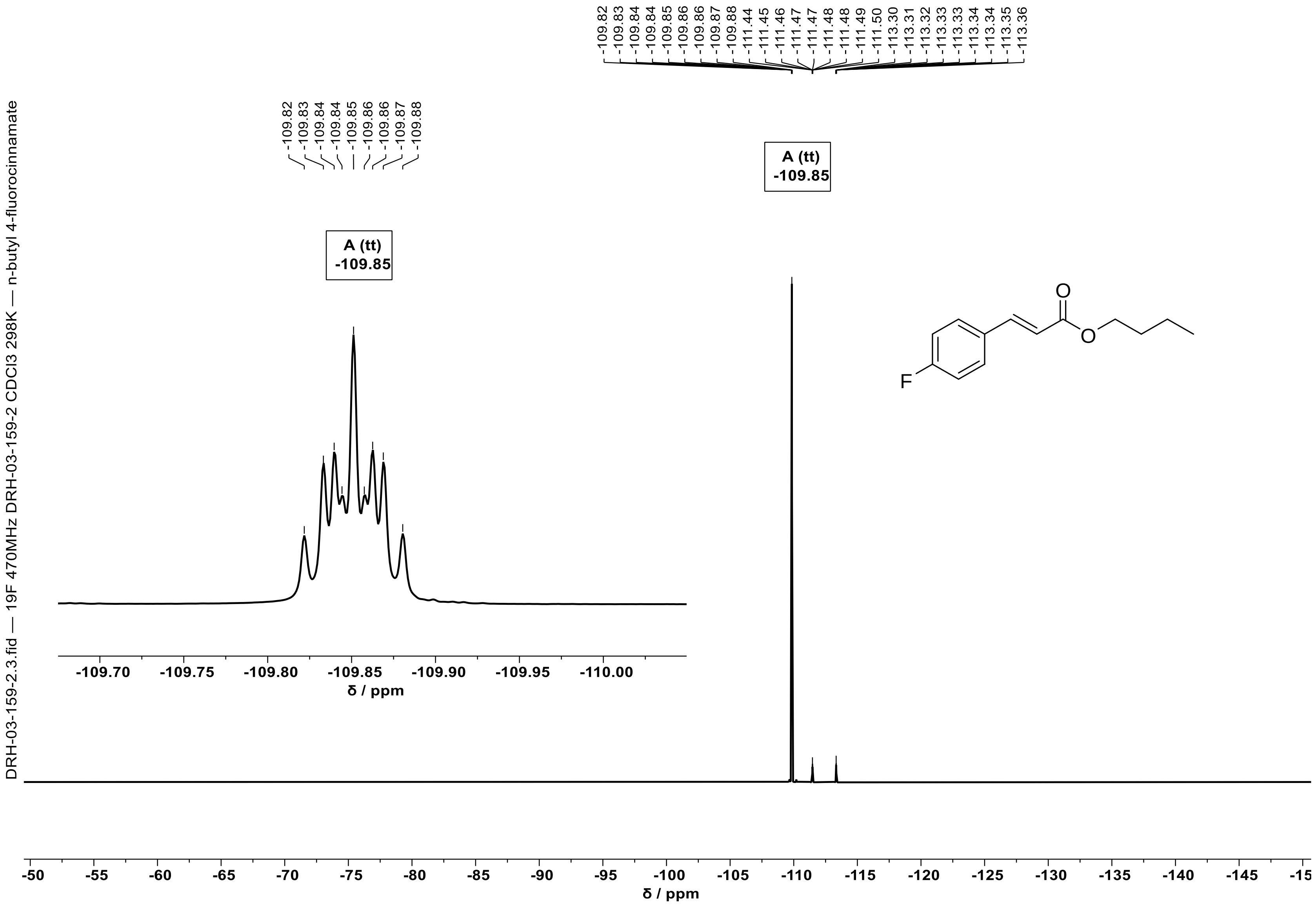

Figure 30: ^{19}F NMR (470 MHz, C*D*Cl$_3$, 64 scans, 298 K) spectrum of n-butyl 4-fluorocinnamate 11. Small impurities are visible at -111.47 and -113.33 ppm (~2%). Lab book ref. DRH-03-159

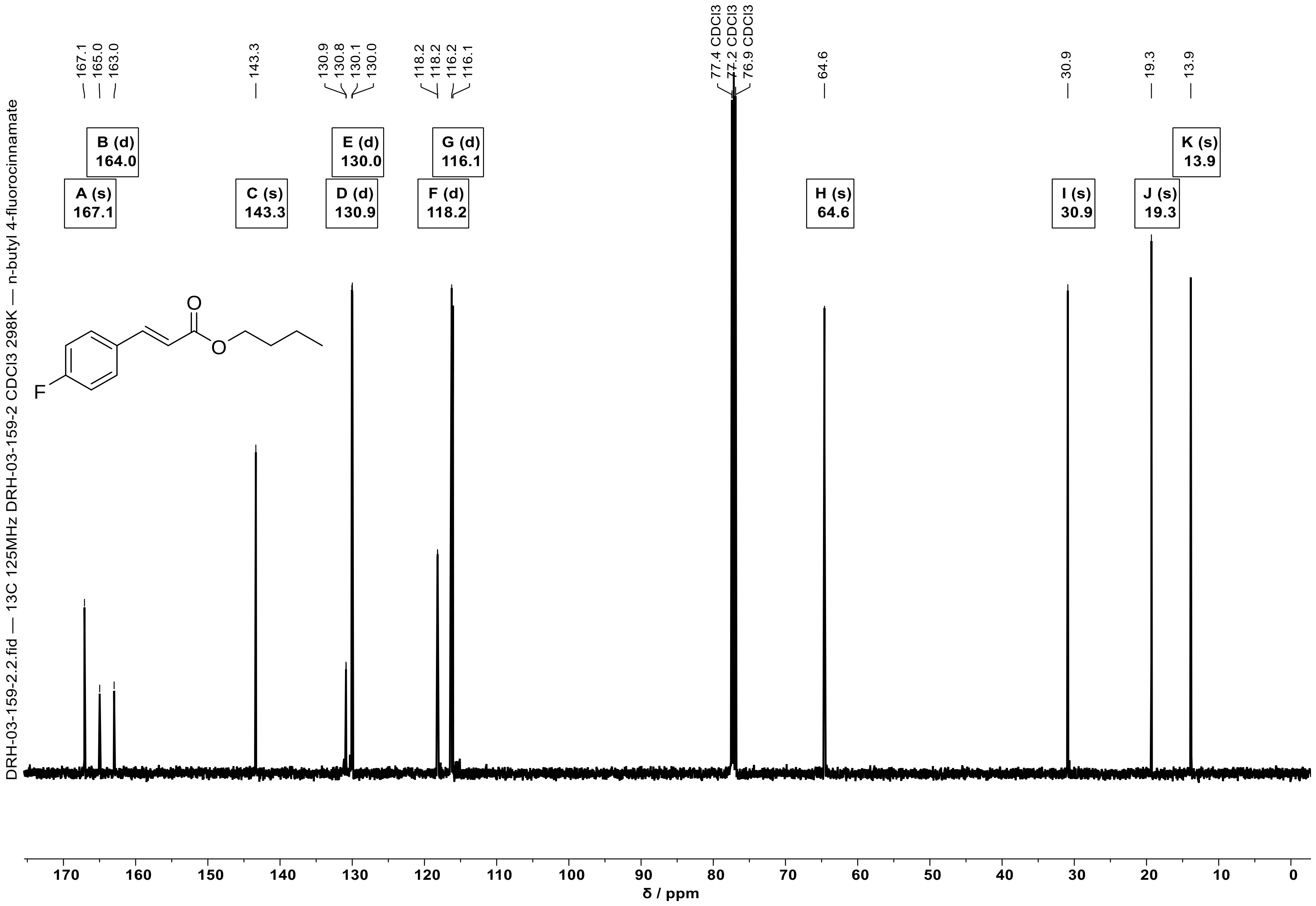

Figure 31: ^{13}C NMR (125 MHz, C*D*Cl$_3$, 128 scans, 298 K) spectrum of n-butyl 4-fluorocinnamate 11. Lab book ref. DRH-03-159

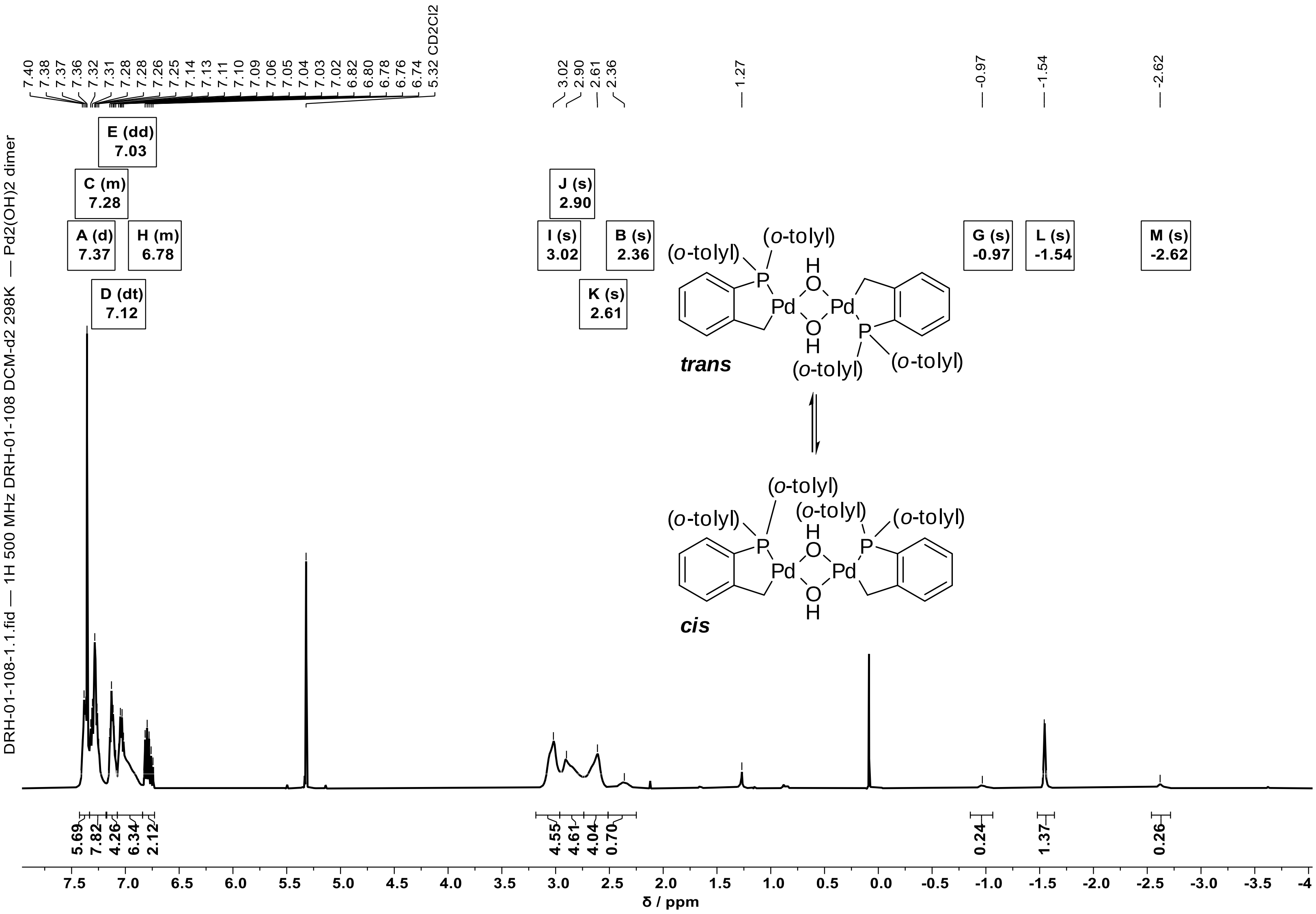

Figure 32: ^{1}**H NMR (500 MHz, DCM-d_2, 32 scans, 298 K) spectrum of the [Pd(C^P)(μ_2-OH)]$_2$ palladacycle 2. Due to poor resolution of broad peaks, this sample was re-ran at 203 K to resolve environments. Lab book ref. DRH-01-108**

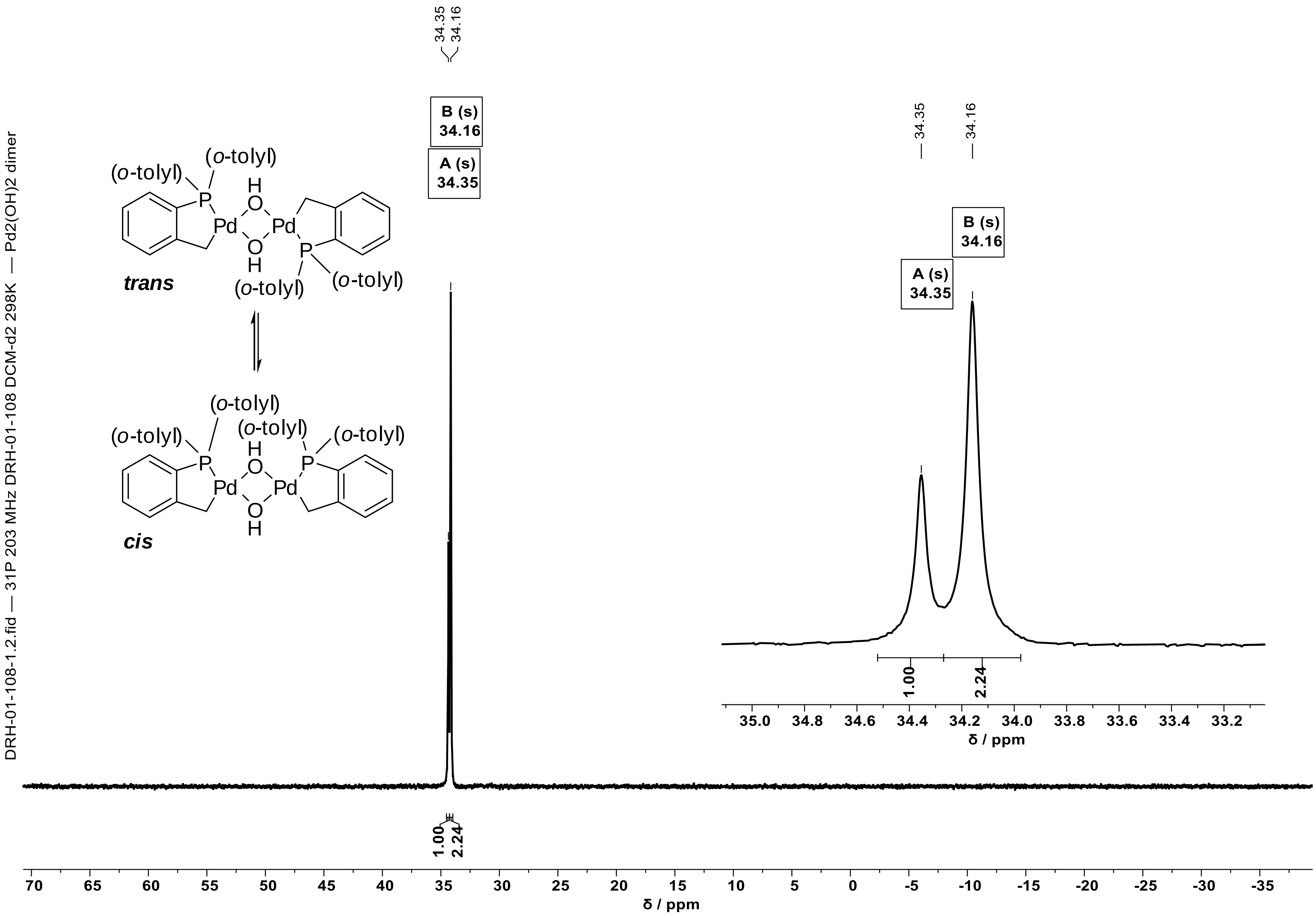

Figure 33: ^{31}P NMR (203 MHz, DCM-d_2, 128 scans, 298 K) spectrum of the [Pd(C^P)(μ_2-OH)]$_2$ palladacycle 2. Cis/trans isomers are present, giving two signals. Due to poor resolution of broad peaks, this sample was re-ran at 203 K to resolve environments. Lab book ref. DRH-01-108

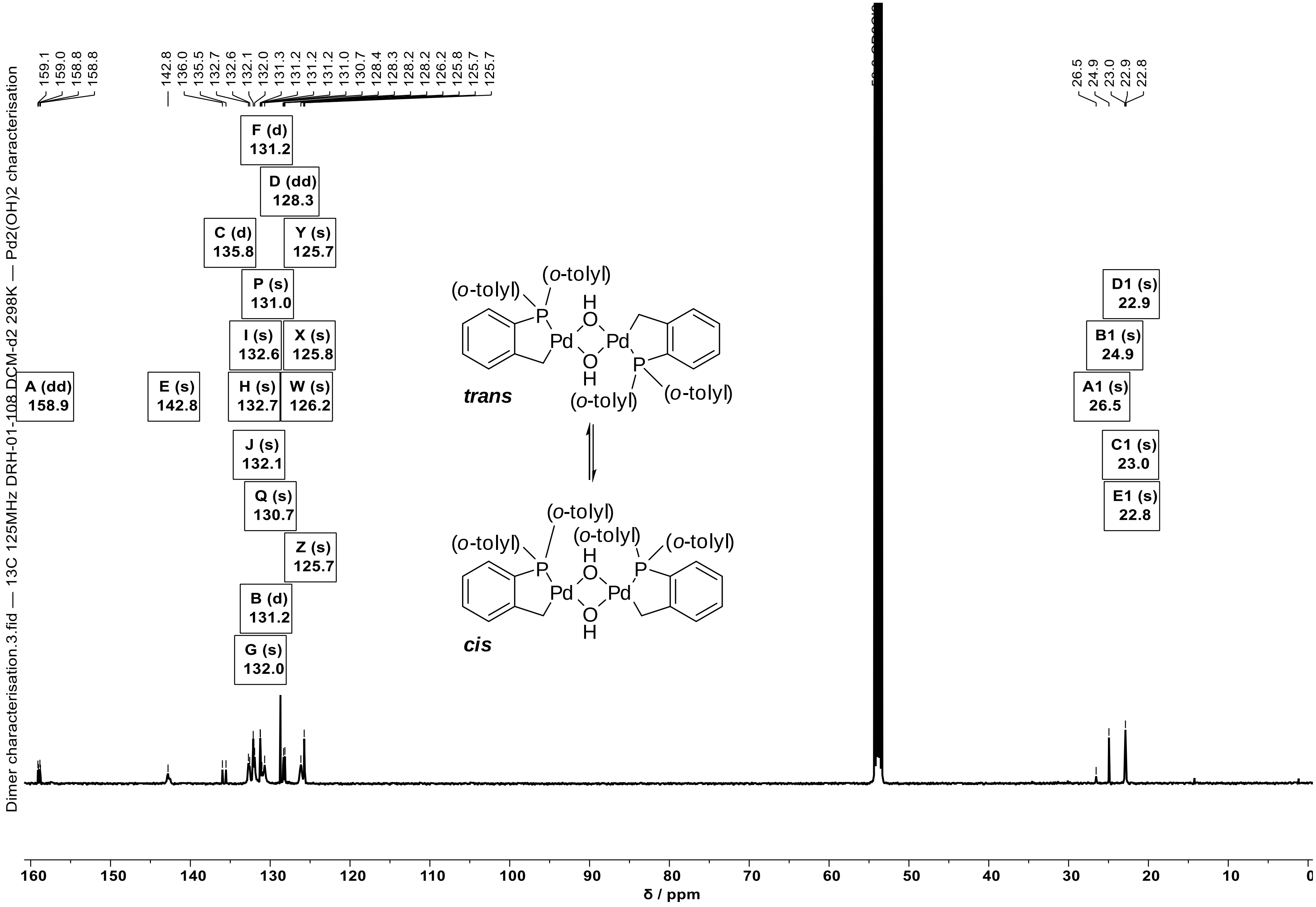

Figure 34: **^{13}C NMR (126 MHz, DCM-*d*$_2$, 49732 scans, 298 K) spectrum of the [Pd(C^P)(μ$_2$-OH)]$_2$ palladacycle 2. Lab book ref. DRH-01-108**

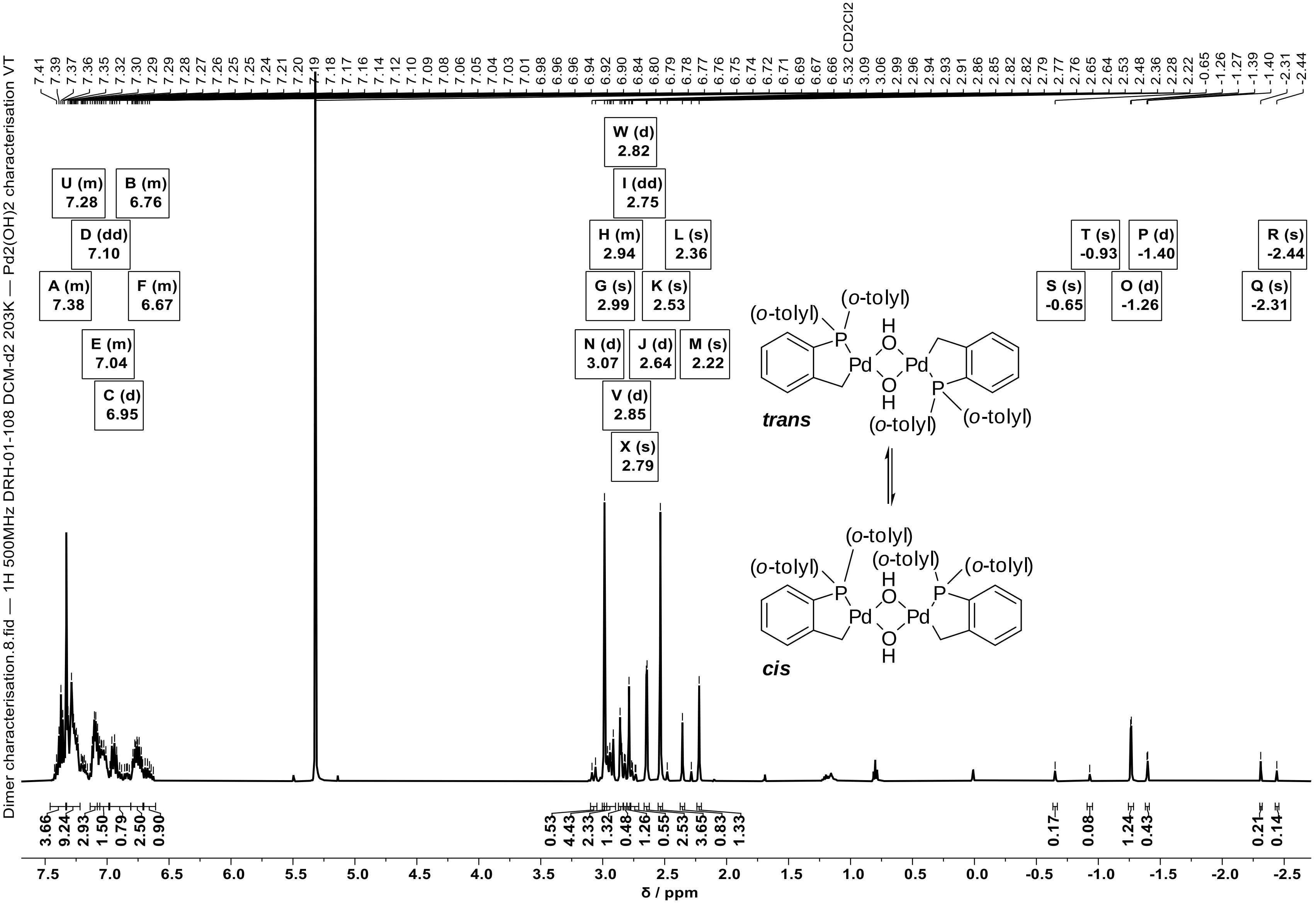

Figure 35: ^{1}H NMR (500 MHz, DCM-d_2, 64 scans, 203 K) spectrum of the [Pd(C^P)(μ_2-OH)]$_2$ palladacycle 2. Lab book ref. DRH-01-108

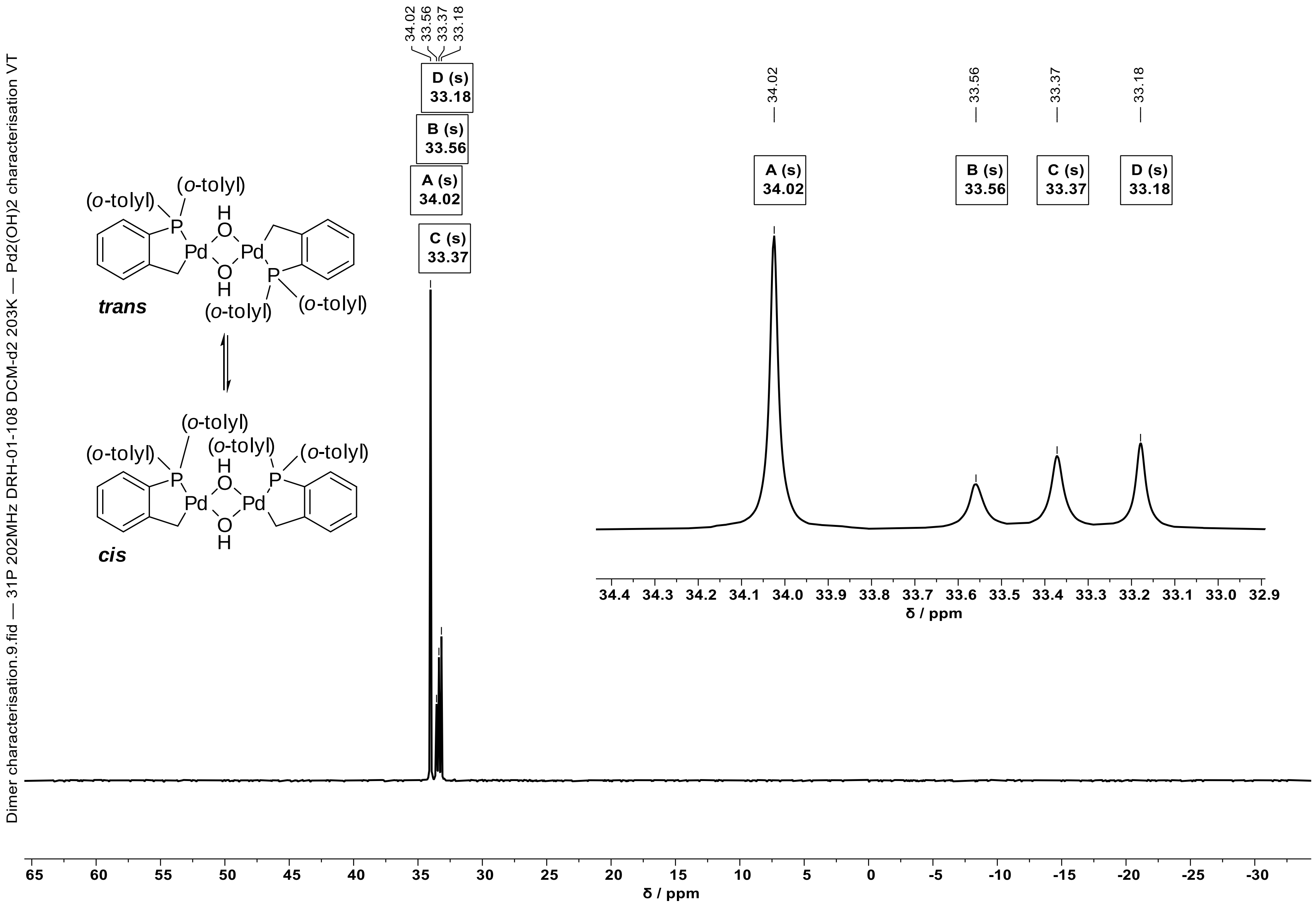

Figure 36: **^{31}P NMR (203 MHz, DCM-*d*$_2$, 256 scans, 203 K) spectrum of the [Pd(C^P)(μ$_2$-OH)]$_2$ palladacycle 2. Lab book ref. DRH-01-108**

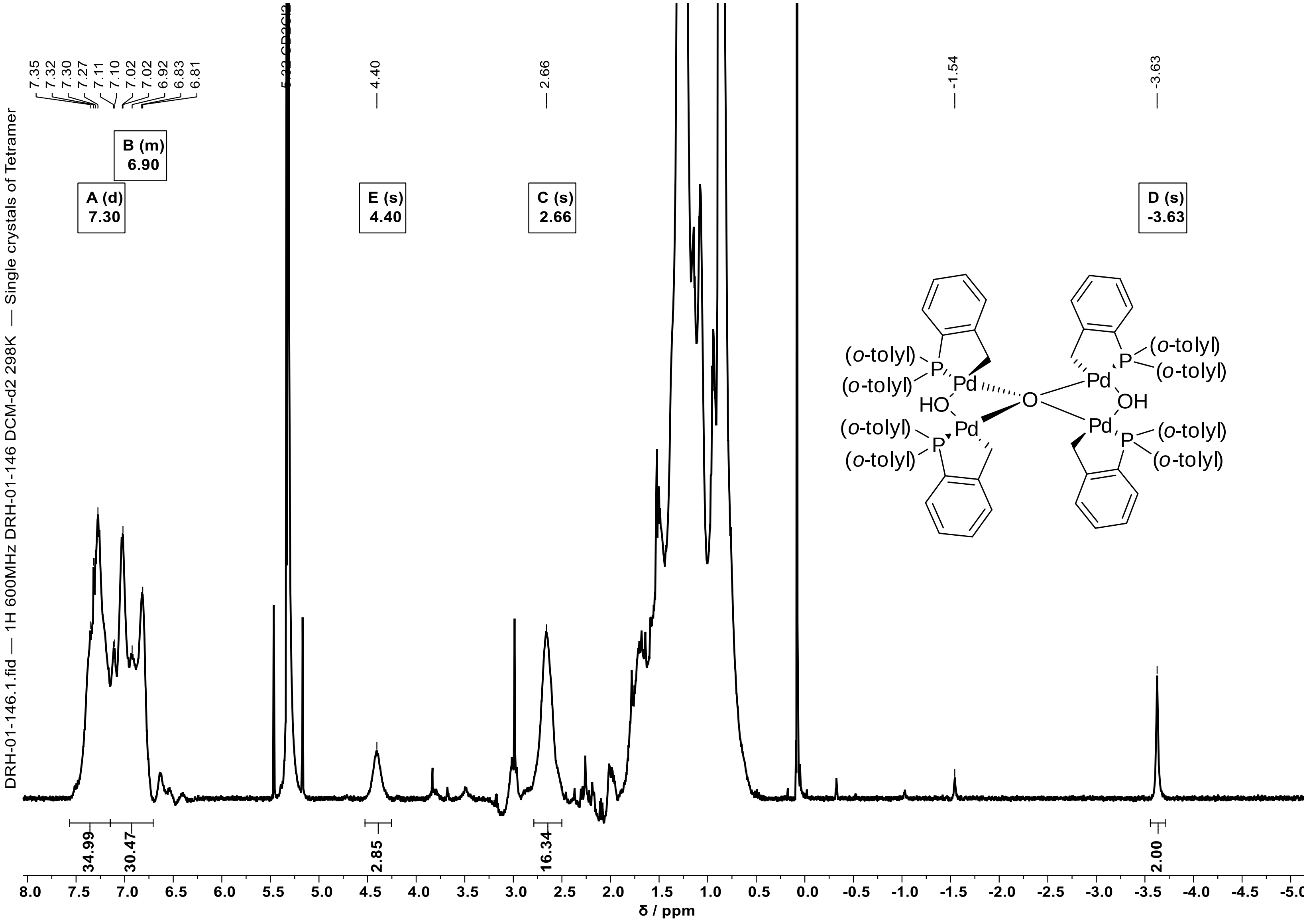

Figure 37: ^{1}H NMR (600 MHz, DCM-d_2, 256 scans, 298 K) spectrum of the palladacyclic $Pd_4(\mu_4\text{-}O)(\mu_2\text{-}OH)_2$ cluster 3. The non-integrated region between 2.0 and 0.5 ppm is residual grease from manual separation of single crystals. Lab book ref. DRH-01-146

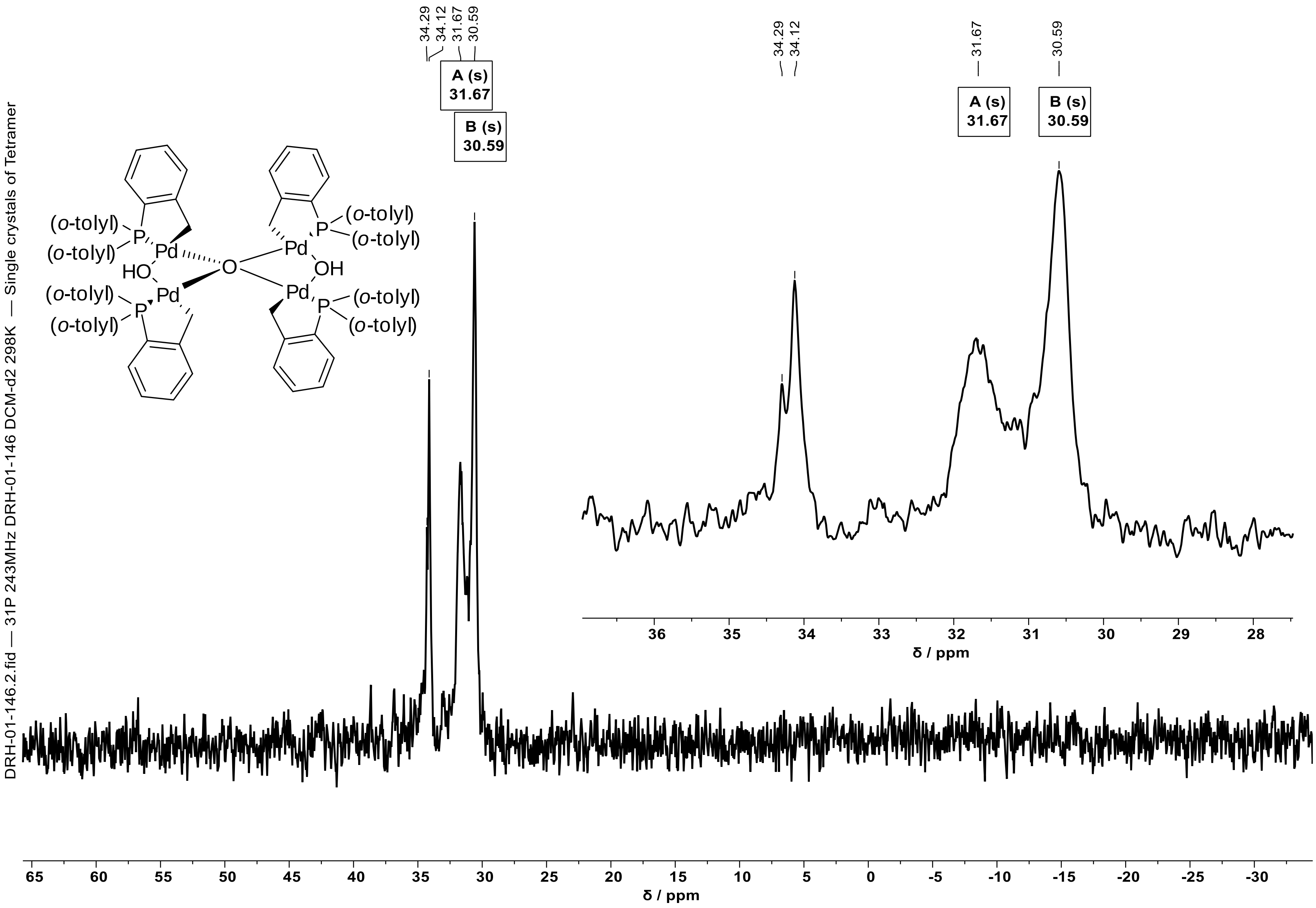

Figure 38: **^{31}P NMR (243 MHz, DCM-d_2, 1024 scans, 298 K) spectrum of the palladacyclic $Pd_4(\mu_4\text{-}O)(\mu_2\text{-}OH)_2$ cluster 3. Lab book ref. DRH-01-146**

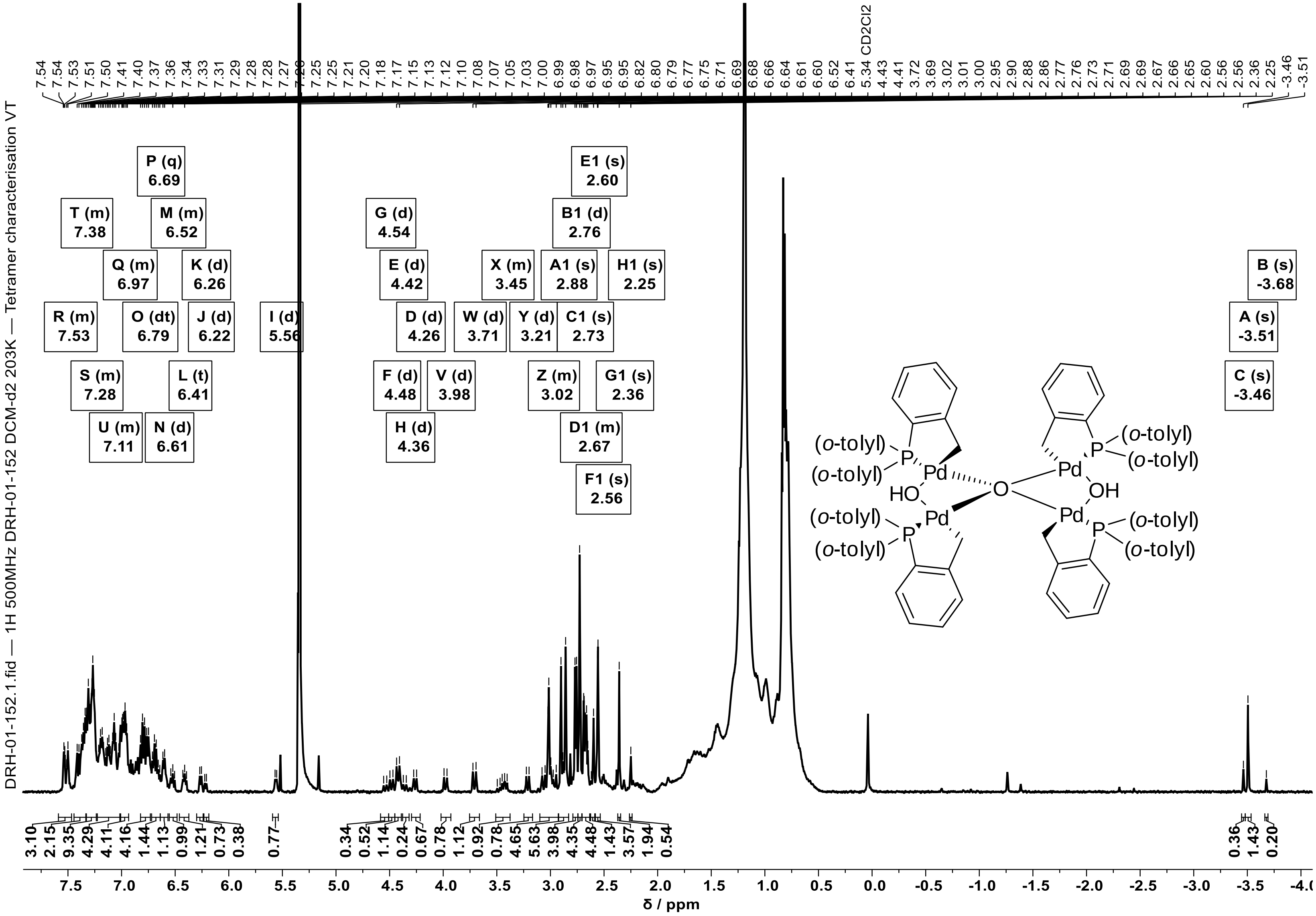

Figure 39: ^{1}**H NMR (500 MHz, DCM-*d*$_2$, 128 scans, 203 K) spectrum of the palladacyclic Pd$_4$(μ$_4$-O)(μ$_2$-OH)$_2$ cluster 3. The non-integrated region between 2.0 and 0.5 ppm is residual grease from manual separation of single crystals, Lab book ref. DRH-01-152**

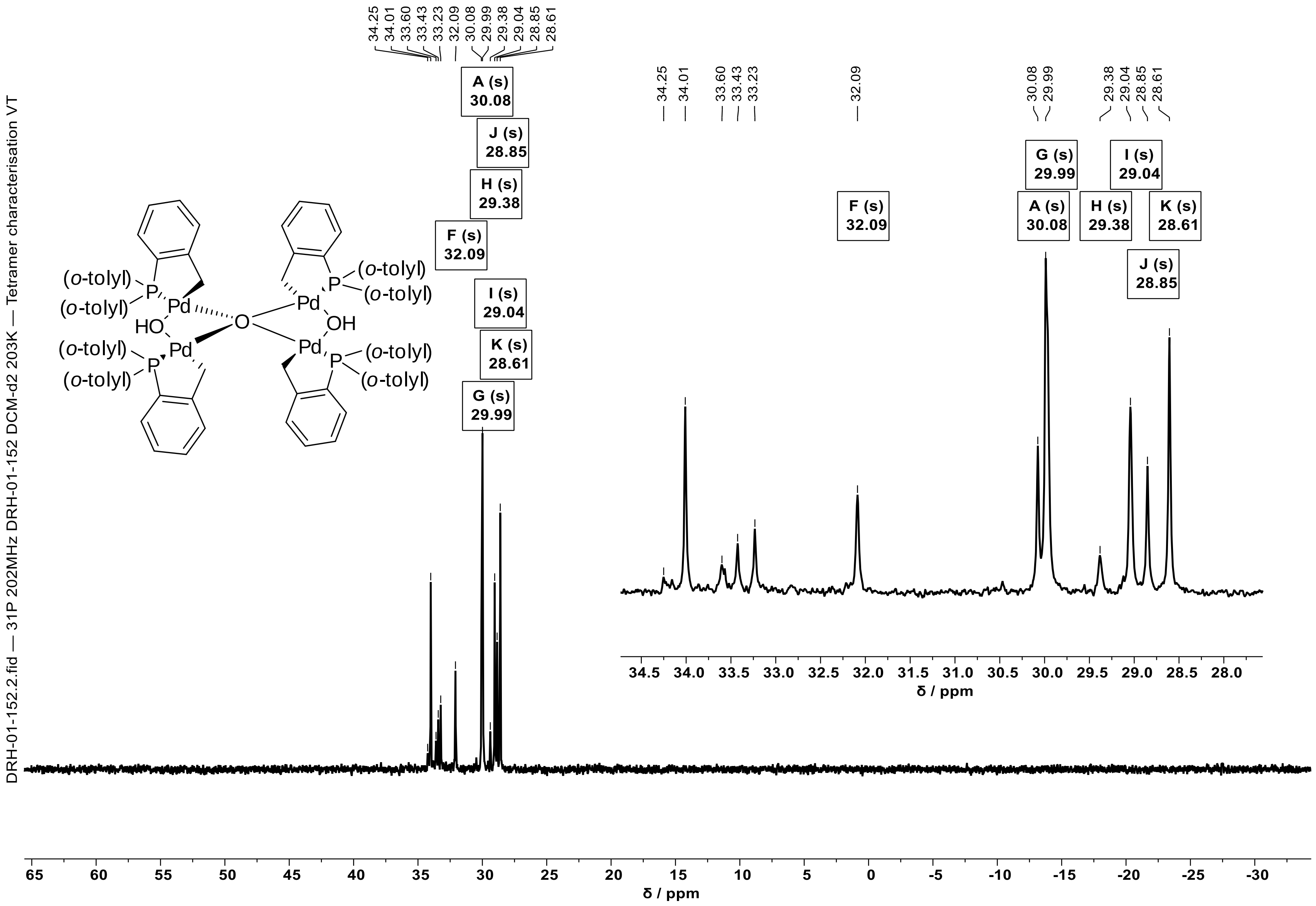

Figure 40: ^{31}P NMR (203 MHz, DCM-d_2, 512 scans, 203 K) spectrum of the palladacyclic $Pd_4(\mu_4\text{-}O)(\mu_2\text{-}OH)_2$ cluster 3. Lab book ref. DRH-01-152

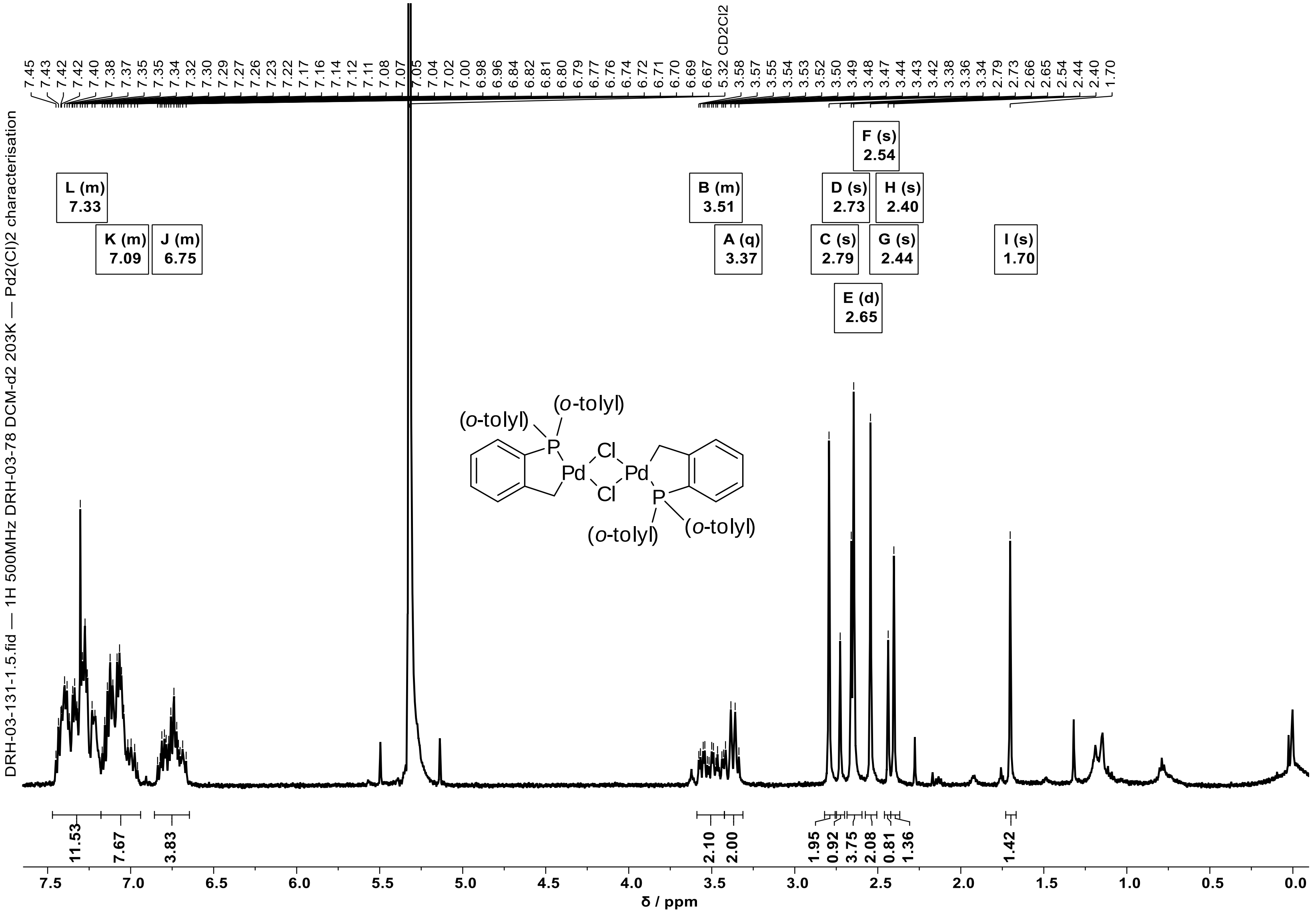

Figure 41: ^{1}H NMR (500 MHz, DCM-d_2, 128 scans, 203 K) spectrum of the [Pd(C^P)(μ_2-Cl)]$_2$ palladacycle 4. Residual solvent signals (most likely water and grease) below 1.5 ppm have not been integrated. Lab book ref. DRH-03-78

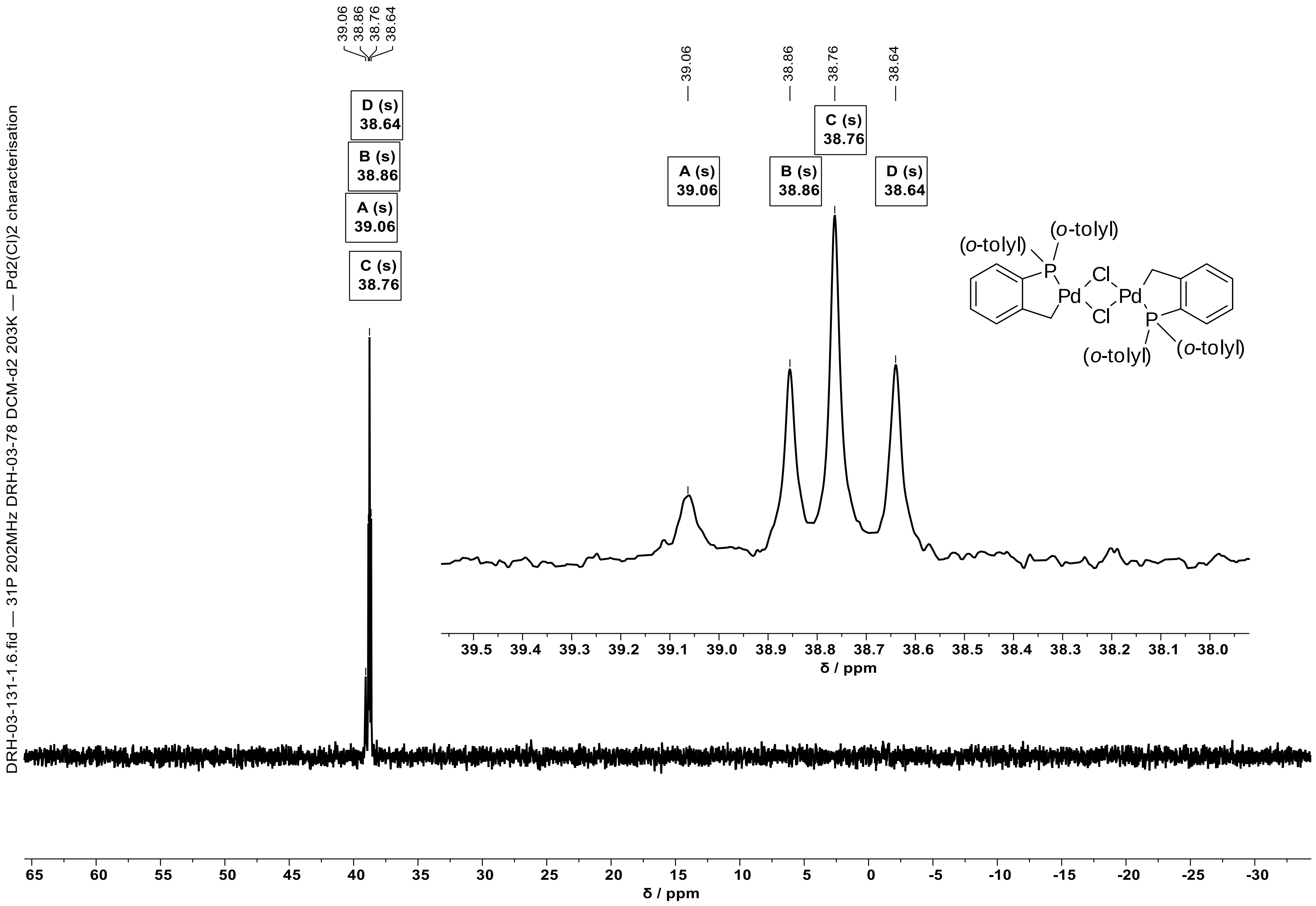

Figure 42: ^{31}P NMR (203 MHz, DCM-d_2, 128 scans, 203 K) spectrum of the $[Pd(C\text{^}P)(\mu_2\text{-}Cl)]_2$ palladacycle 4. Lab book ref. DRH-03-78

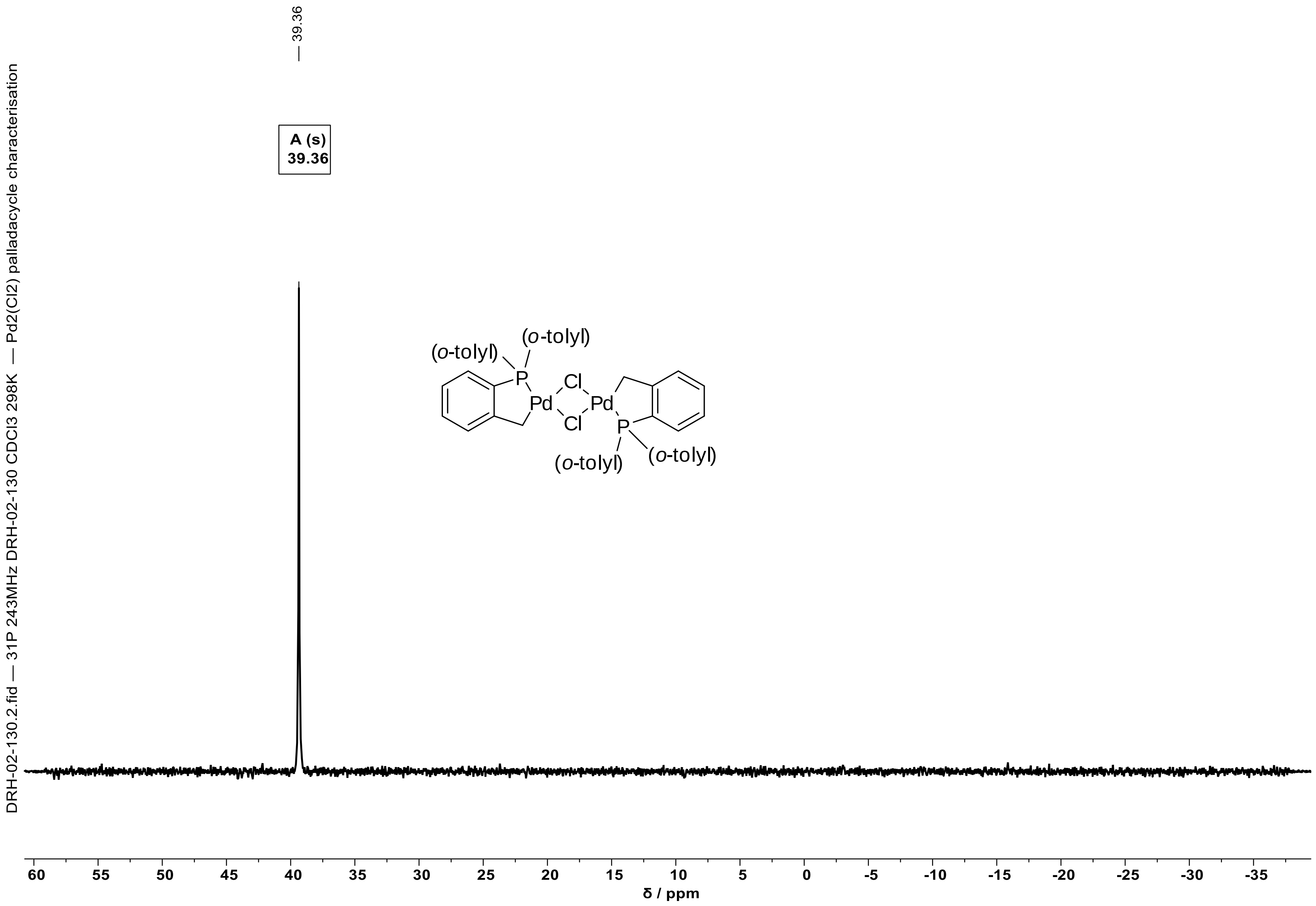

Figure 43: ^{31}P NMR (243 MHz, C*D*Cl$_3$, 128 scans, 298 K) spectrum of the [Pd(C^P)(μ$_2$-Cl)]$_2$ palladacycle 4. Lab book ref. DRH-02-130

6. Density Functional Theory (DFT) Calculations

Unless stated otherwise, all calculations were performed at the DFT level using the B3LYP functional[8–10] in the Gaussian16(revision A.03) suite of programs.[11] Geometry optimization and frequency calculations were performed using the def2-SVP basis set in the gas phase for all structures with a superfine integration grid for all atoms. The SMD continuum model[12] (solvent = tetrahydrofuran, ε=7.4257) was used for all calculations, alongside the D3 version of Grimme's dispersion corrections with Becke-Johnson damping.[13] Unless stated otherwise, all calculations were carried out at 298.15 K and 1.00 Atm pressure. The nature of stationary points was verified by frequency calculations, with minima having zero imaginary frequencies. All energies are displayed in Hartrees.

6.1 DFT Calculations – Hexamer Variant Optimised Structure Coordinates

All calculations in this section were done at the def2-SVP level of theory due to the size of the molecule. All optimised structures follow the basic structure shown below, but with varying Z and X groups. The obtained crystal structure for **7** was used as a starting point for all structures. For visual clarity, most H atoms are not shown.

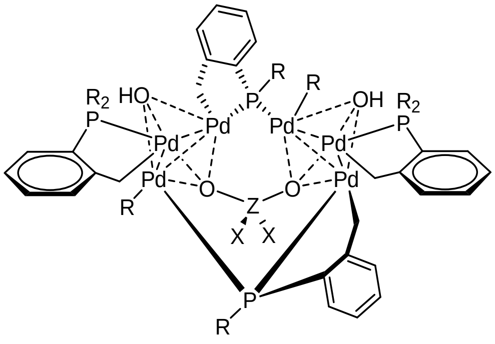

R = *o*-tolyl

Hexamer Z = P, X = Me (7a)

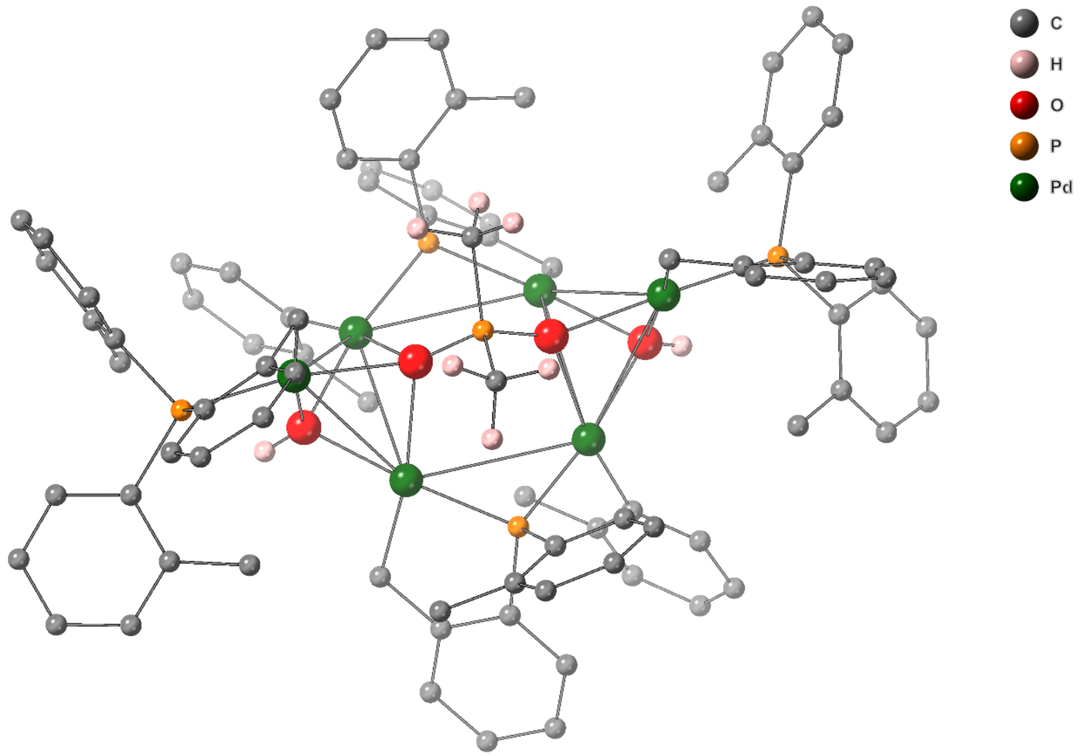

Sum of electronic and zero-point Energies= -6101.973152

Sum of electronic and thermal Energies= -6101.868310

Sum of electronic and thermal Enthalpies= -6101.867366

Sum of electronic and thermal Free Energies= -6102.126534

0 2
C 5.96471132 21.01527388 15.66929326
H 5.68794728 20.31541881 14.85972118
H 7.02749990 20.85712296 15.90510872
C 5.10520857 20.78021662 16.88399143
C 5.42597639 19.86789541 17.89979479
H 6.35283188 19.29149597 17.83571327
C 4.57305979 19.70246153 18.99630365
H 4.84163638 18.99673308 19.78657325
C 3.38611218 20.43895598 19.09815233
H 2.73266683 20.31101156 19.96419953
C 3.05227712 21.35564507 18.09760087
H 2.14479808 21.95936652 18.17627360
C 3.91501556 21.51943194 17.00995403
C 2.47559667 22.06594879 14.50652537
C 1.70219274 20.96494504 14.91046515
H 1.82779938 20.54892611 15.91033408
C 0.77285955 20.38585156 14.04496496
H 0.17929203 19.53138938 14.37795514
C 0.61568427 20.90314799 12.75845453
H -0.10486226 20.45732500 12.06838422
C 1.38604476 21.99380728 12.34965039
H 1.26324567 22.39490665 11.34039397
C 2.32548297 22.59584022 13.19991488
C 3.13660716 23.75751711 12.69957875
H 2.85591342 24.01379093 11.66906950
H 4.21200557 23.52537065 12.71345975
H 2.99756016 24.65498542 13.32277026
C 3.06779787 24.27439432 16.38315607
C 1.84012388 24.78350781 15.92966852
H 1.26587720 24.22951665 15.18531791
C 1.34344012 25.99226443 16.41915203
H 0.38762849 26.37556487 16.05481270
C 2.07801394 26.70145916 17.37099918
H 1.70550494 27.65328906 17.75736975
C 3.29281711 26.19222914 17.83546207

H 3.85887031 26.74870717 18.58645709
C 3.81430894 24.97829113 17.36471261
C 5.11880147 24.46892861 17.91339385
H 5.55234588 25.18577280 18.62324840
H 5.84889300 24.29287938 17.10583455
H 4.98985942 23.50878115 18.43578895
C 8.54355922 20.77851579 12.99603617
H 7.85303350 21.06846270 12.19534585
H 8.02004766 20.12666944 13.71025554
H 9.40074530 20.24353839 12.56598657
C 9.84488434 21.69402752 15.42172181
H 10.55968205 20.89034836 15.19338696
H 9.07432686 21.32393116 16.11052824
H 10.38660918 22.52228009 15.89052295
C 5.96871539 25.59138922 10.97966516
H 4.91844308 25.25834962 11.04532738
H 6.03765285 26.58124374 11.46467093
C 6.40142331 25.65482996 9.53771981
C 5.85202112 26.57600742 8.62969430
H 5.11972713 27.30873672 8.98158813
C 6.22337533 26.54881803 7.28368784
H 5.78844148 27.26958205 6.58610707
C 7.14345925 25.59703396 6.82098790
H 7.42928355 25.57586221 5.76656508
C 7.71132555 24.69032171 7.71530332
H 8.44819016 23.96300764 7.37067034
C 7.35103004 24.73464057 9.06920834
C 7.89448650 21.97247551 9.82789966
C 9.05679654 21.20845511 9.62160425
H 10.03109404 21.67695086 9.78204740
C 8.98419289 19.86457530 9.25263636
H 9.90174973 19.29037952 9.10300922
C 7.73287725 19.26658839 9.08938481
H 7.65659867 18.21348090 8.80653010
C 6.57164154 20.01678213 9.29431934
H 5.59454739 19.54139734 9.17130119
C 6.62096218 21.37056359 9.66251505
C 5.34081262 22.12307104 9.90397460
H 5.23137142 22.97163825 9.21047253
H 5.32619621 22.54951901 10.92013092
H 4.46715903 21.46651358 9.78661579
C 7.55939485 27.27887001 15.45365427
C 6.91379029 27.54928398 16.66764827
H 6.67778703 26.73327144 17.35233626
C 6.57440850 28.86089133 17.02515994
H 6.07086883 29.05273920 17.97699095
C 6.89192067 29.91452054 16.16632209
H 6.64330309 30.94414098 16.43690556
C 7.53276570 29.64591371 14.95217072
H 7.78035238 30.47165650 14.27817475
C 7.87436104 28.33785121 14.57269422
C 8.56340766 28.07979518 13.25834214
H 9.51874711 27.55347067 13.40408268
H 8.76430911 29.01526091 12.71553783
H 7.95604431 27.42796369 12.61155334
C 12.27503455 26.66406188 14.12020936
H 13.28430442 26.31013110 14.40020763
H 12.35694992 27.14773449 13.13097425
C 11.76261555 27.63155199 15.15667360
C 12.28796854 28.92773044 15.29959940
H 13.06085848 29.27430111 14.60719140
C 11.84287391 29.76436888 16.32504854
H 12.26002894 30.77010670 16.42420604
C 10.87114789 29.31915560 17.23311226
H 10.52795512 29.97500645 18.03692906
C 10.32657111 28.04348965 17.09197815
H 9.54980723 27.69626513 17.77553937
C 10.75746935 27.21645826 16.04549727
C 10.16789223 24.60206061 17.19595989
C 8.97550365 24.18810398 17.81490751
H 8.02512771 24.49948886 17.37807761
C 8.98737897 23.37284790 18.94720923
H 8.04503811 23.05964498 19.40393513
C 10.21187004 22.95991089 19.47756528
H 10.24311552 22.31535786 20.35982901
C 11.40354079 23.37052322 18.87414436
H 12.35942768 23.04021412 19.28963840
C 11.41264047 24.19079717 17.73469397
C 12.71941623 24.56634416 17.09405040
H 12.74467290 24.22900991 16.04563264
H 13.56700350 24.11577344 17.62883762
H 12.86281008 25.65812599 17.07384393
C 10.60669458 24.93484991 9.25103095
C 11.13532482 24.17521708 8.19720160
H 11.28391943 23.09977566 8.32030686
C 11.47144254 24.77338772 6.97563418
H 11.88135419 24.16320015 6.16564622
C 11.27244126 26.14424996 6.79893936
H 11.52273956 26.62260820 5.84824920
C 10.74626698 26.90511101 7.84811723
H 10.58746862 27.97902411 7.70982814
C 10.40658165 26.32308276 9.07989370

C 9.82411842 27.16293701 10.18653277
H 8.84491043 26.77193689 10.50093221
H 9.69302640 28.21021012 9.87539427
H 10.46425874 27.14577712 11.08263697
C 12.41004000 20.10278725 12.98538553
H 11.50178789 19.73682265 13.49200993
H 12.44318194 19.63688335 11.98265274
C 13.63220703 19.77693482 13.76071042
C 13.64803424 18.91752355 14.87788927
H 12.71013848 18.45672304 15.20312012
C 14.83110539 18.63765949 15.56682661
H 14.80755267 17.96731474 16.43095850
C 16.04638772 19.20088098 15.15405486
H 16.97215453 18.97639770 15.68974903
C 16.05796649 20.07480488 14.06202839
H 16.99253971 20.54997303 13.75289235
C 14.86976888 20.37980815 13.38863738
C 16.17707640 22.76802143 12.38414672
C 17.23062079 22.78594572 11.45383830
H 17.20791575 22.10186570 10.60334118
C 18.30851949 23.66137708 11.59978671
H 19.11822450 23.65593127 10.86553777
C 18.34459261 24.53564103 12.68858541
H 19.18020582 25.22903891 12.81358215
C 17.30418452 24.52144775 13.62173598
H 17.33452029 25.20497638 14.47492412
C 16.21077388 23.65154471 13.49592536
C 15.12076961 23.66885510 14.53416726
H 15.26233500 24.50133865 15.23748785
H 15.10048799 22.73248924 15.11542926
H 14.12447485 23.76220705 14.07106578
C 14.94851712 20.92471128 10.49909200
C 15.26632039 19.56160339 10.39972605
H 15.44855809 18.98708845 11.31037252
C 15.34979101 18.93098249 9.15542507
H 15.59852597 17.86823571 9.09814185
C 15.11426470 19.66733025 7.99298289
H 15.17466213 19.18675841 7.01307882
C 14.79735299 21.02626409 8.08405700
H 14.61100533 21.60048353 7.17222739
C 14.70299333 21.67796762 9.32192588
C 14.34081547 23.13744070 9.37974379
H 15.13746166 23.73561996 9.85043245
H 13.43147295 23.29053983 9.98189363
H 14.16040159 23.54283715 8.37504199
O 5.94413876 24.94016275 14.06382320
H 5.22789422 25.58701362 14.09581461
O 7.86838007 23.17823720 14.11338652
O 10.20199437 23.01948751 13.06959673
O 12.15951971 24.39767076 11.96259343
H 12.83930556 24.94092873 11.54434110
P 3.73099534 22.73144396 15.66487177
P 9.11784846 22.24089665 13.87064271
P 8.09852126 23.71479795 10.38728467
P 10.04491223 25.58476504 15.63940993
P 14.69877557 21.70212330 12.14337398
Pd 5.81045139 22.90004655 14.88175903
Pd 6.94697855 24.32922174 12.22549345
Pd 7.90579353 25.40724639 14.92172202
Pd 10.24066411 24.06517012 10.99223132
Pd 11.23028329 24.95126637 13.83069072
Pd 12.44155965 22.18747107 12.48517327

Hexamer Z = S, X = Me (7b)

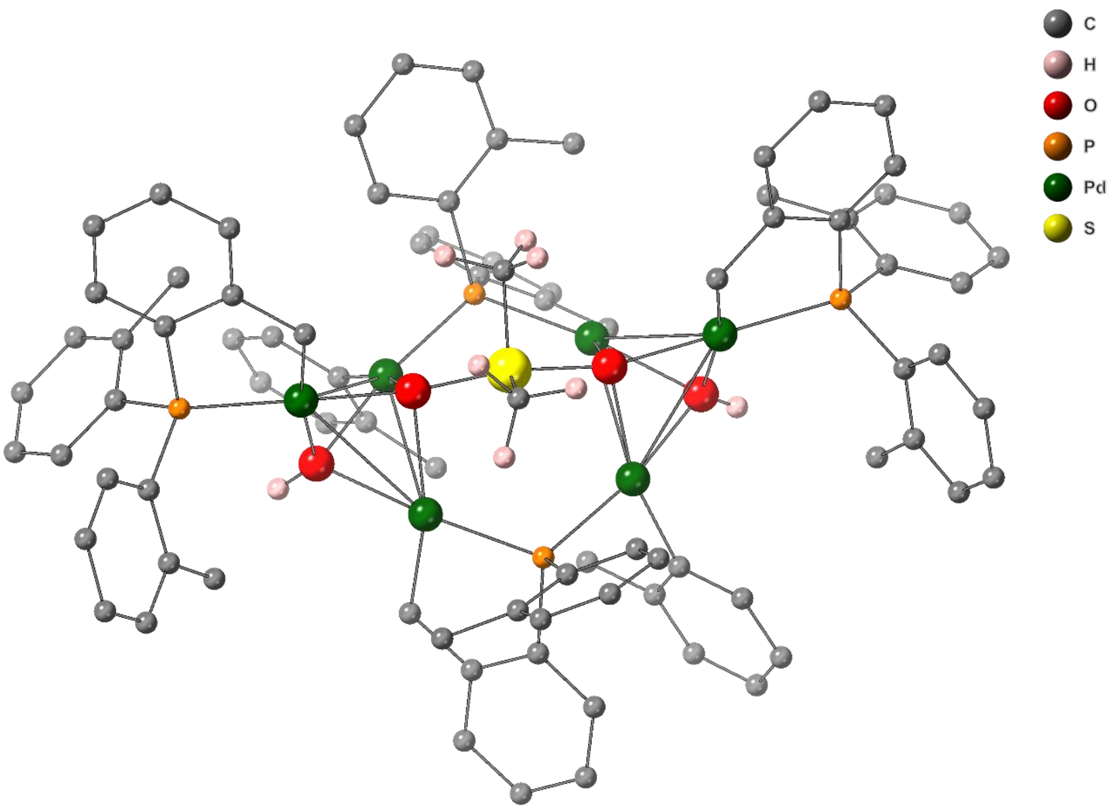

Sum of electronic and zero-point Energies= -6158.640236

Sum of electronic and thermal Energies= -6158.535968

Sum of electronic and thermal Enthalpies= -6158.535024

Sum of electronic and thermal Free Energies= -6158.789732

0 1
C 5.67821300 21.03816500 15.78842500
H 5.43314700 20.31211300 14.99087500
H 6.73649200 20.89333400 16.05617300
C 4.78646800 20.82076100 16.98438500
C 5.07978000 19.89743000 18.00074200
H 5.99569000 19.30313400 17.93931400
C 4.21980400 19.74294300 19.09209100
H 4.46949000 19.02705100 19.87961700
C 3.04923900 20.50582500 19.19230100
H 2.38865900 20.38961000 20.05474200
C 2.74250300 21.43288600 18.19368400
H 1.84959000 22.05781500 18.27429700
C 3.60898600 21.58333100 17.10491700
C 2.09413500 22.27747400 14.67369600
C 1.24883300 21.23500100 15.08762100
H 1.38117100 20.78698000 16.07265400
C 0.23863600 20.75346900 14.25306300
H -0.40894100 19.94285700 14.59535500
C 0.06878200 21.31281500 12.98597400
H -0.71633900 20.94527400 12.32049100
C 0.91028400 22.34483900 12.56523900
H 0.77798100 22.77837200 11.57057000
C 1.93400600 22.84547800 13.38379300
C 2.82113400 23.94450700 12.86660200
H 2.49197100 24.27517200 11.87164900
H 3.86676800 23.60846900 12.79039300
H 2.81804600 24.82117500 13.53283300

C 2.88382400 24.37664700 16.58316700
C 1.67321800 24.96944000 16.19042300
H 1.04526200 24.47517100 15.44731800
C 1.25994000 26.18577200 16.73685200
H 0.31606600 26.63361100 16.41723900
C 2.06157400 26.81954300 17.68755000
H 1.75456600 27.77532500 18.11934100
C 3.26128500 26.22866100 18.09075600
H 3.88186100 26.72598200 18.84056700
C 3.70010300 25.00675600 17.55951800
C 4.99323400 24.41094900 18.04712000
H 5.51485600 25.10451700 18.72020300
H 5.66461800 24.16632900 17.20682700
H 4.82148400 23.47108200 18.59437900
C 8.58510800 20.96036100 13.01773100
H 7.88551700 21.14182000 12.19901900
H 8.08080300 20.39837900 13.81402500
H 9.47733400 20.43655200 12.65739700
C 9.70533100 21.90250200 15.28317900
H 10.10583700 20.89632700 15.11366100
H 8.86705900 21.87890100 15.98735800
H 10.50024400 22.55771500 15.64531700
C 6.11139300 25.48086300 10.86948800
H 5.02866000 25.25619400 10.89157900
H 6.25047800 26.47866800 11.32378600
C 6.61650700 25.45214700 9.45114500
C 6.09608000 26.29850600 8.45602500
H 5.33464100 27.03746100 8.72329900
C 6.52818700 26.18746800 7.13332400
H 6.11458100 26.85029400 6.36839400
C 7.47747600 25.21750300 6.78044700
H 7.80659800 25.12089300 5.74290200
C 8.01380900 24.38547000 7.76178000
H 8.76272900 23.63812200 7.49810100
C 7.60087900 24.52013100 9.09482400
C 8.06294900 21.81485100 9.87599700
C 9.22187900 21.05354300 9.64416500
H 10.19531800 21.50789600 9.84607900
C 9.14998000 19.73616700 9.18886700
H 10.06715800 19.16677600 9.01846500
C 7.89917200 19.15842700 8.96182500
H 7.82159900 18.12619200 8.61050400
C 6.74047500 19.90554900 9.18769300
H 5.76276200 19.44865700 9.01074300
C 6.79039400 21.23435500 9.63972200
C 5.50557500 21.98139000 9.87183600
H 5.41481200 22.84581400 9.19526600
H 5.46224400 22.38805600 10.89689900
H 4.63568800 21.32822900 9.71358300
C 7.45421700 27.17862300 15.39845600
C 6.79100300 27.42038200 16.61150700
H 6.52842800 26.58474000 17.26230800
C 6.47055400 28.72296200 17.01406500
H 5.95660600 28.88840700 17.96529900
C 6.82163200 29.80259600 16.20096100
H 6.59065000 30.82625000 16.50774000
C 7.47389200 29.56565400 14.98708100
H 7.74780200 30.41073400 14.34789900
C 7.79419200 28.26563900 14.56322500
C 8.47361400 28.04809200 13.23809400
H 9.26877600 27.29448000 13.31155200
H 8.90710300 28.97969500 12.84506100
H 7.76034700 27.66475700 12.49067900
C 12.13402300 26.66176500 13.94890000
H 13.20025400 26.43714400 14.14168100
H 12.06880900 27.12326300 12.94716400
C 11.60338400 27.58903200 15.00989400
C 12.09828300 28.89382100 15.18305400
H 12.86312800 29.27439400 14.49943900
C 11.63376900 29.69489300 16.22771800
H 12.02675800 30.70757700 16.35219000
C 10.67503100 29.20156400 17.12477400
H 10.31780100 29.82635700 17.94702100
C 10.16319800 27.91664800 16.95209000
H 9.40180700 27.53174100 17.63200600
C 10.61238100 27.12527200 15.88559900
C 10.11437900 24.57680800 17.09032600
C 8.93555600 24.19100700 17.75156700
H 7.97490600 24.42570400 17.28818300
C 8.97053200 23.50265200 18.96506700
H 8.03677400 23.21288100 19.45391800
C 10.20547500 23.18666400 19.53560700
H 10.25520100 22.64298900 20.48262500
C 11.38407800 23.56681800 18.88848800
H 12.34945200 23.31567900 19.33674900
C 11.36938600 24.26386400 17.66937300
C 12.66924700 24.63122900 17.00934100
H 12.70605900 24.24885000 15.97655600
H 13.52447900 24.22616000 17.56813600
H 12.79027700 25.72382600 16.93677000
C 10.73926100 24.82161600 9.36862400
C 11.31506000 24.14506700 8.28221700

H 11.52983600 23.07582500 8.35883400
C 11.60874700 24.81038200 7.08507500
H 12.05271200 24.26058900 6.24994200
C 11.31854300 26.17111900 6.96281200
H 11.53028900 26.70184900 6.03061500
C 10.74783600 26.85242000 8.04289300
H 10.51592400 27.91774900 7.94699500
C 10.45374300 26.19966800 9.25094600
C 9.83870500 26.96066800 10.39482900
H 9.00025700 26.40531400 10.83666000
H 9.47496100 27.94952400 10.07785200
H 10.56560500 27.11059500 11.20914600
C 12.69554200 20.27019600 13.25899000
H 11.94825200 20.24778100 14.06596800
H 12.30929100 19.64171300 12.43432000
C 14.01830700 19.73121700 13.73949000
C 14.13072900 18.70572600 14.69240800
H 13.22476600 18.27847300 15.13149100
C 15.38640900 18.23513600 15.08828900
H 15.45448800 17.44303900 15.83879400
C 16.55676600 18.77490100 14.53964300
H 17.53488300 18.40872400 14.86064400
C 16.46627000 19.79824400 13.59309000
H 17.37023500 20.24412800 13.17083900
C 15.20609100 20.26792000 13.20651600
C 16.25719200 22.84551900 12.34953200
C 17.33554100 22.90304200 11.45171900
H 17.35184500 22.24091700 10.58429900
C 18.38812600 23.79837000 11.64858100
H 19.21762000 23.82922000 10.93816800
C 18.36754300 24.64863300 12.75549800
H 19.18117000 25.35935400 12.92067800
C 17.30412100 24.58729900 13.65860900
H 17.29644100 25.24797800 14.52953300
C 16.23608300 23.69288000 13.48580800
C 15.13169600 23.65844600 14.50721000
H 15.31998400 24.38862800 15.30611500
H 15.03583300 22.66295100 14.96941700
H 14.15458900 23.88711400 14.05359600
C 15.15445500 20.99757400 10.36258700
C 15.49844800 19.64904600 10.18174900
H 15.67906100 19.01486700 11.05089300
C 15.60965300 19.10365100 8.90048500
H 15.87891600 18.05176700 8.77859400
C 15.37304500 19.90971500 7.78617600
H 15.45412100 19.49553900 6.77806000
C 15.02876700 21.25287500 7.96063200
H 14.84213100 21.88187900 7.08621200
C 14.90867500 21.82324200 9.23592200
C 14.52217700 23.26978900 9.37483700
H 15.29128700 23.84883000 9.90933300
H 13.58722100 23.37205400 9.94448400
H 14.36937500 23.73434300 8.39207600
O 5.74718500 24.90943500 13.97777200
H 5.08082600 25.60790200 13.94984400
O 7.47067700 23.04913200 14.18659200
O 10.79626900 22.66486700 12.95212900
O 12.45736600 24.25627900 11.91128500
H 13.08447800 24.84121100 11.46710600
P 3.45044500 22.82919900 15.78266200
P 8.27418000 23.54476500 10.50057100
P 9.95364600 25.46374200 15.47213500
P 14.89465000 21.65023300 12.05835100
Pd 5.52238400 22.88932800 14.93272000
Pd 6.89070700 24.18204900 12.20955300
Pd 7.74875900 25.29365900 14.86320200
Pd 10.49140800 23.81834600 11.05999400
Pd 11.31410800 24.81889200 13.74566500
Pd 12.75416100 22.17586100 12.52406600
S 9.09587100 22.56705800 13.69989700

Hexamer Z = P, X = O (7c)

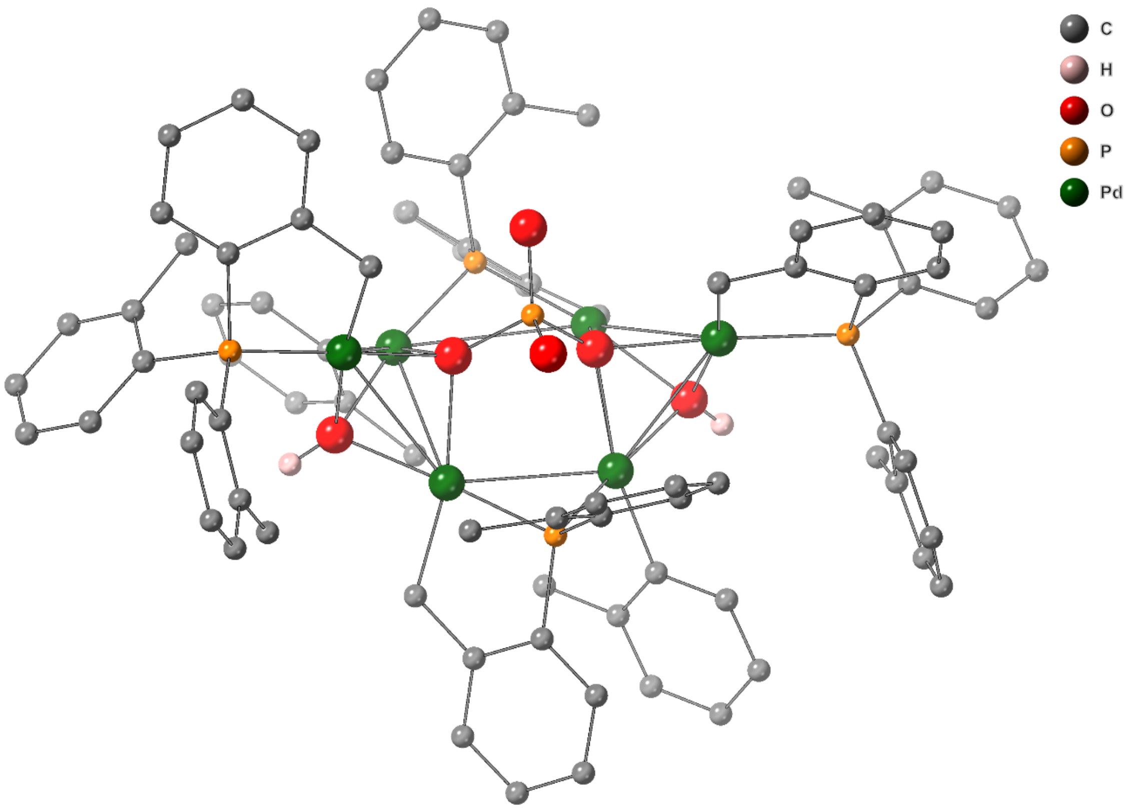

Sum of electronic and zero-point Energies= -6172.626260

Sum of electronic and thermal Energies= -6172.523845

Sum of electronic and thermal Enthalpies= -6172.522900

Sum of electronic and thermal Free Energies= -6172.775303

0 2
C 6.48701747 21.55391355 15.89372813
H 6.42423048 20.73910151 15.15200911
H 7.53869656 21.65670655 16.20170119
C 5.57023942 21.29543656 17.05574125
C 5.95781942 20.57311945 18.19519931
H 6.97830351 20.18762346 18.26514633
C 5.05171636 20.35951849 19.23876838
H 5.37076638 19.80426841 20.12477144
C 3.74471727 20.85945552 19.16666237
H 3.04767022 20.69698651 19.99215845
C 3.34476424 21.58973356 18.04470230
H 2.34044317 22.01731857 17.99015530
C 4.25954131 21.80462355 17.00860320
C 2.94990421 21.88997159 14.37390006
C 2.29379117 20.73838950 14.83770206
H 2.37960117 20.44959345 15.88549614
C 1.53693311 19.94567145 13.97314402
H 1.03383107 19.05384835 14.35392304
C 1.43602610 20.29890547 12.62686789
H 0.85095906 19.68524142 11.93737988
C 2.09000715 21.44045054 12.15830985
H 2.01302714 21.71234758 11.10239682
C 2.85555920 22.25380561 13.00716096
C 3.56173426 23.45388070 12.44090590
H 3.29506224 23.60188872 11.38534884
H 4.65316033 23.32778570 12.50502393
H 3.31596824 24.37739777 12.98733491

C 3.04059622 24.31113774 16.05932415
C 1.81260713 24.60451879 15.44533413
H 1.40208310 23.91925870 14.70181607
C 1.10719608 25.76327885 15.77454815
H 0.15332801 25.97727887 15.28673410
C 1.63203912 26.64037691 16.72540320
H 1.09477808 27.55532999 16.98716422
C 2.84732021 26.34653288 17.34841924
H 3.25167824 27.03368595 18.09602530
C 3.57427826 25.18684280 17.04120825
C 4.86349735 24.90599282 17.76316826
H 5.14069137 25.74948384 18.40858935
H 5.68784441 24.71850775 17.05503025
H 4.78345534 24.00834675 18.39466135
C 6.34632448 25.29878484 10.71848878
H 5.30737237 24.97054281 10.88763579
H 6.40216845 26.37941289 10.92804376
C 6.76174047 24.97598482 9.31098768
C 6.28364947 25.68492585 8.19872960
H 5.61945741 26.54051889 8.34834060
C 6.64002050 25.29249981 6.90595948
H 6.26196643 25.85373588 6.04762144
C 7.47154851 24.18244072 6.69894248
H 7.74314254 23.88247173 5.68430040
C 7.96968158 23.47627067 7.79223157
H 8.64273064 22.62851861 7.64945558
C 7.62771057 23.89109372 9.08730567
C 8.55352559 21.45924152 10.44891975
C 9.83710973 20.95568852 10.19407275
H 10.68626575 21.63880057 10.14081175
C 10.03684270 19.58327642 10.04199271
H 11.04024278 19.19864637 9.84828071
C 8.94750863 18.71615637 10.15413971
H 9.09131366 17.63829529 10.04374471
C 7.67056758 19.22088838 10.42082075
H 6.82813550 18.53223934 10.52746177
C 7.44529354 20.59430946 10.58118178
C 6.08693846 21.11599853 10.96242377
H 5.75169139 21.92749655 10.29743175
H 6.12127746 21.52396556 11.98634688
H 5.33012736 20.31949448 10.93751778
C 7.56337955 27.58901196 15.47634412
C 6.87651251 27.82052500 16.67749520
H 6.61001948 26.97899093 17.31787624
C 6.54271046 29.11761209 17.08819125
H 6.01042245 29.27075610 18.03162729
C 6.90817049 30.20793419 16.29649716
H 6.66868450 31.22748423 16.61054422
C 7.58980153 29.98642118 15.09529507
H 7.87850757 30.83966123 14.47354202
C 7.92105956 28.69116408 14.66638305
C 8.63506662 28.48912304 13.35548199
H 9.45597870 27.76372099 13.45179399
H 9.04416265 29.43285812 12.96417092
H 7.94749757 28.08060503 12.59710590
C 12.19929389 27.06473595 14.31882805
H 13.22811696 26.76832091 14.60126805
H 12.26208691 27.55954599 13.33241896
C 11.63931185 27.99930700 15.36105413
C 12.08568588 29.32410309 15.51201813
H 12.83086593 29.72362614 14.81744505
C 11.60098582 30.12247619 16.55011520
H 11.95731685 31.15076924 16.65593922
C 10.66915475 29.60908912 17.46433224
H 10.29662275 30.23453815 18.27929330
C 10.20009775 28.30495105 17.31412723
H 9.45296070 27.90372600 18.00124727
C 10.66596577 27.51813999 16.25177517
C 10.07214073 24.89903578 17.36253224
C 8.85750363 24.48925378 17.93261330
H 7.92663756 24.73785479 17.42018726
C 8.82641462 23.75381572 19.11762839
H 7.86862656 23.43825267 19.53915738
C 10.02899474 23.41222170 19.74053944
H 10.02602473 22.83042566 20.66614347
C 11.24330180 23.80733973 19.17219836
H 12.18223088 23.52622569 19.65759439
C 11.29467983 24.55007878 17.98261330
C 12.62461491 24.92329480 17.38595627
H 12.70455291 24.54960374 16.35160917
H 13.45358796 24.50685776 17.97597229
H 12.75534090 26.01604989 17.33337627
C 10.49240775 25.00725681 9.57927668
C 11.18997883 24.25297973 8.62862261
H 11.54070285 23.24843468 8.87150465
C 11.43421884 24.76912477 7.35073354
H 11.96982787 24.16409473 6.61560749
C 10.97422876 26.04635989 7.02252553
H 11.14214581 26.45405990 6.02227644
C 10.29497076 26.80403292 7.97999556
H 9.94226672 27.80827899 7.72603356
C 10.04693274 26.30562590 9.27073465

C 9.35483166 27.16319197 10.29602372
H 8.80213862 26.54855789 11.01792182
H 8.66116664 27.87907301 9.83019973
H 10.09165070 27.74353701 10.87686576
C 11.93639487 20.67505847 13.39605999
H 11.86269286 20.82714052 14.48692703
H 10.92178576 20.43489449 13.04021194
C 12.93395691 19.60010341 13.07234295
C 12.74916694 18.26093831 13.46184196
H 11.83703687 17.97924228 13.99476599
C 13.72444299 17.30316824 13.18236393
H 13.56976395 16.26708416 13.49497898
C 14.90574505 17.65778229 12.51212691
H 15.66791915 16.90251121 12.30619986
C 15.10070109 18.97746039 12.10866085
H 16.01264516 19.26707936 11.58110283
C 14.10806401 19.93076342 12.37974989
C 15.81182012 22.28947660 12.40187892
C 16.88816823 22.13528657 11.51230683
H 16.71659923 21.69758856 10.52700176
C 18.17444831 22.54455961 11.86460886
H 18.99993836 22.41800359 11.16024579
C 18.39005930 23.11848266 13.11909396
H 19.39000237 23.45102569 13.40882596
C 17.32482127 23.26544868 14.00944902
H 17.50185826 23.70935071 14.99249509
C 16.02195317 22.85420763 13.68366099
C 14.92364505 23.02635163 14.70091906
H 15.32338708 23.45131070 15.63209411
H 14.43647506 22.06995959 14.94613710
H 14.12864202 23.69525268 14.33350002
C 14.15012899 21.71951656 10.05300071
C 13.76419600 20.55886146 9.35880165
H 13.54802699 19.64455843 9.91250071
C 13.65048698 20.56086847 7.96888157
H 13.34377796 19.65160543 7.44679852
C 13.93640700 21.72854956 7.25644352
H 13.85904499 21.74273458 6.16649243
C 14.30940605 22.88423563 7.94236360
H 14.51317205 23.80182972 7.38520652
C 14.40606001 22.91516964 9.34221866
C 14.77607604 24.21462375 10.00923574
H 15.86405012 24.38740478 9.96731373
H 14.49220404 24.24289472 11.06828081
H 14.28669604 25.05642378 9.49622168
O 6.12460842 25.23662980 13.80930900
H 5.39525337 25.85570485 13.67487596
O 8.07541056 23.65068368 14.18104104
O 10.41747672 23.34390868 13.38708895
O 12.18928990 24.70685278 12.14608390
H 12.78240890 25.24144579 11.60355683
P 4.00106529 22.84506265 15.53675510
P 9.26283366 22.49802863 14.21992604
P 8.35495458 23.25263767 10.60946376
P 10.01672471 25.87010588 15.80834115
P 14.15987803 21.68122556 11.88732284
Pd 6.08843545 23.26573766 14.86106407
Pd 7.33724153 24.38043773 12.25030790
Pd 7.95306859 25.72493483 14.91463205
Pd 10.22734672 24.21504577 11.39145481
Pd 11.26276980 25.28547482 14.04049003
Pd 12.28242991 22.55313461 12.71014594
O 8.78703865 21.33993454 13.37215096
O 9.74502669 22.29903862 15.63634415

Hexamer Z = P, X = OH (7d)

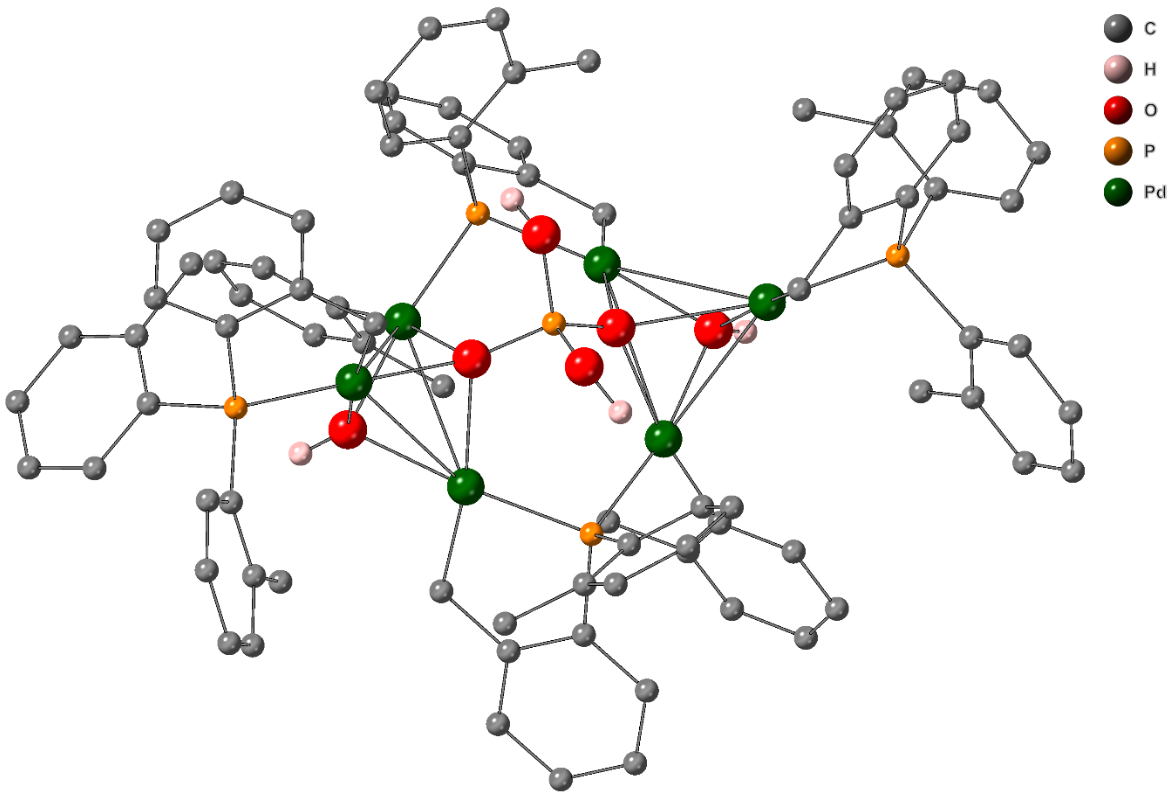

Sum of electronic and zero-point Energies= -6173.824190

Sum of electronic and thermal Energies= -6173.720329

Sum of electronic and thermal Enthalpies= -6173.719385

Sum of electronic and thermal Free Energies= -6173.976679

0 2
C 5.99874000 21.31579600 15.81391400
H 5.79572500 20.55138400 15.04219200
H 7.06510000 21.25736000 16.08038600
C 5.11893300 21.10458800 17.01679600
C 5.46021700 20.26708900 18.08898400
H 6.41731300 19.73893800 18.07766300
C 4.58803900 20.11687700 19.17251600
H 4.87144900 19.47014400 20.00689600
C 3.36252400 20.79428200 19.20510600
H 2.69352800 20.67887700 20.06099600
C 3.00859900 21.63706500 18.14812100
H 2.06971800 22.19573800 18.17221600
C 3.88997300 21.78681200 17.07304000
C 2.50790400 22.13983300 14.49345900
C 1.79408800 21.00422100 14.90970900
H 1.91324400 20.62970300 15.92685900
C 0.93429600 20.33828400 14.03466200
H 0.38658800 19.45712000 14.37650100
C 0.78793500 20.80397300 12.72738600
H 0.12195600 20.29009400 12.02984600
C 1.49860200 21.93053800 12.30718200
H 1.38334500 22.29112900 11.28181900
C 2.36792700 22.61924600 13.16609100
C 3.11721800 23.81771100 12.65421100
H 2.85370100 24.02443400 11.60807800
H 4.20461100 23.65808000 12.70571500
H 2.90027500 24.72205200 13.24436900

C 2.91000300 24.45322500 16.28498600
C 1.64946300 24.84448700 15.80562000
H 1.12000800 24.21025000 15.09272900
C 1.06300300 26.03737300 16.23087000
H 0.08199900 26.32833000 15.84842900
C 1.74006300 26.84938100 17.14251900
H 1.29588800 27.78964200 17.47824800
C 2.98831700 26.45792500 17.63220200
H 3.51103200 27.09391200 18.35109700
C 3.59851900 25.26212600 17.22649900
C 4.93666500 24.88046700 17.79580700
H 5.28836900 25.63676600 18.50972500
H 5.68901000 24.78017500 16.99634600
H 4.89580700 23.91202900 18.31652500
C 6.04242900 25.51356800 10.77659100
H 4.97844800 25.24344800 10.89529100
H 6.15853200 26.56400600 11.09822300
C 6.46834600 25.32973600 9.34041100
C 5.91546300 26.08226100 8.29052300
H 5.17521500 26.85638500 8.51320100
C 6.29492400 25.83744700 6.96886800
H 5.85613900 26.42874600 6.16079400
C 7.22813400 24.83377500 6.67114700
H 7.51943600 24.64390300 5.63533700
C 7.79917800 24.09119900 7.70380500
H 8.54623700 23.32542900 7.48779200
C 7.42651600 24.35269700 9.02926000
C 8.04397900 21.76356800 10.29463900
C 9.23590200 21.00975100 10.27046900
H 10.19681600 21.52980400 10.31969000
C 9.20861900 19.61319900 10.21001200
H 10.14557100 19.05230200 10.20872800
C 7.97830000 18.95522800 10.16215700
H 7.93792200 17.86408500 10.12015500
C 6.79252100 19.69477300 10.17713600
H 5.83284100 19.17195000 10.14787900
C 6.79177500 21.09663600 10.24606900
C 5.48543600 21.83814900 10.30552400
H 5.37670700 22.53968700 9.46382500
H 5.42913600 22.44453700 11.22444900
H 4.63468200 21.14286000 10.29177200
C 7.63151300 27.45913000 15.42995200
C 6.97992400 27.80496900 16.62048200
H 6.65551900 27.02585100 17.31176400
C 6.74676600 29.14763600 16.94604600
H 6.23584600 29.40023700 17.87951000
C 7.18057300 30.15321000 16.08078100
H 7.01697000 31.20541300 16.32796700
C 7.82938900 29.80783000 14.89061400
H 8.16745700 30.59625500 14.21131800
C 8.06342600 28.46810300 14.54006900
C 8.75924100 28.12842400 13.24680900
H 9.66670800 27.53035000 13.42362100
H 9.04411500 29.03462800 12.69209300
H 8.11578800 27.51394100 12.59747000
C 12.25817000 26.73100400 14.40867500
H 13.26120500 26.34266800 14.66697300
H 12.35565600 27.29999400 13.46767300
C 11.74342300 27.60720000 15.52411600
C 12.28292600 28.87735500 15.79188200
H 13.07247500 29.27312700 15.14636900
C 11.82971200 29.62565700 16.87996000
H 12.25862700 30.61206400 17.07551900
C 10.83497000 29.11742100 17.72816600
H 10.48725100 29.70507000 18.58119900
C 10.27460900 27.86847700 17.46452900
H 9.48006400 27.47302500 18.10023500
C 10.71613800 27.13203600 16.35676300
C 9.97729600 24.40587000 17.19290400
C 8.74384600 24.02992800 17.76430500
H 7.82732900 24.46612300 17.36393200
C 8.67549900 23.10055900 18.80335200
H 7.70576200 22.81649300 19.21818700
C 9.85616300 22.53655500 19.29125500
H 9.82316200 21.80083800 20.09860300
C 11.08593900 22.90737400 18.74089700
H 12.00508200 22.45734000 19.12462000
C 11.18024600 23.83841300 17.69418500
C 12.52423200 24.18048900 17.11633000
H 12.55899200 23.92946800 16.04340100
H 13.32534100 23.63342000 17.63189000
H 12.73306700 25.25912800 17.19154300
C 10.63278600 24.81785800 9.29036600
C 11.21778500 23.97555700 8.33489900
H 11.43641000 22.93446300 8.58397200
C 11.52749800 24.45291700 7.05516600
H 11.98269900 23.78222400 6.32107700
C 11.24446500 25.78005000 6.72480600
H 11.47217100 26.16258800 5.72632500
C 10.66286300 26.62099900 7.67933800
H 10.44020400 27.66045300 7.41954900
C 10.34707000 26.16220000 8.96867300

C 9.71670700 27.08712700 9.97818500
H 8.81171500 26.63926000 10.41458500
H 9.44414400 28.05338100 9.52789300
H 10.40117200 27.28282500 10.81976300
C 12.35993000 20.19816900 12.57643300
H 11.46051300 19.83336600 13.09942700
H 12.29651000 19.86655500 11.52250200
C 13.60798900 19.69058800 13.20533200
C 13.63777800 18.63643500 14.14002700
H 12.69484500 18.16553100 14.43383100
C 14.83983300 18.18057100 14.68725100
H 14.82741000 17.36062800 15.41121300
C 16.05890100 18.76057300 14.31124200
H 16.99902900 18.40036800 14.73652700
C 16.05796600 19.82426100 13.40382000
H 16.99834600 20.30987700 13.13083300
C 14.85228600 20.29914700 12.87364100
C 16.19570800 22.80674700 12.33080600
C 17.27895300 22.92873800 11.44399300
H 17.26052700 22.38703000 10.49621800
C 18.38104500 23.72765600 11.75537200
H 19.21399500 23.80578300 11.05194600
C 18.41056500 24.41809000 12.96920100
H 19.26512300 25.04962700 13.22532600
C 17.34025100 24.29816400 13.85991200
H 17.36645500 24.83771000 14.81079400
C 16.22201300 23.50235200 13.56875200
C 15.09866100 23.40151700 14.56683300
H 15.26450900 24.08203800 15.41404600
H 15.00074500 22.37841500 14.96511200
H 14.12675000 23.64208600 14.10477900
C 14.93168500 21.37511800 10.13182200
C 15.14753300 20.03577300 9.77115900
H 15.27234600 19.28424900 10.55345300
C 15.20133600 19.65141200 8.42877600
H 15.37007400 18.60340700 8.16845200
C 15.03912900 20.61268800 7.42931000
H 15.07706800 20.32569600 6.37530600
C 14.82343400 21.94886300 7.78043200
H 14.69285000 22.70019100 6.99688400
C 14.76018600 22.35645100 9.12044300
C 14.50892400 23.80150100 9.45569000
H 15.37079900 24.25353200 9.97223400
H 13.64386400 23.89667000 10.12939900
H 14.30382400 24.38948500 8.55121700
O 5.93170400 25.14958500 13.97517100
H 5.22089500 25.80302400 13.96724600
O 7.82758100 23.32688100 14.11703800
O 10.21545200 23.10744300 13.26351200
O 12.16796600 24.56652300 12.11221300
H 12.82408000 25.14279300 11.70106700
P 3.68353700 22.91977900 15.66250900
P 9.07635500 22.40177800 14.00437200
P 8.18070600 23.59010200 10.50137500
P 9.96583900 25.58111400 15.76554100
P 14.69888100 21.83290700 11.89301600
Pd 5.76777900 23.12943500 14.90400900
Pd 6.96821700 24.40784700 12.20135300
Pd 7.85983000 25.55988900 14.93406300
Pd 10.28655500 24.10514300 11.10314800
Pd 11.21585200 25.04580300 13.98084600
Pd 12.46958600 22.30997700 12.36021600
O 8.68657900 21.01009800 13.33293200
H 8.82520300 20.98572700 12.36291700
O 9.49924900 21.88313000 15.45522700
H 9.60972400 22.60355000 16.11016500

7. References

[1] C. Liu, G. Meng, M. Szostak, M. *J. Org. Chem.* **2016**, *81*, 12023.

[2] G. Athavan, T. F. N. Tanner, A. C. Whitwood, I. J. S. Fairlamb, R. N. Perutz, *Organometallics* **2022**, *41*, 3175.

[3] A. C. Albéniz, P. Espinet, O. López-Cimas, B. Martín-Ruiz, *Chem. Eur. J.* **2005**, *11*, 242.

[4] CrysAlisPro. CrysAlisPro, Oxford Diffraction Ltd. Version 1.171.34.41. p Oxford Diffraction Ltd. Version 1.171.34.41.

[5] Empirical absorption correction using spherical harmonics implemented in SCALE3 ABSPACK scaling algorithm within CrysAlisPro software. Scale3. Empirical absorption correction using spherical harmonics, implemented in SCALE3 ABSPACK scaling algorithm within CrysAlisPro software, p Oxford Diffraction Ltd. Version 1.171.34.40.

[6] O. V. Dolomanov, L. J. Bourhis, R. J. Gildea, J. A. K. Howard, H. Puschmann, *J. Appl. Crystallogr.* **2009**, *42*, 339.

[7] G. M. Sheldrick, *Acta Cryst. A* **2015**, *71*, 3.

[8] C. Lee, W. Yang, R. G. Parr, *Phys. Rev. B* **1988**, *37*, 785.

[9] A. D. Becke, *J. Chem. Phys.* **1993**, *98*, 5648.

[10] P. J. Stephens, F. J. Devlin, C. F. Chabalowski, M. J. Frisch, *J. Phys. Chem.* **1994**, *98*, 11623.

[11] M. J. Frisch, G. W. Trucks, H. B. Schlegel, G. E. Scuseria, M. A. Robb, J. R. Cheeseman, G. Scalmani, V. Barone, B. Mennucci, G. A. Petersson, H. Nakatsuji, M. Caricato, X. Li, H. P. Hratchian, A. F. Izmaylov, J. Bloino, G. Zheng, J. L. Sonnenberg, M. Hada, M. Ehara, K. Toyota, R. Fukuda, J. Hasegawa, M. Ishida, T. Nakajima, Y. Honda, O. Kitao, H. Nakai, T. Vreven, J. A. Montgomery Jr., J. E. Peralta, F. Ogliaro, M. Bearpark, J. J. Heyd, E. Brothers, K. N. Kudin, V. N. Staroverov, R. Kobayashi, J. Normand, K. Raghavachari, A. Rendell, J. C. Burant, S. S. Iyengar, J. Tomasi, M. Cossi, N. Rega, J. M. Millam, M. Klene, J. E. Knox, J. B. Cross, V. Bakken, C. Adamo, J. Jaramillo, R. Gomperts, R. E. Stratmann, O. Yazyev, A. J. Austin, R. Cammi, C. Pomelli, J. W. Ochterski, R. L. Martin, K. Morokuma, V. G. Zakrzewski, G. A. Voth, P. Salvador, J. J. Dannenberg, S. Dapprich, A. D. Daniels, O. Farkas, J. B. Foresman, J. V. Ortiz, J. Cioslowski, D. J. Fox, Gaussian16 (Revision A.03), Gaussian Inc. Wallingford CT;

[12] A. V. Marenich, C. J. Cramer, D. G. Truhlar, *J. Phys. Chem. B* **2009**, *113*, 6378.

[13] S. Grimme, S. Ehrlich, L. Goerigk, *J. Comput. Chem.* **2011**, *32*, 1456.
